# Supplementary material for: Efficient sampling of large-scale transition pathways and intermediate conformations in sub-mesoscopic protein complexes
Source: Nat Commun. 2026 Mar 2;17:2202. doi: 10.1038/s41467-026-69809-y (PMC12960823; doi:10.1038/s41467-026-69809-y)
Supplement: Supplementary file 1 — Supplementary Information [file 41467_2026_69809_MOESM1_ESM.pdf]

Supplementary Information

Efficient sampling of large-scale transition pathways and intermediate conformations in sub-mesoscopic protein complexes

Domenico Scaramozzino[1], Byung Ho Lee[1], Laura Orellana[1,*]

[1]Protein Dynamics and Mutation Lab, Department of Oncology-Pathology, Karolinska Institutet, Solna, Sweden.

*Corresponding author: laura.orellana@ki.se

Generation of the protein datasets

In this work, we investigated in detail the conformational diversity and transition pathways of several proteins. Three of these systems (Supplementary Table 1), i.e., ribose-binding protein (RBP, 30 kDa), RNA endonuclease III (RNaseIII, 48 kDa), and sarcoplasmic/endoplasmic reticulum Ca^{2+} ATPase1 (SERCA, 109 kDa), are of medium-size and come from our previous eBDIMS benchmark[1]. These proteins have been used here to test the impact of two main parameters in eBDIMS2, to assess the performance of our method against other existing path-sampling algorithms, as well as to compare against Molecular Dynamics (MD) sampling.

In order to build a comprehensive dataset of larger proteins undergoing large-scale conformational changes, we performed a far-reaching bioinformatic search combining the Protein Data Bank (PDB)[2] and the UniProt database[3]. First, we retrieved all UniProt IDs that satisfy the following criteria: (i) reviewed entries; (ii) monomeric sequence of at least 500 amino acids; (iii) at least 3 structural models from cryogenic Electron Microscopy (cryo-EM). This provided a list of 1,376 UniProt candidates. Then we screened the PDB for structures satisfying the following conditions: (i) model obtained from cryo-EM; (ii) resolution < 5 Å; (iii) molecular weight in the range 400 kDa – 3 MDa. This generated a list of 6,114 PDB codes. Cross-referencing the two lists from UniProt and PDB, we narrowed down our search to 855 UniProt candidates. Note that, in this stage, we looked for cryo-EM models because these are the most common types of structural data for large proteins captured in different conformations. However, in the subsequent steps, both cryo-EM and X-ray structures were considered for the ensemble generation. Then, we applied the condition that every entry should contain at least 3 models with: (i) good-to-medium resolution (< 5 Å); (ii) large molecular weight (400 kDa – 3 MDa); (iii) number of modelled residues in the range 3,000 – 30,000. Note that the availability of at least 3 models is the minimum condition to build a structural ensemble meaningful for 2D Principal Component Analysis (PCA, see below). The lower limits of molecular weight and modelled residues allowed to discard small- and medium-size proteins, whereas the upper limit was used to exclude extremely large assemblies like microtubules or filaments. This reduced our UniProt list to 511 candidates (Supplementary Table 2).

In the second stage of our search, we implemented additional criteria to retrieve ensembles of large proteins, discarding protein-protein assembly machines and ensuring enough conformational diversity. A proper protein ensemble should contain at least 3 PDB structures that contain a maximum of 10 different polymer entries. This condition allowed us to exclude gigantic assemblies of proteins whose behavior is driven by protein-protein interactions and compositional changes, rather than the conformational dynamics of individual entities. We looked for cases where there were at least two structures with a Root Mean Square Deviation (RMSD) > 4 Å. This condition ensures that the ensemble has an acceptable degree of conformational diversity. We used US-Align[4] to align PDB models and compute RMSD values. At the end of this stage, we narrowed down our list to 158 candidates (Supplementary Table 3).

Finally, we manually went through each of the 158 retained entries and retrieved all high-quality PDB models to build structural ensembles. Certain cases where proper ensembles could not be built were excluded

from subsequent analysis. For example, for the *S. Flexneri* outer membrane protein MxiD (UniProt ID: Q04641), our search provided only 3 good PDB models: 8axk, 8axl, and 8axn. While 8axk and 8akn report the MxiD ring in a 16-mer configuration, 8axl reports MxiD in a 15-mer oligomeric state, making the generation of a coherent ensemble impossible. Most of the remaining UniProt IDs were linked to each other to form heteromeric protein complexes. For example, the *S. Cerevisiae* DNA replication licensing factor MCM complex is composed of six subunits, four of which have UniProt IDs that were independently included in our list (P24279, P30665, P53091, P38132, see Supplementary Table 3). By gathering all these inter-connected UniProt entries, we finally built independent ensembles for 40 large proteins (Supplementary Table 4, Fig. 2a in the main text). Some of these proteins were considered in multiple oligomerization states, e.g., GroEL, MCM, VCP, etc., with different interacting partners, e.g., ClpA/P, or considering different isoforms, e.g., Nf1. This finally led us to analyze 47 ensembles of large protein systems (Supplementary Table 4).

During the review process of this manuscript, we were asked to identify additional multimeric complexes exhibiting large-scale conformational transitions. To address this, we constructed a supplementary dataset of two-state proteins using the same screening approach described above, with two key modifications. First, we relaxed the requirement of at least three experimental models – previously necessary to generate a meaningful two-dimensional PC space – and instead accepted pairs of PDB structures exhibiting sufficient conformational diversity (RMSD > 4 Å). Second, we removed the constraint limiting PDB entries to a maximum of 10 distinct polymer chains. These adjustments enabled us to identify additional multimeric systems that were excluded by our initial criteria. From the resulting dataset, we excluded riboproteins (e.g., ribosomes and spliceosomes) and macromolecular complexes containing nucleosomes. While such systems are indeed compelling for studying large-scale conformational dynamics, accurate modeling of their RNA and DNA components falls beyond the scope of this work and is currently being addressed elsewhere (Cannariato et al., manuscript in preparation). After filtering out these protein–nucleic acid complexes, we were able to identify 15 additional large multimeric protein complexes exhibiting two distinct conformations (Supplementary Table 5). These complexes range in size from ~350 kDa to ~1.1 MDa and display diverse shapes, oligomeric states, and biological functions – including RNA polymerases, ionotropic receptors, mechanosensitive channels, and bacteriophage tails (Supplementary Fig. 1). The conformational transitions observed span from ~5 Å to an exceptional ~57 Å RMSD (Supplementary Table 5).

Construction of structural ensembles and Principal Component Analysis (PCA)

For the protein dataset where at least 3 experimental conformers are available, structural ensembles were generated by downloading all PDB models fulfilling the above-mentioned criteria and making sure that they are all structurally consistent. This was achieved by: (i) checking that chain labels are coherent with relative orientations of protomers in homo- and heteromeric systems; (ii) making sure that all structures have the same number of residues and that all residues belong to the same sequence positions. The last column of Supplementary Table 4 reports all PDB IDs used for generation of the ensembles. For each ensemble, a reference structure - generally associated to apo-resting state - was chosen. All structures were subsequently aligned to the reference to get rid of translational and rotational degrees of freedom (DOFs). Structural alignment was carried out using the gmx confrms tool from Gromacs[5]. Then, we carried out Principal Component Analysis (PCA) to extract apparent collective motions from the aligned ensembles.

Supplementary Table 6 and Supplementary Fig. 2 report the PCA results, showing the variance captured by the first two principal components (PCs), and the total number of PCs needed to describe at least 90% of the variance. The variance covered by PC1 ranges from a minimum of ~41% (MCM 6mer) to a maximum of ~99.9% (Nf1 isoform 2), with an average of ~75%. The variance covered by PC2 ranges from ~0.1% (Nf1 isoform 2) to a maximum of ~44% (*S. Oleracea* ATP synthase), with an average of ~17% (Supplementary Fig. 2a). For the majority of ensembles (33/47), PC1 and PC2 alone cover more than 90% of the variance. An additional PC is needed in 9 systems, while in the remaining cases, 4-7 PCs should be considered to reconstruct >90% of the variance (Supplementary Fig. 2b). PC1 and PC2 always allow to capture at least ~70%. Our PCA

results suggest that these large systems undergo large-scale motions so that their conformational diversity can be explained by just a few PC eigenmodes. Based on the projections of all experimental structures in the PC space, we can detect relevant conformational clusters and select representative end-state conformations to simulate transitions with eBDIMS2.

eBDIMS2 code development, testing, and additional features

eBDIMS2 exploits the fact that, in the essential-dynamics Elastic Network Model (edENM)[6], non-bonded interactions exhibit a strong decay for increasing particle-particle distances, i.e.:

$$k_{ij} = \left(\frac{6}{r_{ij}}\right)^6 \quad \text{(S1)}$$

where k_{ij} is the spring constant (in kcal/molÅ^2) between particles i and j in the ENM, and r_{ij} is their Cartesian distance (in Å). Notice that if the distance between two residues is greater than, e.g., 8 Å, their interaction force becomes so weak that it can in principle be ignored. Instead of considering all $(N^2 - N)/2$ pairwise interactions (see Eq. 6 in the main text) like in our previous algorithm[1,7], we implemented a more efficient approach based on an adaptive spatial cutoff r_c, similar to what is done in Molecular Dynamics (MD) simulations. Every 1,000 steps along the Brownian Dynamics (BD) simulation, we generate a list L of interacting pairs ($d_{ij} < r_c$), where residue-residue distances d_{ij} are evaluated based on the updated coordinates of C^α atoms. This list is used to calculate new interacting forces at each BD step (Eq. 7 in the main text). In this way, we reduce the number of iterations to be performed at each step, resulting in a much more efficient simulation.

The lower r_c, the lower the number of residue-residue interactions, resulting in a faster simulation especially for larger proteins. Yet, this might come at the expense of ignoring potentially relevant contributions to the total interacting force. To look for optimal values of r_c, we tested the performance of eBDIMS2 by using four cutoff values, i.e., 8, 10, 15, and 20 Å. We looked both at the computational efficiency to achieve transition convergence and at the accuracy in predicting the transition pathway. All calculations were performed on a Linux workstation with an Intel® Core i9-13900K processor and using OpenMP parallelization on 16 threads. Supplementary Figs. 3 and 4 show the results for the transitions for RBP, RNaseIII, SERCA, and GroEL 7-mer. In this range of cutoff values, r_c does not significantly modify the PC projection of the transitions (Supplementary Fig. 4), hence it does not impact the pathway accuracy. However, it does play a role for computational efficiency (Supplementary Fig. 3). On average, smaller cutoffs (8-10 Å) lead to the lowest computing times, especially as the scale of the system increases (Supplementary Figs. 3c-d). A value of r_c = 8 Å was selected as optimal and used for all subsequent calculations.

While r_c controls the amounts of force calculations to be performed at each BD step, the biasing frequency k is used to check the outcomes of the BD simulation periodically and drive the pathway towards the target[1]. Hence, k can also have a profound impact on the method performance. Supplementary Figs. 3 and 4 show the computing times and projections of the eBDIMS2 transitions also as a function of four distinct values of the biasing frequency, i.e., k = 1, 2, 5, and 10. The method is very robust, as it provides similar pathways for different k values in this range (Supplementary Fig. 4). However, biasing the transition every (k = 1) or every other (k = 2) step results in a loss of efficiency. Greater performance is generally achieved with k = 5 and k = 10 (Supplementary Fig. 3). Since for the largest system, k = 10 provides the highest computing speed (Supplementary Fig. 3d), we selected k = 10 as optimal.

Since conformational changes can also involve quasi-rigid rotations of entire domains and/or chains, we implemented the explicit treatment of rigid blocks as an additional feature in eBDIMS2. In these cases, using classical ENM parametrizations for non-bonded interactions might lead to artifacts along the transition pathway. This occurrence was observed, e.g., in ATP synthases (Supplementary Fig. 5), which undergo large-scale conformational cycles where the F_0 rotor domains cover a 360° rotation in three steps of ~120°. When the standard edENM is applied to these systems, the predicted pathway gives rise to an unrealistic compression of the rotor (Supplementary Fig. 5a). We fixed this issue in eBDIMS2 by increasing the spring connectivity

between all residues belonging to the rigid block, i.e., without using a cutoff for long-range interactions in the rigid block and setting all spring constant values in this region equal to 1 kcal/molÅ^2, which corresponds to much stronger connections than those employed in the edENM above an 8 Å cutoff. In this work, we have applied rigid conditions at the whole-chain level to all rotor chains of the three ATP synthases and of the V-type ATPase/synthase (Supplementary Table 7). This strategy allowed us to model all these rotors as rigid blocks with no internal deformations. The resulting transitions showed a more realistic pathway, with the F_0 subunits completing each 120° rotation with no internal deformation like in the experimental conformers (Supplementary Fig. 5b, see Fig. 4 in the main text).

Another advantage of eBDIMS2 compared to our previous C^{++} code[7] and the majority of path-sampling algorithms is that it can also deal with proteins with missing residues. This is especially important when considering large models from cryo-EM that inevitably tend to exhibit missing parts in the model. Missing regions are dealt with by ignoring bonded interactions in the portion of the sequence affected by the unmodelled amino acids, and by using long-range non-bonded interactions to guarantee the connectivity of the network before and after the gap (Supplementary Fig. 6).

In eBDIMS2 we also included the possibility to simulate transitions between end states with different numbers of residues. In this case, the correspondence between residues in the two end conformations is found by searching for correspondences between protomer chain labels and residue numberings. This works not only in the case of minor differences between sequence coverages, but also in the case of entire domains missing in one of the two end-state conformers. Supplementary Fig. 7a shows the transitions between a full-length Ca^{2}-E1 open conformation of SERCA (PDB: 2c9m, 994 residues) and a mutant Ca^{2}-E1-MgAMPPCP closed state that is completely lacking the headpiece A-domain (4nab, residues 1-46 and 123-240 missing). In the simulation from the full-length open state, the A-domain is performing small-amplitude fluctuations around its original position, while the rest of the structure undergoes a large-scale conformational change to converge into the (incomplete) target. Similarly, Supplementary Fig. 7b shows the transitions of the large ITPR3 (> 8k residues), between a quasi-complete apo state (6dqj) and an inhibited conformation where all the N-terminal domains are missing (8tla, residues 1-234 missing in each protomer). Also in this case, the NTDs of the apo state perform small-scale oscillations while the rest of the structure is able to correctly converge to the target.

This approach also enables the simulation of transitions between end states that differ in the number or type of polypeptide chains. Residue correspondence for pathway convergence is determined based on matching chain identifiers and residue numbering, allowing for greater flexibility. This enhanced capability was particularly useful in our additional dataset of 15 two-state proteins, where several complexes presented differing chain compositions between the two states (Supplementary Table 5). For example, the MDa-scale integrator–PP2A complex was found to include INST3, NABP2, INIP, and POLR2A in one state (PDB: 8rbz), but not in the other (8rc4). eBDIMS2 can handle such cases by making the common chains converge during the transition pathway, while non-shared protomers generally undergo unconstrained, free-vibration motions.

Application of eBDIMS2 to large proteins: large-scale transition pathways, convergences, computing times, motion complexity, and approaching experimental intermediates

To simulate transition pathways for the large proteins in our ensemble dataset, all relevant end-state conformations were selected from the projections in the 2D PC space. For each conformational cluster, we selected as relevant end-state conformer the structure with the best experimental resolution, and we tried to prioritize transitions between models that have been deposited by the same research group and in the same publication. We also carefully checked the literature to assign functional states to different conformers. Out of the 872 experimental structures used in our 47 ensembles, we selected a total of 124 relevant end states, we simulated 191 transition pathways (Supplementary Table 7), and computed projections on all experimental PC spaces (Supplementary Figs. 8-12). Except for the three ATP synthases where the conformational cycles associated to ATP synthesis are known to be directional, we explored both the forward ($R_0 \rightarrow R_t$) and backward ($R_t \rightarrow R_0$) transitions. In the cases of all ATP synthases, as well as in the case of *T. Thermophilus* V-type

ATPase/synthase, we also applied rigid constraints to all rotor chains in order remove internal deformations within the rotating F_0 domain (Supplementary Fig. 5).

For each pathway, we computed RMSD and collectivity values of the conformational change (Eqs. 8-9 in the main text), RMSD values from the target state at convergence, and the computing time needed to reach convergence. All simulations were performed on a Linux workstation with an Intel® Core i9-13900K processor, 64 GB of RAM, and using 16 OpenMP parallelization threads. The convergence of the simulations was often achieved when one of two conditions was met: (i) RMSD from the target < 0.8 Å; (ii) convergence of the Γ parameter to 99.9%. In just a few cases, we manually increased the convergence thresholds to have a longer sampling of the transition, e.g. Γ convergence of 99.95% for Nf1, or decreased them to avoid spending an excessive amount of time to refine the last steps of the pathway, e.g. Γ convergence of 99.5% for ATP synthases. Supplementary Tables 7 and 8 summarize the results of all transition pathways for the ensemble dataset and the additional set of 15 two-state proteins, respectively, reporting RMSD values, collectivity degrees, and computing times. As shown, eBDIMS2 consistently achieves high levels of convergence (<1 Å RMSD), even for complex, large-amplitude motions such as the ~23 Å transition observed in isoform 2 of neurofibromin (Nf1). Out of more than 200 simulated transitions, encompassing a wide variety of protein sizes, shapes, oligomerization states, and motion complexities, we observed only one instance of poor convergence. This occurred in the exceptionally large transition of the low-density lipoprotein receptor-related protein 2 (LRP2, ~840 kDa), with an overall RMSD of ~57 Å, and only in the closed-to-open direction (Supplementary Table 8). In this unique case, the compactness of the closed conformation (PDB: 8em7), coupled with the presence of long disordered linkers between mobile domains absent in the open structure (8em4), hindered full opening, resulting in a final ~15 Å deviation from the target (~75% of the transition covered, Supplementary Fig. 13). In contrast, starting from the open (more flexible) conformation, the absence of these disordered segments facilitated convergence of the moving domains to their positions in the closed conformation, enabling nearly complete domain rearrangements with a final RMSD of ~4 Å from the target. For all simulated transitions, we also generated animated movies in GIF format, which are freely available on figshare[8] (see Data Availability in the main text).

We investigated the relationship between the computing time (CT) required by eBDIMS2 to simulate the 191 transition pathways in our protein ensemble dataset and three fundamental variables that characterize the transition of each system, i.e., protein size, transition RMSD, and collectivity of the conformational change (Supplementary Table 7). When we look at the whole dataset, we observe a strong positive correlation between the CT and the sizes, with Pearson Correlation Coefficients (PCCs) of ~0.89, showing an almost linear relationship between computing times and system size (Fig. 2c in the main text). This correlation slightly decreases if we focus on systems with N < 10k residues, i.e., not including RyR1, RyR2, and FAS 12-mer. In this case, the PCC reaches a value of ~0.67 yet maintaining a quasi-linear relationship between times and size (Fig. 2c in the main text). Overall, these results suggest that, especially for the not extremely large systems (< 1 MDa), several features other than the system size can affect the computing time to simulate the transition. Not surprisingly, we observed a positive correlation between the size-normalized computing time (CT/N) and the RMSD of the transition, with a PCC of ~0.5. This confirms that, size being equal, larger transitions require more time to be simulated (see the Nf1 and ATP synthase outliers in Fig. 2c in the main text, right panel). Interestingly, we also found a slightly negative correlation between the size-normalized computing time (CT/N) and the collectivity of the conformational change, with a PCC value of ~-0.2. This suggests that, size being equal, a collective transition is easier to simulate than a localized one, which stems from the capability of ENMs to describe large-scale global motions better.

ENMs have demonstrated particular efficacy in capturing global opening-closing motions[9]. Leveraging this strength, eBDIMS2 has enabled the accurate simulation of such transitions in well-characterized systems, including the GroEL chaperonin (Fig. 2d in the main text), as well as in proteins undergoing more extensive opening-closing conformational changes, such as α-2-macroglobulin (A2M, Fig. 3a). These simulations routinely achieve high convergence to the target conformers (Supplementary Table 7). Yet the capabilities of eBDIMS2 extend beyond canonical opening-closing motions. By integrating ENMs with the BD simulation

framework and employing the optimized cutoff scheme, eBDIMS2 facilitates the simulation of large-scale and topologically complex transitions in high-molecular-weight systems within tractable times. For instance, we have successfully modeled the complete rotary motion of ATP synthases (>500 kDa), which encompass rigid-body rotation of the rotor domains coupled with cyclic opening-closing of the three *β*-subunits (Fig. 4 in the main text). Similarly, we captured the extensive roto-translational displacement (>23 Å RMSD) of the GTPase-activating protein-related domains (GRDs) in neurofibromin (Nf1, ~530 kDa; Supplementary Fig. 31), as well as the gigantic conformational rearrangement (~57 Å RMSD) in the open-to-closed transition of the lipoprotein receptor-related protein 2 (LRP2, ~840 kDa) expected to occur in response to pH changes (Fig. 3b in the main text). Additional cases include the ~18 Å shear transition in macrophage-expressed gene 1 protein (Mpeg1, ~940 kDa), wherein a twisted pore structure shifts into a closed ring configuration (Fig. 3c), the torsional twisting (~18 Å) observed in the *Escherichia Phage Lambda* tail tip complex (~1.1 MDa, Fig. 3d), etc. Collectively, these examples highlight the versatility and robustness of eBDIMS2 in modeling a diverse array of conformational transitions across a wide spectrum of protein architectures and sizes.

We and others have previously shown that transition pathways connecting stable end states from X-ray can sample the crystallographic motions and spontaneously predict on-path experimental intermediates[1,10]. To assess whether this also holds true for larger cryo-EM systems, we have evaluated how close eBDIMS2 paths approach experimental intermediates. The identification of intermediate states is straightforward if we use projections of structural ensembles on the low-dimensionality spaces defined by the lowest PCs. From our large protein ensemble benchmark we have selected six systems for which this identification was rather straightforward: the two spike glycoproteins from *SARS-CoV* and *SARS-CoV-2* (Supplementary Fig. 8), DNA-PKcs (Supplementary Fig. 8), ATP-citrate synthase (ACLY, Supplementary Fig. 9), *H. Sapiens* T-complex chaperonin 16-mer (TRiC, Supplementary Fig. 12) and inositol 1,4,5-trisphosphate receptor type 3 (ITPR3, Supplementary Fig. 12). Despite no information on the selected experimental intermediates is fed to the algorithm, eBDIMS2 always tends to closely approach the cryo-EM intermediates before converging to the target with RMSDs as low as ~3-4 Å (Supplementary Fig. 14), as already found in our previous work on smaller proteins[1].

Comparison with other path-sampling algorithms: computing times, target convergence, and sampling in the experimental PC space

We compared eBDIMS2 transition pathways with those from our previous eBDIMS code[1,7] and nine other algorithms that have been widely used for modeling conformational transitions[11] and whose executables were available, i.e., iMOD[12], GOdMD[13], NGENI[14], ICONGENI[15], Climber[10], NOLB[16], ENI[17], aANM[18], ANMPathway[19]. All methods were used with their default parameters on the Linux workstation mentioned above.

These path-sampling algorithms exhibit both differences and similarities. First, they can adopt different representations of the protein DOFs. While iMOD and ICONGENI use soft torsional angles of the backbone, NOLB uses the DOFs of local rigid blocks extrapolated in a non-linear fashion, and all other methods use Cartesian coordinates, often considering only positions of C^{α} atoms. Second, they can adopt different frameworks to simulate the protein dynamics. eBDIMS and eBDIMS2 use Brownian Dynamics (BD), while GOdMD exploits discrete Molecular Dynamics (dMD) simulations. iMOD and ICONGENI use internal-coordinate Normal Mode Analysis (NMA), NGENI and aANM use Cartesian-coordinate NMA, and NOLB uses rigid-block non-linear NMA. A different scheme is used by Climber, ENI, and ANMPathway, which are based on stepwise refinement iterations on morphing conformations between the two end states. Third, these methods differ for the criteria used to bias the transitions to the target conformation R_t. eBDIMS uses dynamic importance sampling (DIMS) and minimizes the differences in internal pairwise distances. GOdMD uses a similar scheme but implementing a Maxwell Demon approach with a Monte Carlo energy penalty function. Minimization of RMSD from R_t is used in iMOD and NOLB, while ENI, NGENI, and ICONGENI minimize a cost function based on interatomic distances. Climber adopts a combination of distance-restrained energy

minimization and RMSD biasing. aANM combines small incremental displacements obtained from the low-frequency modes of the end states, while ANMPathway uses interpolations of 3D coordinates followed by energy minimizations to relax intermediate conformers. Lastly, these algorithms can generate either reversible or irreversible pathways. If the pathway is reversible, i.e., if the transition $R_0 \rightarrow R_t$ is equal to the transition $R_t \rightarrow R_0$, the algorithm is classified as linear. On the other hand, non-linear methods usually generate irreversible pathways, i.e., the forward ($R_0 \rightarrow R_t$) and backward ($R_t \rightarrow R_0$) transitions are different. eBDIMS, eBDIMS2, iMOD, GOdMD, NGENI, ICONGENI, Climber and NOLB are non-linear methods, while ENI, aANM, and ANMPathway are linear.

In this work, we used four proteins of increasing size to compare the algorithm performances, i.e., RBP (271 residues), RNaseIII (432), SERCA (993), and GroEL 7-mer (3,626). We chose these systems as they do not exhibit missing residues in the polypeptide chain, which is a necessary condition for the applicability of most path-sampling algorithms. Supplementary Fig. 15 shows the PCA spaces of these protein ensembles, together with the projections of all transition pathways, whereas Supplementary Fig. 16 reports the computing times to simulate these transitions.

For RBP, the first two PCs cover almost the entire variance in the ensemble, with PC1 (~98%) representing the opening-closing mechanism, and PC2 (~2%) showing rotations in the two domains (Supplementary Fig. 15a). PC1 broadly separates the RBP ensemble in three clusters of varying opening angles: unbound/open conformers (e.g., 1ba2), intermediate states (e.g. 1urp and 2gx6), and ligand-bound/closed conformations (e.g., 2dri)[1]. All methods are able to model the transition between the open and closed conformation, and all non-linear algorithms can reach a convergence with RMSD < 2 Å (Supplementary Fig. 16a). eBDIMS2, eBDIMS, iMOD, Climber, and GOdMD also get close to the experimental intermediates, whereas the pathways from other methods (ENI, NGENI, ICONGENI, NOLB, aANM, and ANMPathway) exhibit more straight-like projections in the PCA space. In terms of computing time, Climber is the slowest, taking ~4 and ~7 minutes for the forward and backward transitions (Supplementary Fig. 16a). NOLB, ANMPathway, and ENI are the fastest, taking only 2, 6, and 23 seconds, respectively (Supplementary Fig. 16a-b). All other methods take approximately 1-2 minutes to achieve a reasonable convergence.

For RNaseIII, the first two PCs cover ~95% of the variance, PC1 (~51%) describing the opening or 'breathing' of the two RNA-binding domains, whereas PC2 (~44%) tracks their concerted rotation (Supplementary Fig. 15b). These PCs cluster the ensemble into four functional groups: the closed cluster (e.g., 1yyo), an intermediate state (1yz9), a pre-catalytic state (e.g., 1yyw), and the catalytic cluster (e.g., 2ez6)[1]. The transition pathways involve a complex motion along both PCs. The majority of non-linear methods, such as eBDIMS2, eBDIMS, GOdMD, NGENI, ICONGENI, and Climber, provide pathways with smooth PC projections that are also able to capture an on-path intermediate (1yz9). On the other hand, iMOD irregularly samples a broader space that is not populated by experimental conformers, while NOLB gives rise to cusps in the PC projections (Supplementary Fig. 15b) and cannot reach a convergence lower than 5-6 Å (Supplementary Fig. 16c). Among linear algorithms, ANMPathway cannot describe the full pathway, as the code gets automatically aborted after ~22 hours without achieving convergence. ENI and aANM successfully simulate the full transition, but their pathway shows straight projections between the end states. Most of the methods can sample the RNaseIII transition in approximately 5 minutes or faster (Supplementary Fig. 16c-d), while iMOD and ICONGENI take ~10 minutes and Climber ~15 minutes.

The first two PCs of the SERCA ensemble also cover ~95% of the total variance, with PC1 (~56%) describing the mechanism related to ion pumping and PC2 (~29%) the closure of the actuator (A) and phosphorylation (P) domains. In this case, the 2c9m-1t5s transition follows the PC2 direction, along which three main groups are distributed: the most open E1-2Ca^{2+} structures (e.g., 2c9m), the most closed E1-2Ca^{2+}-P structures (e.g., 1t5s), and an intermediate cluster of E1-Mg^{2+}-bound structures (e.g., 3w5a). PC1 separates an additional cluster of E2-closed conformations[1]. All algorithms are able to simulate the transition of SERCA, and they all tend to sample the same PCA area and capture intermediate conformers. Like in the case of RBP and RNaseIII, all linear algorithms tend to produce pathways with straight projections between the end states (Supplementary Fig. 15c). Compared to the other algorithms, NOLB is the fastest, providing each transition

in only ~15 seconds (Supplementary Fig. 16e). However, its convergence to the target states is poor, with ~3 Å for the 2c9m-1t5s transition, and ~7.5 Å for the reverse pathway (Supplementary Fig. 16e). All other methods require comparable amounts of computing time (Supplementary Fig. 16e-f). eBDIMS, eBDIMS2 and NGENI take ~20 minutes, GOdMD, ENI, and aANM ~30 minutes, ICONGENI and Climber ~40 minutes, with iMOD being the slowest with ~60/70 minutes. These methods exhibit different RMSDs at convergence, e.g., eBDIMS2, eBDIMS, and Climber can reach ~1 Å from the target, while the others stop at ~2 Å (iMOD) or ~3 Å (GOdMD, NGENI, ICONGENI) from the target (Supplementary Fig. 16e-f).

In the three cases shown so far, eBDIMS2 performs well, as it generally requires low computing time with low RMSD convergence (Supplementary Fig. 16), predicts smooth transitions in the PC space, and is able to capture experimental intermediates (Supplementary Fig. 15). However, it is not the top-performing method, and for these medium-size proteins it shows similar efficiency to our previous C^{++} code. However, when we upscale to larger systems (>3k residues), the outcomes are different (Supplementary Figs. 16g-h; see Fig. 2d in the main text). In the case of the larger GroEL 7-mer (~400 kDa), the PC1 eigenvector captures the main opening and closing of the oligomeric ring, separating structures that are trapped in the unbound/closed conformation from those in the GroES-bound/open conformations (Supplementary Fig. 15d). PC2 captures internal rotations in the apical domains that are observed in a set of closed structures more recently observed in time-resolved cryo-EM (e.g., 8bmd). For this system, eBDIMS2 is able to simulate the ~15 Å conformational change of GroEL in less than 1.5 hours, reaching an RMSD from the target of ~0.6 Å (Supplementary Fig. 16g). To do the same, our previous eBDIMS code takes more than 7 hours. It is interesting to note that NOLB was also able to deal with this large system, simulating the transitions in only a fraction of time (~2 minutes, Supplementary Fig. 16g). Yet the RMSD convergence to the target state is of the order of ~10 Å (Supplementary Fig. 16g), implying that the majority of the pathway has yet to be covered (Supplementary Fig. 15d). Incomplete convergence (< 80%) appears to be a consistent characteristic of NOLB pathways, as also shown in Fig. 2c of the original reference[16]. On the other hand, none of the other non-linear/linear methods was able to reach full pathway coverage in less than 12 hours. Climber simply cannot deal with such a large system, while GOdMD runs for ~6 hours and then it stops automatically for poor convergence (~14 Å RMSD from the target). iMOD can approach the targets with RMSDs of ~3-4 Å but it requires ~60 hours of computation. NGENI and ICONGENI require, respectively, ~13 hours and ~15 hours to simulate each pathway direction, achieving final RMSD convergences of ~4-6 Å from the targets (Supplementary Figure 16g). Similarly to RNaseIII, ANMPathway cannot simulate the GroEL pathway, with the code being aborted after ~286 hours, while aANM and ENI complete the simulation in ~16 hours and ~28 hours, respectively (Supplementary Fig. 16h).

Recently, MinActionPath2 was published[20], an extension and improvement of the previous MinActionPath algorithm[21], now also able to deal with large macromolecular assemblies. MinActionPath finds the (common) transition path connecting two protein end states by building the ENM of both conformers, computing the two Hessian matrices, and solving a pair of linear differential equations arising from an overdamped Langevin equation, joined by a non-linear boundary matching condition at the transition point[21]. We did not include MinActionPath and MinActionPath2 in our PC-time comparisons (Supplementary Figs. 15 and 16) because these methods were only available as webservers, which prevented a thorough assessment of computing times, and we could only download transition point PDB files, but not the full transition paths. However, after the recent release of MinActionPath2, we used its webserver to assess the structural quality of the transition intermediates of our four benchmark proteins, i.e., RBP, RNaseIII, SERCA, and GroEL 7-mer, as well as of a few of our larger systems, i.e., the *M. Smegmatis* ATP synthase, the isoform 2 of Nf1, and A2M (see below).

Stereochemistry assessment of the generated intermediates

To quantify structural distortions in the generated intermediates at the CG level, we measured the distances between consecutive C^{α} atoms (i, i + 1). In well-resolved experimental protein structures, these distances are centered at ~3.8 Å with low variation. Supplementary Fig. 17 presents a comparison of the C^{α}-C^{α} distance

distributions for intermediate conformations generated by the various path-sampling methods across our four benchmark proteins: RBP, RNaseIII, SERCA, and the GroEL 7-mer. For reference, the corresponding distributions from experimental end-state structures are also included.

This analysis reveals that some path-sampling methods introduce extreme structural distortions even at the elementary level of CG backbone connectivity. Among them, MinActionPath2 consistently produces the broadest and most highly variable C^{α}-C^{α} distance distributions, with extreme values ranging from as low as ~2.5 Å in RBP, ~0.3 Å in RNaseIII, ~1 Å in SERCA, and ~0.5 Å in the GroEL 7-mer, to as high as ~6 Å, ~17 Å, ~10 Å, and ~10 Å, respectively. These extreme deviations indicate severe disruptions in the local backbone geometry, rendering the resulting conformations unreliable for subsequent atomistic modeling. Other path-sampling methods also tend to yield significant CG distortions.

For RBP, GOdMD generates distances exceeding 5 Å, while NGENI produces values below 3 Å and above 6 Å. NOLB yields distances outside the 3–5 Å range, and aANM introduces deviations below 3 Å and above 8 Å. In RNaseIII, aANM exhibits the most severe deformations, with distances ranging from below 1 Å to above 35 Å. GOdMD produces distances greater than 9 Å, whereas NGENI generates values below 3 Å and above 9 Å. ICONGENI similarly produces values above 9 Å, while NOLB and ENI also lead to extreme distortions, with NOLB yielding C^{α}-C^{α} distances as low as 1 Å and as high as 18 Å, and ENI ranging from below 1 Å to above 5 Å. In the case of SERCA, GOdMD produces neighboring C^{α}-C^{α} distances exceeding 12 Å. NGENI and ICONGENI generate values ranging from below 3 Å to above 9 Å and above 5 Å, respectively. NOLB introduces values below 2 Å and above 13 Å, while both ENI and aANM yields distances below 1 Å, and ANMPathway shows values below 3 Å. For the GroEL 7-mer, GOdMD generates distances exceeding 8 Å, while NGENI produces values below 3 Å and above 5 Å. NOLB and aANM both result in distorted distances ranging from below 3 Å to above 6 Å, and from below 2 Å to above 5 Å, respectively. Taken together, these results highlight the tendency of several path-sampling methods to introduce unrealistic deformations in C^{α}-C^{α} backbone distances, which compromises the structural integrity of the intermediates. Such distortions, particularly when extremely severe like in the case of MinActionPath2, may invalidate the use of these intermediates in downstream atomistic simulations.

On the other hand, iMOD and Climber consistently generate structural intermediates with high CG quality, as evidenced by the narrow distributions of C^{α}-C^{α} distances centered around ~3.8 Å with minimal deviation and similar to those observed in experimental structures (Supplementary Fig. 17). For Climber, this accuracy is attributable to its use of a detailed atomistic molecular-mechanics force-field[10]. In the case of iMOD, the favorable performance likely results from its use of internal coordinates – specifically dihedral angles – which helps mitigate the non-physical distortions commonly associated with Cartesian-based approaches[12].

Supplementary Fig. 17 further reveals that, although eBDIMS2 does not maintain C^{α}-C^{α} backbone distances as precisely as iMOD or Climber, it nonetheless avoids the extreme deviations observed in some of the other methods discussed above. The observed range of C^{α}-C^{α} distances for eBDIMS2 for these four benchmark proteins spans from ~3.3 Å to ~4.3 Å, representing a deviation of ~0.5 Å from the reference values of 3.8 Å. Notably, eBDIMS2 appears to outperform our previous implementation in preserving backbone geometry, particularly in smaller proteins such as RBP and RNaseIII, where the original eBDIMS was found to produce distances below 3 Å. The underlying cause of this marginal improvement is not fully understood, but it may stem from the lower cutoff for non-bonded interactions (8 Å) that we employed in eBDIMS2. This smaller cutoff results in a lower number of anisotropic forces acting on individual C^{α} particles in the BD simulation, potentially helping in reducing anisotropic C^{α}-C^{α} fluctuations.

We also assessed the CG quality of all eBDIMS2-generated intermediates for the large-protein ensemble dataset. Supplementary Fig. 18 shows the distribution of distances between consecutive C^{α} atoms for all 191 eBDIMS2 mid-point intermediates (panel a), as well as for all 124 experimental end-state conformers (panel b) and all 872 experimental structures used in our ensembles (panel c). Boxplot representations of these distributions are reported in panel (d). The average distance values in all eBDIMS2 intermediates are centered at ~3.8 Å, with 25th and 75th percentiles at ~3.7 Å and ~3.9 Å, and minimum and maximum values (not including outliers) at ~3.4 Å and ~4.2 Å (Supplementary Fig. 18a). Minimum and maximum values, also

including outliers (red points in panel d), are 2.4-6.2 Å for eBDIMS2, 2.7-6.2 Å for end-state conformations, and 2.6-6.2 Å for all experimental structures. These results show that, as already observed above for RBP, RNaseIII, SERCA and GroEL 7-mer, eBDIMS2 tends to add only minor (sub-Å) stereochemical distortions to the C^{α} backbone and does not add extra outliers to those already present in experimental models.

Then, we focused our attention onto three larger proteins exhibiting large-scale and complex structural transitions: *M. Smegmatis* ATP synthase, Nf1 isoform 2, and A2M. We compared the feasibility of pathway generation and the quality of the intermediate states by eBDIMS2 to those obtained by MinActionPath2 and NOLB – which are currently the only path-sampling approaches capable of handling macromolecules of this size. For all three cases, MinActionpath2 (webserver) and NOLB are extremely fast, providing final output files in less than 30 minutes. Yet they tend to produce higher structural distortions compared to eBDIMS2.

For ATP synthase, we simulated the 14 Å conformational transition from rotary state 1 (PDB: 7jg5) to state 2 (7jg6), characterized by a rigid 120° rotation of the F_0 rotor *c*-subunits (Supplementary Fig. 5) coupled with concerted opening and closing motions of the *β*-subunits (Fig. 4 in the main text). Only eBDIMS2 is able to correctly reproduce this motion, achieving a convergence of ~1.2 Å to the target in ~9 hours of simulation (Supplementary Table 7). While much faster than eBDIMS2, neither MinActionPath2 nor NOLB can capture the full transition path (Supplementary Fig. 19). MinActionPath2 fails to simulate the rotation of the F_0 domain, which remains locked in the configuration of state 1 (Supplementary Fig. 19a). NOLB shows partial successes in modeling some degree of the F_0 rotation, but it fails to converge to the target state (~12 Å from the target, Supplementary Fig. 19b). Additionally, the analysis of C^{α}-C^{α} distances reveals significant backbone distortions in the MinActionPath2 intermediate: while the experimental end-state structures exhibit C^{α}-C^{α} distances ranging from ~2.9 to ~3.9 Å, the MinActionPath2 transition point displays values spanning from ~0.6 to ~9.3 Å (Fig. 4a in the main text), suggesting major structural artifacts. In contrast, the eBDIMS2 intermediate and the final NOLB frame maintain C^{α}-C^{α} distances within more acceptable ranges, i.e., 2.8-4.2 Å and 2.9-4.6 Å, respectively, better aligning with those observed in the experimental conformations.

For Nf1, the 23 Å transition from the active (7pgt) to the inactive state (7pgr) of isoform 2 involves a complex and large-scale roto-translation of the GRD and Sec14-PH domains[22]. To simulate this transition, eBDIMS2 takes ~5 hours, achieving a remarkable convergence to the target of ~0.9 Å (Supplementary Table 7). In this case, the MinActionPath2 transition point is even more distorted than in the previous case (Supplementary Fig. 20a). The GRD domain is completely disrupted, and the distribution of C^{α}-C^{α} distances range from ~0.3 Å to an astonishing maximum of ~33.9 Å (Fig. 4b in the main text), being completely out of scale compared to the values in experimental end states (3.8-3.9 Å) and the eBDIMS2 intermediate (3.4-4.2 Å). Also in this case, NOLB fails to accurately model this transition, with the final frame remaining at ~12 Å away from the target structure (Supplementary Fig. 20b). In this case, NOLB also introduces substantial distortions in the CG backbone, as evidenced by C^{α}-C^{α} distances ranging from ~2.3 Å to as much as ~18 Å, far beyond the range observed in the experimental structures and the eBDIMS2 intermediate (Fig. 4b in the main text). Finally, for the 29 Å transition of A2M from its native (7o7l) to its activated state (7o7p), MinActionPath2 provides a transition point where the moving domains are extremely distorted (Supplementary Fig. 21a), with C^{α}-C^{α} distances ranging from 0.7 Å to 19.4 Å (Fig. 4c in the main text). eBDIMS2 can simulate this large-scale transition in ~5.5 hours reaching a convergence of ~1.5 Å from the target (Supplementary Table 7), while NOLB cannot converge (~10 Å from the target, Supplementary Fig. 21b) and also generates high distortions in the C^{α}-C^{α} connections, with distances from ~2.5 Å to ~7.4 Å. In this case, distances in the experimental structures and the eBDIMS2 intermediate are in the range 3.7-4.6 Å and 3.4-4.8 Å, respectively (Fig. 4c in the main text).

Overall, these comparisons indicate that NOLB generally fails to achieve satisfactory convergence to the final states (see also Supplementary Fig. 16) and both NOLB and MinActionPath2 tend to introduce severe distortions in the CG backbone. In contrast, although substantially slower, eBDIMS2 stands out as the only method among the three capable of reliably simulating these complex and large-scale motions with a high degree of convergence. The intermediate states generated by eBDIMS2 – while far from perfect – exhibit CG structural features that are broadly consistent with typical distributions observed in experimental data. These

differences among the three models likely stem from the underlying methodologies. For instance, eBDIMS2 leverages a BD-based molecular simulation framework, whereas NOLB relies on rigid-block normal mode analysis (NMA). The latter, while more efficient, often fails to provide suitable reaction coordinates for tracing complex conformational transitions and can easily lead to unphysical distortions in the resulting structures.

Finally, we assessed the atomistic quality of the generated intermediates following all-atom reconstruction using the recent deep learning-based method cg2all[23] and quality assessment via MolProbity[24]. Supplementary Tables 9-12 summarize key structural metrics – Ramachandran favored/outlier regions, rotamer outliers, clash scores, bond lengths and angles, and overall MolProbity scores – for the generated intermediates of RBP, RNaseIII, SERCA, and the GroEL 7-mer. These results largely mirror the CG-level trends already observed in Supplementary Fig. 17. Climber and iMOD consistently produce the most accurate and high-quality intermediates, while the remaining path-sampling methods frequently introduce significant structural distortions upon atomistic reconstruction. eBDIMS and eBDIMS2 are also found to perform quite poorly immediately after reconstruction with cg2all, as indicated by uniformly high MolProbity scores (>3), largely due to a high incidence of atomic clashes (Supplementary Tables 9-12). However, we found that these issues could be fully resolved through short molecular refinements, which adjust the side-chain placements while preserving the backbone conformation of the intermediate (C^{α}-RMSD < 1Å). Supplementary Tables 13-16 report the improvements in MolProbity metrics for eBDIMS2 intermediates after successive rounds of energy minimization (5,000 steps), equilibration (125 ps under NPT conditions), and short (1 ns) MD production runs. In all cases, atomic clashes are already eliminated during the minimization step, and overall MolProbity scores progressively improve, ultimately reaching values comparable to those of experimental structures (<2). As an illustrative example, Supplementary Fig. 22 presents the progressive improvement of the Ramachandran plots for the GroEL 7-mer intermediate conformer throughout the refinement procedure. These findings demonstrate that, while initially suboptimal, eBDIMS2 intermediates are realistic enough since they can attain high atomistic quality following minimal refinement. This makes them suitable for downstream MD simulations and detailed atomistic analyses[25].

Molecular Dynamics (MD) simulations

To assess the sampling of eBDIMS2 pathways, we performed MD simulations of several systems (Supplementary Table 17). We carried out Targeted MD (TMD)[26] to compare eBDIMS2 transitions with atomistic pathways, and unbiased MD simulations (starting from end-state and eBDIMS2 intermediate conformations) to obtain Free-Energy Landscapes (FELs). Additional details on MD protocols and the investigated systems can be found in the Methods Section of the main text and in Supplementary Table 17, respectively.

Supplementary Fig. 23 shows the results of TMD simulations for RBP (271 residues), RNaseIII (432), SERCA (993), DNA-PKcs (4,118), and ACLY (4,384), respectively. For RBP, RNaseIII and SERCA, 1 ns was enough to achieve convergence, while 2-ns production runs were used for DNA-PKcs and ACLY. For these two larger proteins, we also used the SWISS-MODEL[27] webserver to reconstruct missing gaps in the sequence before carrying out atomistic simulations. By comparing projections on experimental PC spaces, we observe that TMD is in general agreement with eBDIMS2, as both methods provide transition pathways that sample similar portions of the conformational space. In some cases, the level of agreement between TMD and eBDIMS2 paths is impressive, e.g., the opening transition of RNaseIII and the activation-inactivation pathways of DNA-PKcs (Supplementary Fig. 23, see Fig. 5 in the main text). The inactivation pathway of DNA-PKcs (7k0y-7k19) samples the same portion of the conformational space in all TMD replicas. On the contrary, the activation transition (7k19-7k07) goes straight from the inactive (7k19) to the active (7k0y) state in replica 2, while it visits the intermediate (DNA-bound inactive) conformation (7k1n) in replicas 1 and 3 (Supplementary Fig. 23, panel 4). eBDIMS2 is also able to sample this two-step process if the trajectories from the inactive to the intermediate state (7k19-7k1n), and from the intermediate to the active state (7k1n-7k0y) are merged together.

While the sampling between eBDIMS2 and TMD is in fairly good agreement, the former requires much less computational resources. As an example, the 7k19-7k07 activation pathway of DNA-PKcs was computed by eBDIMS2 in only ~29 minutes (Supplementary Table 7) using 16 OpenMP threads on a standard desk computer, while it took ~11 hours and 128 parallel cores on our high-performance computing servers for TMD. This suggests a total CPU consumption of ~8 and ~1,400 core-hours for eBDIMS2 and TMD, respectively. Moreover, TMD (like all MD simulations) requires a careful preparation of the molecular system, the creation of a solvated water box, a few rounds of energy minimizations and equilibrations, and generally leads to the prediction of different trajectories when different starting seeds are used. On the contrary, eBDIMS2 simulations does not need particular preparations of the system (other than ensuring proper correspondence between reference and target structures), are computationally much cheaper and mostly deterministic.

Results from unbiased MD simulations are reported in Supplementary Figs. 24-27. Three independent 200-ns replicas were carried out for different conformational states of the same five proteins (Supplementary Table 17), and they were merged together to generate FELs. PCA of each MD trajectory, i.e., essential dynamics (ED)[28], was also performed to extract the motions that can describe most of the variance in the MD simulation. The similarity between ED and PC modes was assessed by computing the overlap $O_{i,j}$:

$$O_{ij} = \frac{\left|\mathbf{ED_i}^{T} \cdot \mathbf{PC_j}^{T}\right|}{\sqrt{\mathbf{ED_i}^{T} \cdot \mathbf{ED_i}^{T}}\sqrt{\mathbf{PC_j}^{T} \cdot \mathbf{PC_j}^{T}}} \qquad \text{(S2)}$$

where $\mathbf{ED_i}$ is eigenvector corresponding to the ith ED mode, and $\mathbf{PC_j}$ is eigenvector of the jth experimental PC. $O_{i,j}$ values range from 0 to 1, 0 meaning no correlation (orthogonality) between the two eigenvectors and 1 implying perfect overlap. We also used the Root Mean Squared Inner Product (RMSIP) to assess the similarity between the two vectorial spaces:

$$RMSIP_m = \sqrt{\frac{1}{m}\sum_{i=1}^{m}\sum_{j=1}^{m} O_{ij}^{2}} \qquad \text{(S3)}$$

where m is the total number of ED and PC eigenvectors considered (we used 3, 5, and 10). Supplementary Table 18 reports the results of ED analysis, showing the amount of variance captured by the first two ED modes, RMSIP values, and maximum overlap scores.

For RBP, the correlation between MD trajectories and the experimental PCs is impressive, with RMSIPs up to ~0.8 and individual mode overlaps up to 0.97 (Supplementary Table 18). As we have shown previously[29], eBDIMS paths of RBP are in agreement with data from atomistic MD that capture the opening-closing motion. We also observe a large overlap between the distinct FELs that are obtained from the open (1ba2) and closed (2dri) end states, as well as from the atomistic reconstructions of the eBDIMS2 mid-point intermediates along the closing (1ba2-2dri) and opening (2dri-1ba2) transitions (Supplementary Figs. 24 and 25). This confirms that eBDIMS2-generated conformers can be used for subsequent atomistic analysis and are able to sample conformations that are consistent with those obtained from the experimental end states. In RNaseIII, MD simulations from the open conformation (1yyw) do not sample broadly the conformational space, while in one replica from the closed conformation (1yyo) the eBDIMS2 opening pathway is reproduced with tremendous accuracy by the unbiased MD trajectory (Supplementary Fig. 24, replica 1 from 1yyo). Unbiased simulations from the eBDIMS2-generated intermediate along the opening pathway (1yyo-1yyw) allow to enhance the sampling of the conformational space and visit additional RNaseIII atomistic conformations (Supplementary Figs. 24-25), although 200 ns might not be enough for exhaustive sampling of all possible conformations from the intermediate. For SERCA, MD trajectories from the closed state (1t5s) are not able to escape the low-energy minimum, while those from the open structure (2c9m) sample the direction of the transition along PC2 (Supplementary Fig. 24). In this case, overlaps between ED modes and PC eigenvectors reach values as high

as 0.83 and RMSIP scores of ~0.7. Like in the case of RNaseIII, MD simulations from an eBDIMS2 intermediate allow to enhance the sampling of the conformational space (Supplementary Figs. 24-25).

In DNA-PKcs, the unbiased simulations from the active state (7k0y, Supplementary Fig. 26) do not exhibit significant structural variations, as revealed by the limited sampling in the PC space (Supplementary Fig. 27) and by low similarities between ED and PC modes (< 0.4, Supplementary Table 18). Although overlaps do not reach more than ~0.6 even in the simulations from the inactive state (7k19), MD trajectories starting from this more flexible conformation display a somewhat larger degree of conformational dynamics, especially along PC1, which points in the direction of the inactivation-activation pathways (Supplementary Fig. 26). The conformational variability in the MD simulations of ACLY (Supplementary Figs. 26-27) is much larger. When simulations are started from the apo-open state (6pof), the three replicas exhibit large motions along PC1, as also reflected from the large overlaps between ED eigenvectors and PC1 (~0.8, Supplementary Table 18). The FEL generated from these trajectories samples a large portion of the conformational space along PC1 but limited only to high PC2 values (Supplementary Fig. 27). This is likely due to the fact that conformations lying at low-PC2 values are only stable in presence of ligands (citrate, coenzyme-A, etc.). The eBDIMS2 pathways between an inhibited ACLY state (6o0h) and a CoA-bound conformation in a partially open state (6uia) are consistent with the directions of motion sampled by these MD simulations. On the other hand, MD trajectories starting from the holo-closed state of ACLY (6hxh, in absence of ligands) describe opening motions towards higher values of PC2, spontaneously going back to the apo-open state (6pof), in agreement with the eBDIMS2 pathway from the holo-closed to the apo-open conformation (Supplementary Fig. 26). In this case, we observe that the FEL obtained from the holo-closed state in absence of ligand clearly captures intermediate experimental conformations, e.g., 8g1e, 6ui9, etc., which are also approached by the eBDIMS2 transition pathway (Supplementary Fig. 27).

In this work we also used simulations of the *SARS-CoV-2* spike glycoprotein, publicly available from the Amaro's lab[30,31]. Several unbiased MD simulations from the open (wild-type and double-mutant) and closed conformation (Supplementary Table 17) of the spike glycoprotein[30] were downloaded from https://amarolab.ucsd.edu/covid19.php, and projected on the experimental PC space of the *SARS-CoV-2* spike (Supplementary Fig. 8, panel 3). The resulting FELs are shown in Supplementary Fig. 28. As can be seen, simulations starting from the open state with one receptor-binding domain (RBD) up (Supplementary Figs. 28a-d) tend to sample the area towards the fully closed state of the trimer (cluster containing our reference closed conformation, 6xr8). This is especially evident for simulations with the N165A-N234A double-mutant (Supplementary Figs. 28c-d; see Fig. 6b in the main text), whose FEL is pointing in the same directions of the eBDIMS2 transition paths between the fully closed and one-RBD-up conformation. Conversely, all MD simulations starting from the closed state do not show a substantial amount of sampling (Supplementary Fig. 28e), suggesting that this conformation lies in a low-energy minimum. The results from the ED calculations show relatively low similarity between ED eigenvectors and experimental PCs (Supplementary Table 18). This is due to the fact that the experimental PCs capture large-scale conformational changes of the RBDs and underlie a large variety of conformational diversity, i.e., fully closed (6xr8) and fully open (8cy6) trimers, as well as conformations with only one (7a94) or two (7tpr) RBDs up. Such large-scale motions cannot be easily captured by unbiased MD simulations.

We also made use of another simulation from the Amaro's lab[31], that employs a Weighted Ensemble (WE) enhanced sampling approach[32] to capture the transition from the fully closed state of the spike to the conformation with one RBD up. This transition is necessary for spike activation, but it can occur on biological timescales that are generally not accessible by traditional unbiased MD, as shown above. In the WE method, several short simulations are run in parallel along selected reaction coordinates and the trajectories that rarely sample high-energy regions are replicated, while those that sample low-energy ones are merged. This makes sampling rare events computationally tractable[31]. Supplementary Fig. 29 shows the projection of the 175-ns WE simulation of RBD opening on our experimental PC space. Towards the end of simulation (~150 ns), the trajectory is clearly moving away from the cluster of closed conformations and samples the cluster of one-RBD-up states. The initial WE simulation was run for 8.77 days on 80 GPUs collecting a total sampling of

~7.5 μs, while the total analysis lasted 26.74 days on 100 GPUs, amount to a total sampling of ~70 μs[31]. eBDIMS2 can provide a similar description for the RBD opening, simulating the transition pathway from a fully closed trimer (6xr8) to the open one (7a94), in just ~1.2 hours (Supplementary Table 7) on a standard desktop computer, using 16 OpenMP threads (which amounts to only ~20 CPU core-hours), and achieving a convergence to the target of ~0.6 Å. It is important to note that WE simulations does typically aim to quickly capture one transition pathway, but rather they generate multiple trajectories, that are subsequently weighted and replicated or pruned to accelerate the discovery of rare events such as transitions. This makes a direct comparison of WE and eBDIMS2 simulation times not entirely appropriate. Nonetheless, such a general comparison in terms of computing time and resources provides a useful indication of the substantial difference in computational cost between a CG path-sampling method like eBDIMS2, and an enhanced sampling approach based on atomistic MD, when applied to the same macromolecular system.

The WE simulation and the eBDIMS2 pathways for the spike glycoprotein have different starting and end points (Supplementary Fig. 29a). To assess the similarity between the two trajectories, we computed the RMSD between each WE and eBDIMS2 frame describing the RBD opening. Supplementary Fig. 29b reports RMSD values between WE and eBDIMS2 trajectories, showing that intermediates along the two pathways have RMSD values as low as ~4 Å, which is similar to the value between the two different starting states. Supplementary Fig. 29c highlights the pairs of WE and eBDIMS2 intermediates with RMSD close to the minimum value (lower than 2% difference). This figure shows that the eBDIMS2 sequential transition generally agrees with the opening motion captured at the end of the WE simulation (Supplementary Fig. 29d), indicating that eBDIMS2 is able to provide a description of the RBD opening consistent with the more expensive WE method.

Comparison with recent DL methods: BioEmu and AF_unmasked

Since the release of AlphaFold (AF)[33], deep learning (DL) approaches have been increasingly exploited to generate conformational ensembles of proteins. Among the most recent examples are BioEmu[34] and AF_unmasked[35]. BioEmu, trained on static structures, >200 ms of MD trajectories, and experimental stability data, has been designed to sample thousands of statistically independent conformations that approximate the equilibrium distribution of a protein. In contrast, AF_unmasked builds directly on AF's template-handling strategy and has been explicitly developed to predict diverse conformations of large, multimeric, and flexible protein assemblies.

BioEmu is currently restricted to monomeric proteins. In our dataset of large systems, this limited its applicability to DNA-PKcs, the only monomeric example. Unfortunately, the model was unable to generate conformations for DNA-PKcs, most likely due to its large size (~450 kDa full sequence), which far exceeds the protein sizes (< 90 kDa) explored in the domain-motion benchmark of the model[36]. On the other hand, the method could successfully be applied to RBP and SERCA (Supplementary Fig. 30). After filtering out unphysical conformations, 971 models were generated for RBP in ~4.5 hours and 889 for SERCA in ~20.5 hours using the same Linux workstation described above.

For RBP, BioEmu is found to accurately capture known experimental states (Supplementary Fig. 30a), with a clear bias towards the closed conformation (2dri, $RMSD_{min}$ ~0.6 Å), followed by the intermediate (1urp, $RMSD_{min}$ ~1.2 Å) and open form (1ba2, $RMSD_{min}$ ~1.9 Å). This bias toward the closed state is also evident from the projection of all conformers in the experimental PC space (Supplementary Fig. 30b). The analysis of the gyration radii (R_g) of the obtained conformers (Supplementary Fig. 30c) highlights that, in addition to reproducing the experimentally observed ensemble and preferring more compact states, the model also tends to sample exaggeratedly expanded structures characterized by partially unfolded domains. For SERCA, BioEmu has a greater difficulty in reproducing the full range of experimental conformers. The generated ensemble is again found to display a strong bias toward the closed E2 state (e.g., 2zbe), which is captured with a minimum RMSD of ~2.8 Å. By contrast, the open E1-2Ca^{2+} state (2c9m) and the closed E1-2Ca^{2+}-P state (1t5s) are barely captured, with minimum RMSDs exceeding ~7.5 Å and ~5.4 Å, respectively (Supplementary

Figs. 30d-f). As with RBP, the model generates conformations featuring partially unfolded domains (Supplementary Fig. 30e), which do not correspond to any experimental evidence currently available in the PDB or MD data (Supplementary Figures 24-25).

Differently from BioEmu, AF_unmasked can be applied to larger multimeric systems. In the original publication[35], the method was also tested on isoform 2 of Nf1, which is included in our large-protein dataset. Fifteen AF_unmasked models (three predictions from five relaxed models) were retrieved from the figshare AF_unmasked repository[37], and were found to exhibit substantial conformational variability, particularly in the GRD and Sec14-PH domains and in the curvature of the Nf1 scaffold[35]. Here, we report comparison of these AF_unmasked predictions to the experimental conformations and intermediates generated by eBDIMS2 (Supplementary Fig. 31). To better assess conformational differences, we projected all experimental, eBDIMS2, and AF_unmasked conformers onto a 2D space defined by the first principal component (PC1) of the Nf1 experimental ensemble, which clearly captures the GRD–Sec14-PH domain motion, and the lowest-frequency normal mode (NM1) from the edENM[6] built on the inactive state, which is associated with scaffold bending (Supplementary Fig. 31a). Consistent with the authors' findings[35], some AF_unmasked models capture the inactive conformation (7pgr), but none is able to approach the active state (7pgt). Most AF_unmasked conformations populate regions of the PC1–NM1 space characterized by exaggerated scaffold curvature (high NM1 scores), distinct from both experimental structures and eBDIMS2 intermediates. To further highlight this conformational diversity, we also defined three angular metrics: χ, which quantifies the scaffold bending (average angle between residues V289, N1963, and E2693 across the two chains *F* and *N*), and θ_F/θ_N, describing GRD–Sec14-PH orientation in each chain (angles between V1291, N1963, and T1646; Supplementary Fig. 31b). Lower χ values correspond to more bent-curved scaffolds, while lower θ values to more closed/inactive arrangements of GRD–Sec14-PHs in the two chains. In the (χ, θ_F, θ_N) 3D space, AF_unmasked conformers are found to display an even broader and more scattered variation, illustrating better the inability to model conformers close to the experimentally known active state (7pgt, quasi-straight scaffold with only one GRD–Sec14-PH in the open conformation) or in the conformational space in between the two experimental structures. By contrast, eBDIMS2 intermediates sample a smooth continuum between the inactive and active conformations across both global (PC1–NM1) and local (χ, θ_F, θ_N) descriptors.

Taken together, the comparisons with BioEmu and AF_unmasked highlight a key distinction between path-sampling approaches such as eBDIMS2 and DL methods. eBDIMS2 is designed to bridge the conformational space between two (experimentally determined) end states, making it well suited for exploring directional transition pathways and generating mechanistically coherent on-path intermediates. In contrast, DL approaches like BioEmu and AF_unmasked enable a much broader sampling of conformational space which, besides recovering known experimental states, could include unfolded conformations unsupported by both experimental data and MD simulations. At the same time, the conformational biases inherited from training data itself, e.g. overrepresentation of closed structures, reduced flexibility in MD from closed states, etc., may also make the DL methods to miss biologically relevant intermediate conformers, leaving gaps between end states.

Supplementary Table 1. Three medium-size proteins (< 1k residues) used as benchmark to assess eBDIMS2 optimal parameters, comparison with other path-sampling algorithms and all-atom MD simulations. Oligomeric state refers to the most relevant oligomerization for the protein function. When this is uncertain or a protein has been captured in several oligomeric forms, all relevant states are considered. Number of common residues is the total number of amino acids considering missing residues in the structural ensemble. Mass provides an estimate of the molecular weight of the protein, based on the number of common residues and considering an average mass of 110 Da per amino acid. Experimental structures used for PCA lists all the PDB models used for the generation of the structural ensemble. When present, suffixes "_1", "_2", "_3", etc., indicate the first, second, third, etc., molecular entity in the PDB file. Structures used for the eBDIMS2 transitions are shown in *italic*, while the reference used for PCA is shown in ***bold italic***.

**Protein**	**UniProt ID**	**Oligomeric state**	**Number of common residues**	**Mass (kDa)**	**Experimental structures used for PCA**
Escherichia Coli ribose import binding protein (*RBP*)	P02925	Monomer	271	30	***1ba2_1***, *2dri*, 1ba2_1, 1dbp, 1drj, 1drk, 1urp_1, 1urp_2, 1urp_3, 1urp_4, 2gx6
Aquifex Aeolicus ribonuclease III (*RNaseIII*)	O67082	Homodimer	432	48	***1yyo***, *1yyw_1*, 1yyk, 1yyw_2, 1yz9, 2ez6, 2nue, 2nuf, 2nug, 4m2z, 4m30
Oryctolagus Cuniculus Sarcoplasmic/endoplasmic reticulum Ca^{2+} ATPase 1 (*SERCA*)	P04191	Monomer	993	109	***2c9m_1***, *1t5s*, 2c9m_2, 5xab, 5xa7, 5xa8, 3ar7, 3n5k_1, 3n5k_2, 5zmw, 5xa9, 6rb2, 2zbd, 3ar5, 3w5d, 3ar4, 4uu0, 5xaa, 4ycl, 5zmv, 3ar6, 5a3s_1, 5a3s_2, 4xou, 1wpg_1, 1wpg_2, 1wpg_3, 1wpg_4, 3ar3, 6hef, 2zbf, 4bew_1, 4bew_2, 1su4, 2agv_1, 2agv_2, 3fgo_1, 3fgo_2, 3ar2, 3w5c, 3ar9, 4uu1, 2zbg, 2dqs, 3ar8, 3n8g, 3b9b, 3gbp, 6yaa, 3w5a_1, 3w5a_2,1t5t, 2c8k, 2eau, 2eat, 2c8l, 3b9r_1, 3b9r_2, 1vfp_1, 1vfp_2, 1xp5, 2yfy, 3fps, 3j7t, 4j2t, 3w5b, 2c88, 2ear, 2by4, 1iwo_1, 1iwo_2, 2zbe_1, 2zbe_2, 1kju, 5a3r, 4ycm, 4ycn, 3ba6

Supplementary Table 2. List of the 511 UniProt entries retrieved after the first stage of the bioinformatic search. These are all UniProt IDs that satisfy the following criteria: (i) reviewed entries; (ii) monomeric sequence of > 500 amino acids; (iii) at least 3 cryo-EM models available with medium-to-good resolution (< 5 Å), with sufficiently large molecular weight (400 kDa – 3 MDa), and sufficiently large number of modelled residues (3,000 – 30,000). Search performed in January 2024.

**UniProt entries**
P0DTD1, E9Q555, P11716, Q92736, E9Q401, Q9NR09, Q14204, P78527, P36022, P38811, Q03131, Q8N2C7, Q03133, P03186, Q13315, P21359, P04275, O95071, Q9UQ35, P29994, Q14573, Q9P2D8, A6H8Y1, P42345, E2JF22, Q8JUX6, P33334, P38111, Q6P2Q9, P21951, Q8N201, P32639, O75643, P07149, P34731, P10587, P11414, P24928, Q9H1A4, P21675, P19097, P43098, P16157, Q923J1, Q14997, Q07163, Q8IZF0, P04050, Q7KZ85, O95602, Q6R327, P15398, P01031, P10964, P51532, Q8NI27, Q04781, Q8RQE8, Q05755, Q9Y4B6, P35956, O94759, Q03468, P53115, O60306, Q00960, P01023, A0A0R4IMY7, Q5SXM2, P09884, Q96RY7, P04051, O15360, O60244, Q9BXW9, P0A8T7, Q9HWC9, P9WNA5, O14802, Q14152, P03226, P40340, Q2HRA7, Q9ULK4, Q04693, Q51561, K9N5Q8, P36334, P0A8V2, Q8NEZ3, Q8N122, Q6TS43, P03726, A0QS66, P9WGY7, O75533, Q8CJT1, Q5JTH9, Q04183, Q48245, P0DTC2, P59594, P05674, O14727, Q5XXP3, Q92833, Q9HBG6, P53840, Q12791, P08518, Q14562, Q6QGE7, Q15393, Q2NKJ3, Q8TAQ2, Q8TD43, Q7TN37, Q9H0H0, P22138, Q03720, P37871, Q6P1X5, P37870, O14114, Q08965, Q9P2L0, P11498, P9WGY9, P30876, Q9P7X8, Q6PD62, Q9LK40, P60281, Q9L0L0, P22276, P30958, P24384, Q16531, Q9H9Y6, Q9NW08, P11513, Q8RQE9, Q8R4D5, Q03660, P53396, O00267, O00268, P19263, P17255, O60566, P52002, O60231, Q12532, Q96P20, P00722, Q6P9B9, P53091, P36048, Q08162, Q75QN2, P38764, O75448, Q06337, Q15029, P24279, P49955, P60240, P38249, Q96HW7, Q9NVH2, P36776, Q99460, P04133, Q6QGE9, P32565, O94906, P17427, P35439, P13671, P30665, Q5VWG9, Q01454, Q99613, P02730, P49917, P33602, P42260, Q13200, Q9HCG8, P49736, Q8IYB3, P19735, Q55544, Q13435, P32786, P0A705, P49735, Q9UL03, P19491, Q0VG06, P11512, P76272, Q9Y2X0, Q9HBA0, P29469, Q26454, P33991, Q04048, Q8NB91, P63284, Q9HCS7, P9WPC9, P9WPD1, Q9BZJ0, P38132, P10643, Q00578, P25582, P32563, Q9HV55, P25286, Q29466, Q93050, P25694, P43572, P30260, Q9UJX6, Q14566, Q9XYU1, Q9V461, P55884, P32497, Q80WG5, Q8IWT6, P15917, P25205, Q9UJX5, Q04660, P55072, Q8R0I0, Q9BYF1, Q2NL82, Q8R502, O42832, Q99459, O43290, P40136, Q15542, P03747, Q15459, Q07381, Q96RN5, Q56223, P19447, P32794, P06839, P29496, O32215, Q9IMP3, P13423, P06103, P02786, P18074, P0ABH9, P0A405, P53946, Q9UJX4, Q9IMP4, P58576, P12154, P29254, P56766, Q8MFA3, Q9FGI6, P03725, Q04377, Q15910, P0AG20, P18708, P0A407, P58565, O74399, Q15022, P09144, P41895, P33992, P56767, Q8MFA2, Q12149, Q9VGW6, P13010, P29255, Q13823, Q94511, P15690, P28331, P46199, Q91VD9, Q6SZW1, Q13144, Q9XYU0, P33993, Q2M385, P0A9H3, A1L314, P32501, Q92541, Q2YD98, Q9IMP5, Q9NVU0, P0A6M8, P32325, O15234, P02787, P16452, Q5SHN5, P24482, Q96EY7, P32569, Q12309, Q9UXG1, P0CG48, Q14C51, O43395, Q13769, P77455, P49848, Q8TAF3, P29388, Q8WVC0, Q07896, Q9NV88, Q96FV9, Q9B6D3, P32349, P12754, Q9NVC6, Q08032, Q02892, O94659, P32776, P53145, Q13409, Q9BZE4, P39682, P36056, P36070, Q9SM09, O15287, P53207, Q13042, Q9BRD0, P40991, P31404, P38606, Q29048, P00579, P33607, Q12136, P12956, P9WQN5, O60164, P03921, O78756, P03920, P25808, Q56227, Q9TDR1, P53261, P03915, Q5TA45, Q14181, Q9UJX2, P29056, P33599, P46678, P47083, Q03862, P07358, Q03654, Q8IXH7, P30153, O00541, Q12420, O95391, O53945, P07357, Q79VD7, Q9GS23, Q8N1G4, P33755, Q8WX92, O60508, P09032, Q09916, Q56403, A0JNT9, P53333, P03420, P10809, P12538, O34693, P03233, P69996, P47079, Q9BRS2, O75419, Q04641, Q12321, Q9UJX3, Q9Y262, P35672, P40413, P15928, P32893, P02748, P12612, P9WQK3, Q00597, P0AG67, P17987, P19483, P25705, Q9BVS4, P42943, P9WP37, A0R202, B7USU2, O15371, P0A6F5, P32780, P50990, P39079, P07251, P49368, Q03776, Q99832, P48643, P50991, Q79VE9, P03728, Q13573, Q9HB96, P78371, O43242, P00401, P39077, Q9BUI4, P40227, Q42290, Q6P1J9, Q8N7H5, P19736, P06576, P0A7I4, Q8DKY0, P00829, P39078, P9WGI1, Q9H3P2, P32481, P39076, C6K2K4, P40016, Q00539, Q9UI10, O43172, Q9BPZ7, P40010, Q1JQ97, Q79PF4, Q9GPE9, P38861, P16140, P35269, O43824, P25382, Q8DMR6, Q96GM5, A3M142, O43660, P00396, P00397, P12268, P20839, P40992, P0ABB0, Q02939, P00830, P18183, P21281, P31408, P62815, O15446, P0AFE8, P49643, P37255, P03222, P06243, P06450, Q04712, P9WNQ9, D0VWR1, P15723, Q03532, Q9UMS4, P32523, P9WNP9, Q96D46, Q9ZU25, Q12874.

Supplementary Table 3. List of the 158 UniProt IDs retrieved after the second stage of our bioinformatic search. These are all UniProt entries from the first-stage list (Supplementary Table 2) that satisfy the additional criteria: (i) at least 3 good-quality PDB models that have a maximum of 10 different polymer entries; (ii) RMSD between two conformers in the ensemble > 4 Å. Search performed in January 2024.

**UniProt entries**
P0DTD1, P11716, Q92736, E9Q401, Q14204, P78527, P36022, Q03131, Q8N2C7, Q03133, P21359, P29994, Q14573, Q9P2D8, P42345, Q8JUX6, P33334, P38111, P07149, P10587, P19097, P16157, Q923J1, Q8IZF0, P04050, Q6R327, P01031, Q8RQE8, Q9Y4B6, O94759, Q00960, P01023, A0A0R4IMY7, P09884, Q96RY7, P0A8T7, P03226, Q2HRA7, K9N5Q8, P0A8V2, Q8NEZ3, Q8N122, P03726, A0QS66, P9WGY7, Q04183, P0DTC2, P59594, Q5XXP3, Q9HBG6, Q12791, P08518, Q8TD43, Q03720, P37871, P37870, O14114, Q9P2L0, P9WGY9, P60281, Q16531, Q8RQE9, Q03660, P53396, P17255, P52002, Q96P20, P53091, Q06337, P24279, P36776, P04133, P17427, P35439, P30665, P02730, P49917, P42260, P19491, P29469, P63284, P38132, P25694, Q80WG5, P15917, P55072, Q9BYF1, Q8R502, P40136, P03747, Q56223, P32794, P29496, P13423, P02786, P0ABH9, P03725, Q04377, P13010, Q6SZW1, A1L314, P32501, P02787, P16452, P0CG48, Q8TAF3, P12754, P00579, P12956, P9WQN5, Q14181, P33755, P09032, Q56403, P03420, P10809, P12538, P47079, Q04641, P35672, P40413, P15928, P32893, P02748, P12612, P17987, P19483, P42943, A0R202, B7USU2, P0A6F5, P50990, P39079, P07251, P49368, Q99832, P48643, P50991, P03728, P78371, P39077, P40227, P00829, P39078, P9WGI1, P32481, P39076, C6K2K4, Q9BPZ7, Q79PF4, P16140, A3M142, P12268, P20839, P00830, P62815, P49643, P06450.

Supplementary Table 4. Dataset of the 47 investigated large protein systems (>2.8k residues) with rich experimental ensembles. The labels and contents of the columns have the same meaning as in Supplementary Table 1. Here we also report the ensemble RMSD (in Å), which provides the average (and standard deviation) of all pairwise RMSDs between the experimental PDB models in the ensemble.

**Protein**	**UniProt ID**	**Oligomeric state**	**Number of common residues**	**Mass (kDa)**	**Ensemble RMSD (Å)**	**Experimental structures used for PCA**
SARS-CoV spike glycoprotein (*SARS_Spike*)	P59594	Homo-trimer	2,801	308	9.0 ± 4.6	***5x58***, *5x5b*, *6ack*, *6nb7*, 5xlr, 6acc, 6acd, 6acg, 6acj, 6crw, 6crx, 6crz, 6cs0, 6cs1, 6nb6, 7akj, 7sg4, 7zh1, 7zh2, 8h0x, 8h0y, 8h0z, 8h10, 8h12, 8h14
Saccharomyces Cerevisiae cell division control protein 48 (*sc_CDC48*)	P25694	Homo-6-mer	2,895	318	4.5 ± 4.3	***8dar***, *6oa9*, *6opc*, 8das, 8dat, 8dau, 8dav, 8daw
SARS-CoV-2 spike glycoprotein (*SARS2_Spike*)	P0DTC2	Homo-trimer	2,910	320	9.3 ± 5.3	***6xr8***, *8cy6*, *7tpr*, *7a94*, 6zge, 6zgi, 7upy, 7krr, 7jjj_1, 7jjj_2, 7upw, 7dwy, 7jji, 7krq, 8cyc, 8cxn, 7x08, 7sbp, 8cyb, 8cya, 7ybj, 7sbs, 8cxq, 7sbt, 7krs, 8cy9, 7qur, 7qus, 8uun, 7e7b, 8uum, 7e7d, 8uul, 8k46, 7wo5, 7woa, 7v26, 7nt9, 7ru1, 7s6i, 7df3, 7n0g, 7ntc, 7rw2, 7l2d, 8k47, 7n0h, 7z3z, 7wz2, 7nta, 7rq6, 7u0x, 7ddd, 7l2e, 7ls9, 7mxp, 7vq0, 7l2f, 7kqb, 7ru2, 7wob, 7wo4_1, 7wo4_2, 7dzw, 7tgy, 8g77, 8g70, 7tgx, 7tb8, 7lqv, 7lrt, 7mm0, 7rbv, 7tyz, 6zp2, 7n9t, 7swx, 8csj, 8cyd, 7cws, 7dzy, 7dzx
Homo Sapiens DNA-dependent protein kinase catalytic subunit (*hs_DNA-PKcs*)	P78527	Monomer	2,993	329	6.2 ± 3.1	***7k19***, *7k0y*, *7k1n*, *7sud*, 7k17_1, 7k17_2, 7k1b, 7k1j, 7k1k, 7otw, 7otm, 7otp, 7otv, 7oty, 7tyr, 6zh4, 6zfp, 6zh2, 6zh6, 6zh8, 6zha 7su3, 7sgl, 7z87, 7z88, 7nfe, 7nfc_1, 7nfc_2, 7lt3_1, 7lt3_2, 5w1r, 5luq_1, 5luq_2, 8eza_1, 8eza_2, 8ez9_1, 8ez9_2, 8bhy_1, 8bhy_2, 8bh3_1, 8bh3_2, 8bhv_1, 8bhv_2

Saccharomyces Cerevisiae DNA replication licensing factor MCM complex (*sc_MCM 6mer*)	A0A6A5Q1S9<br>P24279<br>P30665<br>A0A6A5PUY8<br>P53091<br>P38132	Hetero-6-mer (MCM2-7)	3,047	335	4.3 ± 2.6	***3ja8***, *5v8f*, *7z13_1*, 3jc5, 3jc7, 5bk4_1, 5bk4_2, 5u8t, 6eyc, 6f0l_1, 6f0l_2, 6hv9, 6rqc, 6skl, 7p5z_1, 7p5z_2, 7p30_1, 7p30_2, 7pmk, 7pmn, 7pt6_1, 7pt6_2, 7pt7_1, 7pt7_2, 7qhs, 7v3u_1, 7v3u_2, 7v3v_1, 7v3v_2, 7w8g_1, 7w8g_2, 7z13_2, 8b9a, 8b9b, 8b9c, 8kg6, 8kg8, 8kg9, 8w7m
MERS-CoV spike glycoprotein (*MERS_Spike*)	K9N5Q8	Homo-trimer	3,243	357	10.9 ± 4.6	***7m5e***, *5x59*, *5x5f*, *5w9l*, 5x5c, 7v5j, 7v5k, 7v6n, 7x25, 5w9h, 5w9j, 5w9k, 5w9m, 5w9o
Homo Sapiens Ca^{2+}-activated K-channel (*hs_KCNMA*)	Q12791	Homo-tetramer	3,295	362	4.0 ± 1.8	***6v3g***, *6v38*, 6v22, 6v35, 8gh9, 8ghf, 8ghg
Schizosaccharomyces Pombe ATPase histone chaperone Abo1 (*sp_Abo1*)	O14114	Homo-6-mer	3,322	365	6.5 ± 3.5	***6jpu***, *6jq0*, 6jpq
Escherichia Coli chaperone protein ClpB (*ec_ClpB*)	P63284	Homo-6-mer	3,354	369	3.5 ± 1.2	***5og1***, *6oax*, 5ofo, 6oay, 6qs6, 6qs7, 6qs8
Escherichia Coli ATP-dependent Clp protease ATP-binding ClpA subunit (*ec_ClpA*)	P0ABH9	Homo-6-mer	3,355	369	5.4 ± 3.2	***6w20***, *7uiy*, 6w21, 6w22, 6w23, 6w24, 6w1z, 6uqe, 6uqo, 7uiv, 7uix, 7uiw, 7uiz, 7uj0
Homo Sapiens Ca^{2+}-activated K-channel slowpoke (*hs_SLO*)	Q03720	Homo-tetramer	3,540	389	2.8 ± 2.1	***7pxf***, *7pxe*, 7pxg, 7pxh
Escherichia Coli GroEL chaperonin (*ec_GroEL 7mer*)	P0A6F5	Homo-7-mer	3,626	399	8.1 ± 5.9	***1ss8_1***, *1sx4_1*, 1ss8_2, 1sx4_2, 1kp8_1, 1kp8_2, 1mnf_1, 1mnf_2, 1sx3_1, 1sx3_2, 2yey_1, 2yey_2, 2nwc_1, 2nwc_2, 1xck_1, 1xck_2, 5w0s_1, 5w0s_2, 5opw_1, 5opw_2, 4wgl_1, 4wgl_2, 4wsc_1, 4wsc_2, 4v43_1, 4v43_2, 1grl_1, 1grl_2, 1oel_1, 1oel_2, 1aon_1, 1aon_2, 1pf9_1, 1pf9_2, 1pcq_1, 1pcq_2,

						1svt_1, 1pcq_2, 7pbj_1, 7pbj_2, 7pbx_1, 7pbx_2, 3wvl_1, 3wvl_2, 5opx_1, 5opx_2, 7oxj_1, 7oxj_2, 7oxk_1, 7oxk_2, 7oxl_1, 7oxl_2, 7oxm_1, 7oxm_2, 7ywy_1, 7ywy_2, 8ba7_1, 8ba7_2, 8ba8_1, 8ba8_2, 8ba9_1, 8ba9_2, 8bkz_1, 8bkz_2, 8bl2_1, 8bl2_2, 8bl7_1, 8bl7_2, 8blc_1, 8blc_2, 8bld_1, 8bld_2, 8ble_1, 8ble_2, 8blf_1, 8blf_2, 8bly_1, 8bly_2, 8bmd_1, 8bmd_2, 8bmt_1, 8bmt_2, 8bm0_1, 8bm0_2, 8bm1_1, 8bm1_2, 8bmo_1, 8bmo_2
Saccharomyces Cerevisiae T-complex chaperonin (*sc_TRiC 8mer*)	P12612<br>P39076<br>P39078<br>P40413<br>P39077<br>P42943<br>P47079<br>P39079	1 α-chain<br>1 β-chain<br>1 δ-chain<br>1 ε-chain<br>1 γ-chain<br>1 η-chain<br>1 ϑ-chain<br>1 z-chain	3,645	401	11.9 ± 7.0	***7ylw_1***, *7ylu_1*, *6ks8_1*, 7ylw_2, 7ylu_2, 6ks8_2, 7ylv_1, 7ylv_2, 7ylx_1, 7ylx_2, 7yly_1, 7yly_2, 5gw4_1, 5gw4_2, 5gw5_1, 5gw5_2, 6krd_1, 6krd_2, 6kre_1, 6kre_2, 6ks6_1, 6ks6_2, 6ks7_1, 6ks7_2
Homo Sapiens 60 kDa mitochondrial heat shock protein (*hs_HSP60 7mer*)	P10809	Homo-7-mer	3,668	403	9.0 ± 6.0	***8g7j***, *8g7n_1*, 8g7n_2, 8g7l_1, 8g7l_2, 4pj1_1, 4pj1_2, 6mrc_1, 6mrc_2, 6ht7_1, 6ht7_2, 6mrd, 7azp, 7l7s, 8g7k, 8g7o
Homo Sapiens T-complex chaperonin (*hs_TRiC 8mer*)	P17987<br>P78371<br>P50991<br>P48643<br>P49368<br>Q99832<br>P50990<br>P40227	1 α-chain<br>1 β-chain<br>1 δ-chain<br>1 ε-chain<br>1 γ-chain<br>1 η-chain<br>1 ϑ-chain<br>1 z-chain	3,822	420	5.4 ± 6.4	***8sff_1***, *8sfe_1*, *8sgq_1*, *8i9u_1*, 8sff_2, 8sfe_2, 8sgq_2, 8sgq_2, 8sg8_1, 8sg8_2, 8sg9_1, 8sg9_2, 8sgc_1, 8sgc_2, 8sgl_1, 8sgl_2, 8sh9_1, 8sh9_2, 8sha_1, 8sha_2, 8shd_1, 8shd_2, 8she_1, 8she_2, 8shf_1, 8shf_2, 8shg_1, 8shg_2, 8shl_1, 8shl_2, 8shn_1 8shn_2, 8sho_1, 8sho_2, 8shp_1, 8shp_2, 8shq_1, 8shq_2, 8sht_1, 8sht_2, 6qb8_1, 6qb8_2, 7nvl_1, 7nvl_2, 7nvm_1, 7nvm_2, 7nvn_1, 7nvn_2, 7x3j_1, 7x3j_2, 7x7y_1, x7y_2, 8i1u_1, 8i1u_2, 8ib8_1, 8ib8_2, 7wu7_1,

						7wu7_2, 7x6q_1, 7x6q_2, 7x0v_1, 7x0v_2, 7x0s_1, 7x0s_2, 7lum_1, 7lum_2
Homo Sapiens ATP-citrate synthase (*hs_ACLY*)	P53396	Homo-tetramer	4,032	444	7.2 ± 4.6	***6pof***, *6hxh_1*, *6uia*, *6o0h*, 6hxh_2, 6poe, 6ui9, 6uu, 6uuz, 6uv5, 7rig, 7rkz, 7rmp, 8g1e, 8g1f, 7lla
Homo Sapiens transitional endoplasmic reticulum ATPase (*hs_VCP 6mer*)	P55072	Homo-6-mer	4,131	454	3.9 ± 6.5	***7vcu_1***, *7vcs_1*, 7vcu_2, 7vcs_2, 7vct, 7vcv, 7vcx, 5ftl, 5ftm, 5ftn, 5ftj, 5ftk, 7rlh, 7rlf, 7rlg, 7rli_1, 7rli_2, 7rlj_1, 7rlj_2, 7rl6, 7rl7, 7rl9, 7rla, 7rlb, 7rlc, 7rld, 7lmy, 7r7t, 7bpa, 7bpb, 7bp8, 7bp9, 7k56_1, 7k56_2, 7k57_1, 7k57_2, 7k59, 7jy5, 7y4w, 7y53, 7y59, 8fcl, 8fcm, 8fcn, 8fco, 8fcp, 8fcq, 8fcr, 8ooi
Escherichia Phage T7 portal protein (*T7_Portal*)	P03728	Homo-12-mer	4,212	463	5.2 ± 2.0	***6qx5***, *6qxm*, 7ey6, 7ey8, 7bou, 7bp0, 6r21
Saccharomyces Cerevisiae ATPase family gene 2 protein (*sc_AFG2*)	P32794	Homo-6-mer	4,241	467	7.1 ± 3.5	***7ykk***, *7z11*, *7wbb*, 7ykl, 7ykt, 7ykz, 7wd3
Homo Sapiens Ser/Thr-protein kinase mTOR (*hs_mTOR*)	P42345	Homodimer	4,282	471	5.6 ± 2.5	***6zwm***, *7uxh*, *6bcu*, 7tzo, 7pe7, 7pea, 6bcx, 5zcs
Mus Musculus volume-regulated anion channel subunit LRRC8A (*mm_ LRRC8A*)	Q80WG5	Homo-6-mer	4,308	474	3.7 ± 1.8	***6g9l***, *7p5y*, 6g90, 7p5v, 7p5w, 7p6k, 7p60
Homo Sapiens neurofibromin (*hs_Nf1 – isoform 1*)*	P21359	Homodimer	4,325	476	20.2 ± 15.3	***7r03***, *7r04*, 8e20
Mus Musculus transient receptor potential cation channel subfamily M member 7 (*mm_TRPM7*)	Q923J1	Homo-tetramer	4,388	483	2.2 ± 1.6	***8si2***, *8si5*, 8si3, 8si4, 8si6, 8si7, 8si8, 8sia
Escherichia Coli ATP-dependent Clp	P0ABH9 P0A6G7	ClpA 6-mer ClpP 7-mer	4,598	506	5.9 ± 3.2	***6w20***, *7uiy*, 6w21, 6w1z, 6uqe, 6uqo, 7uiv, 7uix, 7uiw, 7uiz, 7uj0

protease ATP-binding ClpA/P (*ec_ClpA/P 13mer*)						
Homo Sapiens mitochondrial lon protease homolog (*hs_LONP1*)	P36776	Homo-6-mer	4,656	512	7.8 ± 3.7	***7oxo***, *7ng5*, *7ngf*, 7nfy, 7ng4, 7ngl
Escherichia Phage T7 tail tubular protein gp12 (*T7_gp12*)	P03747	Homo-6-mer	4,662	513	5.2 ± 4.7	***7boy***, *7ey7*, 7ey9, 6r21
Mycolicibacterium Smegmatis ATP synthase (*ms_ATPs*)	A0R202<br>A0R200<br>A0R201<br>A0R1Z9<br>A0R206<br>A0R204<br>A0R203<br>A0R205	3 α-chains<br>3 β-chains<br>1 γ-chain<br>1 ε-chain<br>1 *a*-chain<br>1 *b*-chain<br>1 *b*δ-chain<br>9 *c*-chains	4,716	519	10.5 ± 5.4	***7jg5***, *7jg6*, *7jg7*, 7jg8, 7jg9, 7jga, 7njk, 7njl, 7njm 7njn, 7njo, 7njp, 7njq, 7njr, 7njs, 7y5b, 7y5c, 7y5d, 8g08, 8g09, 8g0a, 8g0c, 8g0d, 8g0e
Chikungunya Virus polyprotein P1234 (*cv_P1234*)	Q8JUX6	Homo-12-mer	4,776	525	2.4 ± 1.4	***6z0u_1***, *7y38*, 6z0u_2, 6z0v, 7dop, 7fgg, 7fgh, 7fgi, 7x01, 8aov, 8aow, 8aox_1, 8aox_2, 8jce, 8apx, 8axv
Homo Sapiens neurofibromin (*hs_Nf1 – isoform 2*)*	P21359	Homodimer	4,846	533	15.7 ± 12.9	***7pgr***, *7pgt*, 7pgu
Acinetobacter Baumannii ATP synthase (*ab_ATPs*)	A3M142<br>A3M144<br>A3M137<br>A3M140<br>A3M141<br>A3M145<br>A3M143<br>A3M139	3 α-chains<br>3 β-chains<br>1 *a*-chain<br>2 *b*-chains<br>1 δ-chain<br>1 ε-chain<br>1 γ-chain<br>10 *c*-chains	4,881	537	15.6 ± 0.4	***7p2y***, *7p3n*, *7p3w*
Homo Sapiens α-2-macroglobulin (*hs_A2M*)	P01023	Homo-tetramer	5,082	559	15.0 ± 10.2	***7o7l***, *7o7o*, *7o7p*, *7o7r*, *7o7s*, 7o7q, 6tav
Spinacia Oleracea ATP synthase (*so_ATPs*)	P06450<br>P00825<br>P05435<br>P11402<br>P31853<br>P06453<br>P06451<br>P00833<br>P69447	3 α-chains<br>3 β-chains<br>1 γ-chain<br>1 δ-chain<br>1 *b'*-chain<br>1 *b*-chain<br>1 *a*-chain<br>1 ε-chain<br>14 *c*-chains	5,149	566	14.8 ± 8.2	***6fkf***, *6fkh*, *6fki*, 6vmb, 6vof, 6voh, 6voj, 6vol, 6von

Homo Sapiens transient receptor potential cation channel subfamily M member 2 (*hs_TRPM2*)	O94759	Homo-tetramer	5,208	573	6.8 ± 4.5	***6puo***, *6pus*, 6pur, 6puu, 6mix, 7vq1
Saccharomyces Cerevisiae Ser/Thr-protein kinase MEC1 (*sc_MEC1*)	P38111<br>Q04377	Homodimer + 2 x LCD1	5,264	579	5.2 ± 2.3	***7wzw***, *7wzr*, *6z2x*, 6z3a, 6z2w
Thermus Thermophilus V-type ATPase/synthase (*tt_V-ATPs*)	Q56403<br>Q56404<br>O87880<br>P74903<br>Q5SIT5<br>P74901<br>P74902<br>Q5SIT6<br>Q5SIT7	3 α-chains<br>3 β-chains<br>1 *D*-chain<br>1 *F*-chain<br>2 *V-T* chains<br>2 *E*-chains<br>1 *C*-chain<br>1 *I*-chain<br>12 *K*-chains	5,839	642	12.3 ± 6.2	***6r0w***, *6r0w*, *6r0y*, 6r0z, 6r10
Saccharomyces Cerevisiae DNA replication licensing factor MCM complex (*sc_MCM 12mer*)	A0A6A5Q1S9<br>P24279<br>P30665<br>A0A6A5PUY8<br>P53091<br>P38132	Hetero-12-mer (2 x MCM2-7)	6,094	670	4.8 ± 5.8	***7w8g***, *7z13*, 5bk4, 6f0l, 7p5z, 7p30, 7pt6, 7pt7, 7v3u 7v3v
Escherichia Coli ATP-dependent Clp protease ATP-binding ClpA/P (*ec_ClpA/P 20mer*)	P0ABH9<br>P0A6G7	ClpA 6-mer<br>ClpP 14-mer	6,110	672	2.9 ± 0.9	***6w20***, *6uqo*, 6w21, 6w1z, 6uqe
Escherichia Coli GroEL chaperonin (*ec_GroEL 14mer*)[+]	P0A6F5	Homo-14-mer	7,252	798	10.5 ± 5.0	***1ss8***, *1sx4*, *1sx4_fl*, *3wvl*, 1kp8, 1mnf, 1sx3, 2yey, 2nwc, 1xck, 5w0s, 5opw, 4wgl, 4wsc, 4v43, 5opx, 1grl, 1oel, 7xoj, 7xok, 7xol, 7xom, 7ywy, 8ba7, 8ba8, 8bkz, 8bl2, 8bl7, 8blc, 8bld, 8ble, 8blf, 8bly, 8bmd, 8bmt, 1aon, 1aon_fl, 1pf9, 1pf9_fl, 1pcq, 1pcq_fl, 1svt, 1svt_fl, 7pbj, 7bpj_fl, 7pbx, 7pbx_fl, 8ba9, 8ba9_fl, 8bm0, 8bm0_fl, 8bm1, 8bm1_fl, 8bmo, 8bmo_fl

Homo Sapiens 60 kDa mitochondrial heat shock protein (*hs_HSP60 14mer*)	P10809	Homo-14-mer	7,336	807	7.2 ± 4.6	***8g7l***, *8g7n*, 4pj1, 6mrc, 6ht7
Saccharomyces Cerevisiae T-complex chaperonin (*sc_TRiC 16mer*)	P12612<br>P39076<br>P39078<br>P40413<br>P39077<br>P42943<br>P47079<br>P39079	2 α-chains<br>2 β-chains<br>2 δ-chains<br>2 ε-chains<br>2 γ-chains<br>2 η-chains<br>2 ϑ-chains<br>2 z-chains	7,475	822	13.7 ± 7.5	***7ylw***, *7ylu*, *6ks8*, *6kre*, 7ylv, 7ylx, 7yly, 5gw4, 5gw5, 6krd, 6ks6, 6ks7
Homo Sapiens T-complex chaperonin (*hs_TRiC 16mer*)	P17987<br>P78371<br>P50991<br>P48643<br>P49368<br>Q99832<br>P50990<br>P40227	2 α-chains<br>2 β-chains<br>2 δ-chains<br>2 ε-chains<br>2 γ-chains<br>2 η-chains<br>2 ϑ-chains<br>2 z-chains	7,716	850	5.9 ± 6.8	***8sff***, *8sfe*, *8sgq*, *8i9u*, 8sg8, 8sg9, 8sgc, 8sgl, 8sh9, 8sha, 8shd, 8she, 8shf, 8shg, 8shl, 8shn, 8sho, 8shp, 8shq, 8sht, 6qb8, 7nvl, 7nvm, 7nvn, 7x3j, 7x7y, 8i1u, 8ib8, 7wu7, 7x6q, 7x0v, 7x0s, 7lum
Homo Sapiens inositol 1,4,5-trisphosphate receptor type 3 (*hs_ITPR3*)	Q14573	Homo-tetramer	8,036	884	8.7 ± 5.2	***6dqj***, *7t3t*, *6dr2*, 6dqn, 6dqs, 6dqv, 6dqz, 6dr0, 6dra, 6drc, 7t3p, 7t3q, 7t3r, 8tk8, 8tkd, 8tke, 8tkf, 8tkg, 8tkh, 8tki
Homo Sapiens transitional endoplasmic reticulum ATPase (*hs_VCP 12mer*)	P55072	Homo-12-mer	8,328	916	7.7 ± 4.6	***7vcu***, *7vcs*, 7rli, 7rlj, 7k56, 7k57
Saccharomyces Cerevisiae fatty acid synthase (*sc_FAS 6mer*)	P19097<br>P07149	3 α-subunits<br>3 β-subunits	8,469	932	5.3 ± 5.9	***6u5t_1***, *6ql5_1*, 6u5t_2, 6ql5_2, 6utu_1, 6utu_2, 6ql6_1, 6ql6_2, 6ql7_1, 6ql7_2, 6ql7_3, 6ql7_4, 6ql9_1, 6ql9_2, 6ta1_1, 6ta1_2, 2uv8_1, 2uv8_2, 8prw_1, 8prw_2, 2vkz, 3hmj, 6jsh, 6jsi
Homo Sapiens ryanodine receptor 2 (*hs_RyR2*)	Q92736	Homo-tetramer	15,716	1,729	2.1 ± 0.9	***7ua5***, *7ua4*, 7u9q, 7u9r, 7u9t, 7u9x, 7u9z, 7ua1, 7ua3, 7ua9, 8uq5, 8uxc, 8uxe, 8uxh, 8uq2, 8uxg, 8uq4, 8uxf, 8uxl, 8uxi, 8uq3, 8uxm
Oryctolagus Cuniculus	P11716	Homo-tetramer	16,672	1,834	2.8 ± 1.3	***5tb4***, *5t9v*, 5tb1, 5tb2, 5tb3, 5tb0, 5tap, 5tas, 5tat, 5tau, 5tav, 5ta3, 5tal, 5tan,

ryanodine receptor 1 (*oc_RyR1*)†						5tam, 5taq, 5taw, 5tax, 5tay, 5taz, 5t15, 5t9m, 5t9n, 5t9r, 5t9s
Saccharomyces Cerevisiae fatty acid synthase (*sc_FAS 12mer*)	P19097<br>P07149	6 α-subunits<br>6 β-subunits	20,820	2,290	3.1 ± 1.9	***6u5t***, *6ql5*, 6utu, 6ql6, 6ql7_1, 6ql7_2, 6ql9, 6ta1, 2uv8, 8prw

*For *H. Sapiens* Nf1, we considered separately the two isoforms for which structural information was available from the UniProt ID P21359. +For GroEL 14-mer, we also considered flipped conformations of the asymmetric open-closed conformers (i.e., 1sx4 and 1sx4_fl). These flipped conformations were obtained by swapping the chain labels of the open and closed 7-mer rings. †For the *O. Cuniculus* RyR1, we considered the smaller, yet more consistent, ensemble coming from the work of des Georges et al.[38].

Supplementary Table 5. Additional dataset of 15 large multimeric protein complexes with two experimental conformations used for the simulation of additional transition pathways. Here, Oligomeric State also reports the name of all protein-coding genes for heteromeric complexes. Number of residues reports the number of C^{α} atoms in both end-state PDB models (in parentheses), as well as the number of common residues. The RMSD is evaluated between the two sets of common residues in the two end states.

Protein	UniProt ID	Oligomeric state	Number of residues	Mass (kDa)	RMSD (Å)
Mus Musculus AP2 clathring adaptor (*mm_AP2*)[*]	P17427<br>Q9DBG3<br>P84091<br>P62743<br>Q62314	2 $\alpha 2$-subunits<br>2 $\beta 2$-subunits<br>2 μ-subunits<br>2 σ-subunits<br>2 TGN38 peptides	3,244 (*7rwa*)<br>3,408 (*7rwb*)<br>3,214 (common)	354	38.8
Synechococcus Elongatus RNA polymerase (*se_RNAPol*)	Q31L30<br>Q31N17<br>P42079<br>Q31N15<br>Q31MH9	2 α-subunits<br>1 β-subunit<br>1 γ-subunit<br>1 β'-subunit<br>1 ω-subunit	3,537 (*8syi*)<br>3,574 (*8urw*)<br>3,537	389	10.1
Nematostella Vectensis transienr receptor potential melastatin 2 (*nv_TRPM2*)	A7T1N0	Homo-4-mer	4,282 (*6co7*)<br>3,798 (*9jje*)<br>3,764	414	8.2
Latrodectus Tredecimguttatus alpha-latrotoxin-Lt1a (*lt_Lt1a*)	P23631	Homo-4-mer	4,700 (*9go9*)<br>4,322 (*9goa*)<br>4,320	475	14.0
Homo Sapiens Kv4.2-DPP6S complex (*hs_KV4.2_DPP6S*)	Q9NZV8<br>P42658	KCND2 4-mer<br>DPP6 4-mer	4,580 (*7e8b*)<br>5,576 (*7e8h*)<br>4,562	502	4.9
Saccharomyces Cerevisiae plasma membrane ATPase 1 (*sc_PMA1*)	P05030	Homo-6-mer	4,824 (*7vh5*)<br>4,626 (*7vh6*)<br>4,602	506	12.5
Escherichia Coli K-dependent mechanosensitive channel (*ec_MscK*)	P77338	Homo-7-mer	5,061 (*7uw5*)<br>4,991 (*7ux1*)<br>4,893	538	11.8
Mus Musculus AMPA receptor (*mm_AMPAR*)[+]	P23818<br>P23819<br>O35089<br>Q8VHW2<br>-<br>-<br>-	Gria1 2-mer<br>Gria2 2-mer<br>Cnih2 2-mer<br>Cacng8 2-mer<br>11B8 scFv<br>15F1 Fab light chain<br>15F1 Fab heavy chain	4,990 (*7ldd*)<br>4,990 (*7lde*)<br>4,990	549	7.8
Homo Sapiens intraflagellar transport complex A (*hs_IFT-A*)	Q96RY7<br>Q9HBG6<br>Q8NEZ3<br>Q9P2L0<br>Q96FT9<br>Q7Z4L5	IFT140<br>IFT122<br>IFT144<br>IFT121<br>IFT143<br>IFT139	6,424 (*8bbg*)<br>5,564 (*8fgw*)<br>5,537	609	27.8

Homo Sapiens essential mediator (*hs_MED*)[**]	Q15648<br>Q9NPJ6<br>O75586<br>O43513<br>Q96G25<br>Q9NWA0<br>Q9BTT4<br>Q9P086<br>Q9UHV7<br>O60244<br>Q96RN5<br>Q9Y2X0<br>Q9NVC6<br>Q9BUE0<br>A0JLT2<br>Q9H944<br>Q13503<br>Q15528<br>Q9ULK4<br>O75448<br>Q71SY5<br>Q6P2C8<br>Q9H204<br>Q9NX70<br>Q96HR3<br>Q9Y3C7<br>-	MED1<br>MED4<br>MED6<br>MED7<br>MED8<br>MED9<br>MED10<br>MED11<br>MED13<br>MED14<br>MED15<br>MED16<br>MED17<br>MED18<br>MED19<br>MED20<br>MED21<br>MED22<br>MED23<br>MED24<br>MED25<br>MED27<br>MED28<br>MED29<br>MED30<br>MED31<br>Unknown peptide	7,781 (*8t9d*)<br>7,627 (*8trh*)<br>7,231	795	5.6
Mus Musculus low-density lipoprotein receptor-related protein 2 (*mm_LRP2*)	A2ARV4	Homo-2-mer	8,314 (*8em4*)<br>9,590 (*8em7*)<br>7,620	838	57.4
Homo Sapiens anaphase-promoting complex (*hs_APC/C*)[***]	Q9H1A4<br>Q9UJX6<br>Q9UJX5<br>Q9UJX4<br>Q9UJX3<br>Q9UM13<br>Q9NYG5<br>Q9BS18<br>Q2NKV2<br>P60006<br>Q96DE5<br>Q8NHZ8<br>Q13042<br>Q12834<br>Q9UJX2<br>P30260<br>P34244	APC subunit 1<br>APC subunit 2<br>APC subunit 4<br>APC subunit 5<br>2 APC subunit 7<br>APC subunit 10<br>APC subunit 11<br>APC subunit 13<br>APC subunit 13<br>APC subunit 15<br>APC subunit 16<br>2 APC subunit CDC26<br>2 CDC16 homologs<br>CDC20 homolog<br>2 CDC23 homologs<br>2 CDC27 homologs<br>Ser/Thr kinase HSL1	8,352 (*5g04*)<br>8,053 (*5g05*)<br>7,756	853	9.9
Mus Musculus macrophage-expressed gene 1 protein (*mm_Mpeg1*)	A1L314	16 Mpeg1	8,528 (*8a1d*)<br>8,528 (*8a1s*)<br>8,528	938	17.6

Homo Sapiens integrator complex (*hs_INT)*[****]	P30153<br>P67775<br>Q8N201<br>Q9H0H0<br>Q68E01<br>Q96HW7<br>Q6P9BP<br>Q9UL03<br>Q9NVH2<br>Q75QN2<br>Q9NV88<br>Q9NVR2<br>Q5TA45<br>Q9NVM9<br>Q96SY0<br>Q96N11<br>Q9BQ15<br>Q9NRY2<br>-<br>-<br>I3LJR4	PPP2R1A<br>PPP2CA<br>INTS1<br>INTS2<br>INTS3<br>INTS4<br>INTS5<br>INTS6<br>INTS7<br>INTS8<br>INTS9<br>INTS10<br>INTS11<br>INTS13<br>INTS14<br>INTS15<br>NABP2<br>INIP<br>*Trichoplusia Ni* DSS1<br>*Sus Scrofa* unknown peptide<br>*Sus Scrofa* POLR2A	11,105 (*8rc4*)<br>10,105 (*8rbz*)<br>9,401	1,034	6.1
Escherichia Phage Lambda tail (*ep_Tail*)	P03733<br>P03737<br>P03749<br>P03738<br>P03736<br>P03730	24 lambdap13<br>6 lambdap17<br>3 lambdap21<br>3 lambdap18<br>3 lambdap16<br>3 lambdap20	10,248 (*8iyk*)<br>10,248 (*8iyl*)<br>10,248	1,127	17.6

[*]The two Trans-Golgi network integral membrane protein TGN38 peptides are present in 7rwa but not in 7rwb. [**]MED19 (A0JLT2) is only present in 8trh, while MED25 (Q71SY5) is only present in 8t9d. [***]*H. Sapiens* APC subunit 13 (Q9BS18), *H. Sapiens* CDC20 homolog (Q12834), and *S. Cerevisiae* Ser/Thr protein kinase HSL1 (P34244) are present in 5g04 but not in 5g05. Conversely, *B. Taurus* ACP subunit 13 (Q2NKV2) and a 15-mer unidentified peptide are present in 5g05 and not in 5g04. [****]INST3 (Q68E01), NABP2 (Q9BQ15), INIP (Q9NRY2), and the *S. Scrofa* POLR2A (I3LJR4) are only present in 8rbz and not in 8rc4. [+]The conformers of AMPA receptor also include fragment antigen-binding (Fab) regions of an antibody, that are not classified by a UniProt ID.

Supplementary Table 6. Results from PCA of the 47 large-protein ensembles. For each system, the number of conformations included in the ensemble, the amount of variance (%) captured by the first two PCs, and the total number of PCs needed to describe at least 90% of the variance are reported.

Protein	Number of conformers in the ensemble	PC1 (%)	PC2 (%)	$PC_{90\%}$ (-)	Protein	Number of conformers in the ensemble	PC1 (%)	PC2 (%)	$PC_{90\%}$ (-)
SARS_Spike	25	58.95	18.62	3	hs_LONP1	6	68.16	28.87	2
sc_CDC48	8	88.52	8.75	2	T7_gp12	4	98.84	0.73	1
SARS2_Spike	82*	70.92	13.6	3	ms_ATPs	24	53.06	41.40	2
hs_DNA-PKcs	43	52.95	21.17	4	cv_P1234	16	81.54	15.39	2
sc_MCM 6mer	39	40.90	25.77	5	hs_Nf1 (isoform 2)	3	99.92	0.08	1
MERS_Spike	14	52.25	34.57	3	ab_ATPs	3	57.44	42.56	2
hs_KCNMA	7	70.99	23.86	2	hs_A2M	7	77.37	14.63	2
sp_Abo1	3	93.60	6.40	1	so_ATPs	9	54.74	44.54	2
ec_ClpB	7	53.78	24.63	4	hs_TRPM2	6	93.06	5.50	1
ec_ClpA	14	88.40	5.12	2	sc_MEC1	5	60.41	30.60	2
hs_SLO	4	94.54	4.96	1	tt_V-ATPs	5	56.62	40.52	2
ec_GroEL 7mer	88	74.25	17.98	2	sc_MCM 12mer	10	93.10	5.58	1
sc_TRiC 8mer	24	79.80	14.61	2	ec_ClpA/P 20mer	5	69.27	19.17	3
hs_HSP60 7mer	16	83.63	10.65	2	ec_GroEL 14mer	55	45.82	31.75	3
hs_TRiC 8mer	66	92.64	3.22	1	hs_HSP60 14mer	5	81.12	15.93	2
hs_ACLY	16	57.12	29.97	3	sc_TRiC 16mer	12	80.76	13.15	2
hs_VCP 6mer	50	78.36	6.01	5	hs_TRiC 16mer	33	92.91	2.98	1
T7_Portal	7	56	34.48	2	hs_ITPR3	20	73.25	18.08	2
sc_AFG2	7	74.37	14.2	3	hs_VCP 12mer	6	94.12	4.08	1
hs_mTOR	8	60.63	29.37	2	sc_FAS 6mer	24	83.50	13.99	2
mm_ LRRC8A	7	66.21	20.7	3	hs_RyR2	22	71.88	6.73	7
hs_Nf1 (isoform 1)	3	99.44	0.56	1	oc_RyR1	25	75.31	18.01	2
mm_TRPM7	8	79.55	7.59	3	sc_FAS 12mer	10	87.87	6.26	2
ec_ClpA/P 13mer	11	88.27	5.12	2					

*The actual number of conformations for the *SARS-CoV-2* spike glycoprotein available in the PDB is much higher than 82. However, here we only retained PDB models with less than 60 missing residues and those associated with the canonical amino acid sequence. Considering additional structures would have just reduced the number of common residues available for PCA and would not have provided additional insights into the main conformational clusters.

Supplementary Table 7. RMSD values, collectivity degrees, computing times, and convergences for all transition pathways of the large protein ensemble dataset simulated with eBDIMS2.

Protein	Number of residues (-)	Transition pathway (Reference – Target PDB)	Transition RMSD (Å)	Collectivity degree (-)	Computing time (hours)	Convergence RMSD (Å)
SARS_Spike	2,801	5x58-5x5b	9.5	0.10	0.85	0.55
		5x5b-5x58			0.52	0.73
		5x58-6ack	11.0	0.11	1.69	0.75
		6ack-5x58			1.08	0.67
		5x58-6nb7	17.6	0.23	1.21	0.96
		6nb7-5x58			0.99	0.83
		6ack-5x5b	14.3	0.19	1.49	0.82
		5x5b-6ack			1.35	0.74
		6nb7-6ack	13.7	0.17	1.32	0.59
		6ack-6nb7			1.49	0.64
		6nb7-5x5b	14.4	0.20	1.07	0.68
		5x5b-6nb7			1.27	0.75
sc_CDC48	2,895	8dar-6oa9	5.2	0.69	0.59	0.80
		6oa9-8dar			0.64	0.79
		8dar-6opc	11.5	0.86	1.09	0.78
		6opc-8dar			1.23	0.74
SARS2_Spike	2,910	6xr8-8cy6	22.6	0.23	1.51	0.82
		8cy6-6xr8			1.32	0.75
		6xr8-7tpr	11.6	0.19	1.24	0.61
		7tpr-6xr8			0.80	0.70
		6xr8-7a94	9.1	0.18	1.16	0.56
		7a94-6xr8			0.62	0.75
		7a94-7tpr	13.3	0.23	1.38	0.61
		7tpr-7a94			1.17	0.61
		7a94-8cy6	18.7	0.17	1.55	0.58
		8cy6-7a94			1.39	0.54
		8cy6-7tpr	15.4	0.19	1.13	0.72
		7tpr-8cy6			1.27	0.55
hs_DNA-PKcs	2,993	7k19-7k1n	5.8	0.17	0.31	0.78
		7k1n-7k19			0.34	0.77
		7k1n-7k0y	4.8	0.58	0.32	0.79
		7k0y-7k1n			0.31	0.78
		7k19-7k0y	7.8	0.46	0.48	0.79
		7k0y-7k19			0.49	0.76
		7k0y-7sud	13.4	0.63	1.17	0.54
		7sud-7k0y			2.03	0.52
sc_MCM 6mer	3,047	3ja8-5v8f	10.2	0.64	0.71	0.78
		5v8f-3ja8			0.71	0.79
		3ja8-7z13	5.7	0.75	0.42	0.78
		7z13-3ja8			0.45	0.79
MERS_Spike	3,243	7m5e-5x59	19.7	0.22	3.14	0.80
		5x59-7m5e			2.85	0.80
		7m5e-5x5f	11.5	0.13	4.19	0.86
		5x5f-7m5e			3.68	0.75
		7m5e-5w9l	12.2	0.12	1.85	0.56

		5w9l-7m5e			1.09	0.65
		5w9l-5x5f	14.9	0.19	3.59	1.07
		5x5f-5w9l			3.80	0.81
		5x59-5w9l	14.6	0.18	2.76	0.80
		5w9l-5x59			2.75	1.02
		5x59-5x5f	14.7	0.15	0.93	0.61
		5x5f-5x59			1.05	0.67
hs_KCNMA	3,295	6v3g-6v38	4.6	0.53	0.42	0.79
		6v38-6v3g			0.45	0.79
sp_Abo1	3,322	6jpu-6jq0	8.5	0.53	1.48	0.77
		6jq0-6jpu			1.33	0.78
ec_ClpB	3,354	5og1-6oax	5.3	0.65	0.73	0.79
		6oax-5og1			0.78	0.80
ec_ClpA	3,355	6w20-7uiy	8.8	0.63	0.81	0.78
		7uiy-6w20			0.89	0.79
hs_SLO	3,540	7pxf-7pxe	4.7	0.52	0.42	0.80
		7pxe-7pxf			0.43	0.79
ec_GroEL 7mer	3,626	1ss8-1sx4	15.2	0.56	1.20	0.55
		1sx4-1ss8			1.22	0.60
sc_TRiC 8mer	3,645	7ylw-7ylu	21.3	0.34	2.18	1.29
		7ylu-7ylw			1.87	1.14
		7ylw-6ks8	15.1	0.42	1.93	0.84
		6ks8-7ylw			1.77	0.82
		7ylu-6ks8	12.5	0.14	1.26	0.53
		6ks8-7ylu			2.04	0.58
hs_HSP60 7mer	3,668	8g7j-8g7n	15.8	0.58	1.53	0.55
		8g7n-8g7j			1.25	0.78
hs_TRiC 8mer	3,822	8sff-8i9u	16.0	0.56	2.21	0.95
		8i9u-8sff			2.49	0.90
		8sff-8sgq	11.5	0.33	1.57	0.71
		8gsq-8sff			1.44	0.79
		8sgq-8i9u	9.4	0.46	1.37	1.00
		8i9u-8sgq			1.56	0.96
hs_ACLY	4,032	6pof-6hxh	12.5	0.60	1.74	0.79
		6hxh-6pof			2.03	0.82
		6uia-6o0h	20.2	0.50	2.54	0.96
		6o0h-6uia			3.42	1.22
hs_VCP 6mer	4,131	7vcu-7vcs	9.6	0.30	1.73	0.78
		7vcs-7vcu			2.09	0.79
T7_Portal	4,212	6qx5-6qxm	6.9	0.21	1.75	0.79
		6qxm-6qx5			1.88	0.79
sc_AFG2	4,241	7ykk-7z11	10.9	0.72	3.27	1.00
		7z11-7ykk			3.50	0.90
		7ykk-7wbb	10.8	0.73	3.18	0.85
		7wbb-7ykk			3.30	0.80
hs_mTOR	4,282	6zwm-6bcu	9.4	0.83	1.36	0.75
		6bcu-6zwm			1.22	0.78
		6zwm-7uxh	7.1	0.67	1.03	0.80
		7uxh-6zwm			1.00	0.79
mm_ LRRC8A	4,308	6g9l-7p5y	6.1	0.74	0.78	0.88
		7p5y-6g9l			0.80	0.92
hs_Nf1	4,325	7r03-7r04	29.2	0.26	7.80	1.66

(isoform 1)		7r04-7r03			7.34	1.53
mm_TRPM7	4,388	8si2-8si5	4.9	0.69	0.90	0.80
		8si5-8si2			0.90	0.80
ec_ClpA/P 13mer	4,500	6w20-7uiy	8.9	0.68	1.90	0.77
		7uiy-6w20			1.82	0.79
hs_LONP1	4,656	7oxo-7ng5	10.4	0.73	2.31	0.77
		7ng5-7oxo			2.24	0.77
		7oxo-7ngf	11.2	0.72	2.60	0.75
		7ngf-7oxo			2.45	0.75
T7_gp12	4,662	7boy-7ey7	9.5	0.57	2.17	0.78
		7ey7-7boy			2.67	0.8
*ms_ATPs**	4,716	7jg5-7jg6	14.0	0.27	9.09	1.15
		7jg6-7jg7	16.0	0.34	7.61	1.12
		7jg7-7jg5	13.6	0.33	6.65	1.62
cv_P1234	4,776	6z0u-7y38	4.2	0.90	0.62	0.79
		7y38-6z0u			0.66	0.80
hs_Nf1 (isoform 2)	4,846	7pgr-7pgt	23.1	0.11	5.04	0.90
		7pgt-7pgr			6.39	0.99
*ab_ATPs**	4,881	7p2y-7p3n	15.4	0.30	9.09	2.13
		7p3n-7p3w	16.0	0.31	10.04	1.95
		7p3w-7p2y	15.4	0.30	9.57	1.91
hs_A2M	5,082	7o7l-7o7o	19.2	0.29	5.20	0.88
		7o7o-7o7l			6.40	1.00
		7o7l-7o7s	26.0	0.43	5.74	1.36
		7o7s-7o7l			6.48	1.50
		7o7l-7o7p	28.8	0.46	5.41	1.51
		7o7p-7o7l			6.09	1.77
		7o7o-7o7s	15.8	0.32	5.47	0.89
		7o7s-7o7o			5.13	0.99
		7o7o-7o7p	19.4	0.40	4.78	0.90
		7o7p-7o7o			6.32	1.00
		7o7s-7o7r	11.9	0.16	4.17	0.66
		7o7r-7o7s			5.00	0.75
*so_ATPs**	5,149	6fkf-6fkh	19.5	0.36	14.50	1.40
		6fkh-6fki	22.6	0.42	13.39	0.94
		6fki-6fkf	20.4	0.34	13.59	1.51
hs_TRPM2	5,164	6puo-6pus	10.8	0.63	2.42	0.77
		6pus-6puo			2.47	0.78
sc_MEC1	5,264	7wzw-7wzr	7.7	0.55	10.21	1.46
		7wzr-7wzw			8.93	1.54
		7wzw-6z2x	7.6	0.55	8.71	0.84
		6z2x-7wzw			6.56	0.90
*tt_V-ATPs**	5,839	6qum-6r0w	16.3	0.28	11.93	1.50
		6r0w-6qum			14.63	1.39
		6r0w-6r0y	16.4	0.31	13.50	1.39
		6r0y-6r0w			13.78	1.35
		6r0y-6qum	16.0	0.28	13.96	1.54
		6qum-6r0y			13.65	1.57
sc_MCM 12mer	6,094	7w8g-7z13	16.4	0.81	6.63	0.94
		7z13-7w8g			5.63	0.83
	6,110	6w20-6uqo	4.0	0.75	3.12	0.80

ec_ClpA/P 20mer		6uqo-6w20			2.66	0.80
ec_GroEL 14mer	7,252	1ss8-1sx4	11.4	0.29	4.53	0.94
		1sx4-1ss8			4.44	0.99
		1ss8-1sx4$_{fl}$	11.4	0.29	4.56	0.95
		1sx4$_{fl}$-1ss8			4.15	1.00
		1ss8-3wvl	12.9	0.46	8.11	0.98
		3wvl-1ss8			7.33	0.98
		1sx4-3wvl	12.8	0.38	5.82	0.99
		3wvl-1sx4			5.49	0.99
		1sx4$_{fl}$-3wvl	12.8	0.38	5.78	0.99
		3wvl-1sx4$_{fl}$			5.51	0.99
		1sx4-1sx4$_{fl}$	15.1	0.59	5.45	0.96
		1sx4$_{fl}$-1sx4			5.39	0.95
hs_HSP60 14mer	7,336	8g7l-8g7n	12.3	0.25	4.92	0.80
		8g7n-8g7l			4.77	0.79
sc_TRiC 16mer	7,475	7ylw-7ylu	23.3	0.30	11.85	1.02
		7ylu -7ylw			12.89	0.95
		7ylw-6ks8	16.3	0.40	8.06	0.98
		6ks8-7ylw			7.76	0.85
		7ylu -6ks8	13.9	0.12	7.95	0.65
		6ks8-7ylu			6.74	0.70
hs_TriC 16mer	7,716	8sff-8i9u	17.6	0.51	10.88	1.00
		8i9u-8sff			10.62	0.97
		8sff-8sgq	12.0	0.31	7.87	0.76
		8gsq-8sff			6.64	0.78
		8sgq-8i9u	10.2	0.47	7.77	1.06
		8i9u-8sgq			7.75	1.05
hs_ITPR3	8,036	6dqj-7t3t	9.2	0.66	5.53	0.80
		7t3t-6dqj			5.49	0.79
		6dqj-6dr2	16.5	0.58	7.76	1.03
		6dr2-6dqj			7.77	1.03
		7t3t-6dr2	13.7	0.77	8.89	0.85
		6dr2-7t3t			8.43	0.83
hs_VCP 12mer	8,328	7vcu-7vcs	10.2	0.21	8.79	0.99
		7vcs-7vcu			8.29	0.95
sc_FAS 6mer	8,469	6u5t-6ql5	5.0	0.44	3.25	0.79
		6ql5-6u5t			3.16	0.80
hs_RyR2	15,716	7ua5-7ua4	4.0	0.62	23.99	1.21
		7ua4-7ua5			24.35	1.20
oc_RyR1	16,672	5tb4-5t9v	7.7	0.66	43.65	1.27
		5t9v-5tb4			41.02	1.23
sc_FAS 12mer	20,820	6u5t-6ql5	5.1	0.44	49.26	0.86
		6ql5-6u5t			49.58	0.85

*Rigidity constraints applied to the rotor chains of all ATP synthases (Supplementary Fig. 5).

Supplementary Table 8. RMSD values, collectivity degrees, and computing times for the transition pathways of the additional dataset of two-state proteins simulated with eBDIMS2.

Protein	Transition pathway (Reference – Target PDB)	Number of reference residues	Transition RMSD (Å)	Collectivity degree (-)	Computing time (hours)	Convergence RMSD (Å)
mm_AP2	7rwa-7rwb	3,244	38.8	0.78	4.38	1.95
	7rwb-7rwa	3,408			5.16	1.98
se_RNAPol	8syi-8urw	3,537	10.1	0.40	2.38	1.00
	8urw-8syi	3,574			2.31	0.90
nv_TRPM2	6co7-9jje	4,282	8.2	0.51	1.56	0.73
	9jje-6co7	3,798			1.21	0.76
lt_Lt1a	9go9-9goa	4,700	14.0	0.10	8.53	1.65
	9goa-9go9	4,320			3.86	0.83
hs_KV4.2_DPP6S	7e8b-7e8h	4,580	4.9	0.61	0.93	0.77
	7e8h-7e8b	4,562			0.90	0.73
sc_PMA1	7vh5-7vh6	4,824	12.5	0.83	3.05	0.83
	7vh6-7vh5	4,626			2.41	0.76
ec_MscK	7uw5-7ux1	5,061	11.8	0.89	2.58	0.75
	7ux1-7uw5	4,991			2.61	0.72
mm_AMPAR	7ldd-7lde	4,990	7.8	0.72	4.33	1.10
	7lde-7ldd	4,990			5.42	1.06
hs_IFT-A	8bbg-8fgw	6,424	27.8	0.47	13.10	2.32
	8fgw-8bbg	5,564			9.58	1.47
hs_MED	8t9d-8trh	7,781	5.6	0.36	17.69	2.11
	8trh-8t9d	7,627			17.83	2.11
mm_LRP2	8em4-8em7	8,314	57.4	0.74	49.64	4.14
	8em7-8em4	9,590			57.96	14.99
hs_APC/C	5g04-5g05	8,352	9.9	0.20	7.25	1.07
	5g05-5g04	8,053			8.90	1.07
mm_Mpeg1	8a1d-8a1s	8,528	17.6	0.43	14.36	1.09
	8a1s-8a1d	8,528			12.98	1.06
hs_INT	8rc4-8rbz	11,105	6.1	0.56	25.93	1.26
	8rbz-8rc4	10,105			23.52	1.30
ep_Tail	8iyk-8iyl	10,248	17.6	0.60	41.85	2.43
	8iyl-8iyk	10,248			44.30	2.43

Supplementary Table 9. MolProbity[24] assessment of RBP intermediate conformations generated by the different path-sampling methods. Coarse-grained models have been reconstructed via cg2all[23].

Path-sampling method	Transition direction	RMSD from starting/target state (Å)*	Ramachandran outlier/favored (%)	Rotamer outliers (%)	Clash-score	RMS bonds	RMS angles	MP score+
eBDIMS2	1ba2-2dri	3.6/3.6	0.7/95.9	1.5	125	0.024	2.02	2.98
	2dri-1ba2	3.7/3.6	0.4/94.8	1.5	112	0.026	1.95	3.01
eBDIMS	1ba2-2dri	2.8/4.7	4.1/85.8	1.0	164	0.055	5.81	3.32
	2dri-1ba2	2.8/4.9	2.6/91.1	2.0	96	0.038	3.35	3.20
iMODs	1ba2-2dri	3.2/3.1	0.7/98.1	1.0	25	0.012	0.85	1.89
	2dri-1ba2	4.5/3.7	0.4/97.8	1.0	184	0.014	1.07	2.78
GOdMD	1ba2-2dri	0.4/6.0	0.4/98.1	0.5	27	0.043	1.66	1.92
	2dri-1ba2	0.3/6.1	0.7/98.1	1.0	30	0.033	1.96	1.97
NGENI	1ba2-2dri	3.1/3.2	1.5/97.0	1.5	43	0.028	2.59	2.42
	2dri-1ba2	3.3/3.3	1.9/94.2	1.0	47	0.060	4.14	2.55
ICONGENI	1ba2-2dri	3.0/3.2	0.4/98.1	1.5	32	0.012	0.88	2.12
	2dri-1ba2	3.2/3.3	0.7/95.9	0.5	37	0.015	1.16	2.33
Climber	1ba2-2dri	4.5/2.5	0.4/96.7	0.0	22	0.012	0.92	2.04
	2dri-1ba2	4.9/2.4	0.4/97.4	0.5	24	0.013	0.88	1.98
NOLB	1ba2-2dri	3.6/2.7	1.1/97.8	1.0	27	0.018	1.48	1.97
	2dri-1ba2	3.9/2.9	3.0/94.7	0.5	70	0.058	3.56	2.68
ENI	-	3.1/3.1	0.4/98.5	0.5	24	0.016	1.01	1.88
aANM	-	3.3/3.1	0.8/97.4	0.0	50	0.024	1.87	2.30
ANMPathway	-	3.5/3.3	1.1/97.8	0.5	31	0.021	2.24	2.03
MinActionPath2	-	3.1/3.5	5.3/87.0	2.6	115	0.011	6.61	3.45

*For linear methods, starting and target structures for RMSD calculations are the same in both directions. +MP score stands for MolProbity score.

Supplementary Table 10. MolProbity[24] assessment of RNaseIII intermediate conformations generated by the different path-sampling methods. Coarse-grained models have been reconstructed via cg2all[23].

Path-sampling method	Transition direction	RMSD from starting/target state (Å)	Ramachandran outlier/favored (%)	Rotamer outliers (%)	Clash-score	RMS bonds	RMS angles	MP score
eBDIMS2	1yyo-1yyw	10.9/9.8	3.3/88.1	2.8	205	0.0283	2.65	3.71
	1yyw-1yyo	10.8/9.6	3.0/85.1	1.4	217	0.0307	3.42	3.57
eBDIMS	1yyo-1yyw	8.5/11.9	8.1/78.2	1.4	190	0.0504	5.07	3.61
	1yyw-1yyo	10.0/10.7	18.9/65.3	1.7	278	0.078	7.46	3.95
iMODs	1yyo-1yyw	14.1/9.8	0.2/96.5	0.6	27	0.011	0.91	2.14
	1yyw-1yyo	14.3/10.8	0.2/95.8	0.6	55	0.011	0.80	2.51
GOdMD	1yyo-1yyw	0.7/16.9	0.7/96.0	0.8	31	0.022	1.63	2.25
	1yyw-1yyo	0.8/16.8	0.7/96.0	0.3	35	0.022	1.34	2.30
NGENI	1yyo-1yyw	7.1/12.1	1.9/94.0	0.3	44	0.049	3.31	2.52
	1yyw-1yyo	10.3/9.3	5.5/86.4	0.0	99	0.111	5.76	3.10
ICONGENI	1yyo-1yyw	7.4/12.0	2.3/93.5	0.3	37	0.012	0.85	2.48
	1yyw-1yyo	10.8/8.8	6.1/85.0	0.6	73	0.062	4.26	2.99
Climber	1yyo-1yyw	11.7/9.1	0.5/96.3	0.3	31	0.011	0.92	2.23
	1yyw-1yyo	12.2/8.8	1.2/93.5	0.3	33	0.012	0.95	2.43
NOLB	1yyo-1yyw	11.4/9.2	12.2/80.2	1.4	251	0.118	10.49	3.81
	1yyw-1yyo	11.1/9.3	9.6/83.8	1.1	195	0.127	7.92	3.47
ENI	-	7.8/10.3	14.5/77.2	1.4	178	0.097	12.12	3.60
aANM	-	8.5/11.4	10.4/79.9	1.7	260	0.126	8.51	3.78
ANMPathway	-	-	-	-	-	-	-	-
MinActionPath2	-	9.3/10.9	22.0/57.4	3.1	362	0.168	11.94	4.32

Supplementary Table 11. MolProbity[24] assessment of SERCA intermediate conformations generated by the different path-sampling methods. Coarse-grained models have been reconstructed via cg2all[23].

Path-sampling method	Transition direction	RMSD from starting/target state (Å)	Ramachandran outlier/favored (%)	Rotamer outliers (%)	Clash-score	RMS bonds	RMS angles	MP score
eBDIMS2	2c9m-1t5s	7.9/8.0	4.0/86.0	2.3	208	0.032	3.07	3.70
	1t5s-2c9m	8.4/8.3	2.9/88.3	2.1	151	0.031	2.92	3.48
eBDIMS	2c9m-1t5s	6.0/9.8	6.8/31.4	1.8	183	0.038	3.23	3.64
	1t5s-2c9m	6.5/10.5	5.4/83.5	3.8	148	0.037	3.64	3.75
iMODs	2c9m-1t5s	8.3/7.3	0.8/96.0	1.3	52	0.015	0.97	2.56
	1t5s-2c9m	9.5/9.8	0.9/94.3	1.0	58	0.014	1.01	2.63
GOdMD	2c9m-1t5s	0.7/13.6	0.9/95.4	1.7	50	0.030	1.55	2.67
	1t5s-2c9m	0.6/13.6	1.3/93.5	0.9	52	0.024	1.38	2.62
NGENI	2c9m-1t5s	7.3/7.2	1.1/93.4	1.2	61	0.034	2.48	2.74
	1t5s-2c9m	7.3/7.3	3.6/88.2	1.0	78	0.079	4.34	2.97
ICONGENI	2c9m-1t5s	7.3/7.2	0.8/94.7	1.6	56	0.015	1.21	2.74
	1t5s-2c9m	7.5/7.1	1.8/91.7	1.4	66	0.027	1.39	2.91
Climber	2c9m-1t5s	10.1/5.4	0.7/94.8	0.8	36	0.013	1.08	2.39
	1t5s-2c9m	10.3/5.4	1.6/94.6	1.3	34	0.014	1.08	2.47
NOLB	2c9m-1t5s	8.3/7.0	4.0/89.2	1.8	101	0.051	4.57	3.24
	1t5s-2c9m	9.4/9.3	5.8/84.6	1.6	157	0.109	7.39	3.47
ENI	-	7.2/7.0	3.5/92.4	0.8	83	0.049	5.28	2.86
aANM	-	7.4/6.6	14.1/76.3	1.9	244	0.090	9.35	3.84
ANMPathway	-	9.8/6.4	1.7/94.1	1.3	58	0.025	2.14	2.72
MinActionPath2	-	7.0/7.8	14.7/70.8	2.6	193	0.150	9.79	3.89

Supplementary Table 12. MolProbity[24] assessment of GroEL 7-mer intermediate conformations generated by the different path-sampling methods. Coarse-grained models have been reconstructed via cg2all[23].

Path-sampling method	Transition direction	RMSD from starting/target state (Å)	Ramachandran outlier/favored (%)	Rotamer outliers (%)	Clash-score	RMS bonds	RMS angles	MP score
eBDIMS2	1ss8-1sx4	8.3/7.7	3.4/88.3	3.0	160	0.032	2.93	3.62
	1sx4-1ss8	8.1/7.7	2.7/89.6	2.2	161	0.034	2.82	3.49
eBDIMS	1ss8-1sx4	7.8/9.2	4.6/85.6	2.4	126	0.037	3.52	3.50
	1sx4-1ss8	7.8/8.7	4.6/85.1	1.9	141	0.034	2.90	3.48
iMODs	1ss8-1sx4	9.2/9.4	0.0/98.2	0.7	31	0.013	1.00	1.98
	1sx4-1ss8	9.4/8.9	0.2/97.9	0.9	52	0.014	1.05	2.22
GOdMD	1ss8-1sx4	0.3/15.1	0.1/97.9	1.0	34	0.021	1.52	2.04
	1sx4-1ss8	0.4/15.1	0.1/97.7	0.5	31	0.020	1.50	2.04
NGENI	1ss8-1sx4	8.4/10.4	2.1/94.7	1.5	50	0.050	3.81	2.66
	1sx4-1ss8	10.5/7.1	2.7/93.3	0.8	70	0.054	4.02	2.75
ICONGENI	1ss8-1sx4	8.4/10.0	0.3/97.0	1.3	40	0.014	1.11	2.34
	1sx4-1ss8	10.7/6.9	0.3/96.3	1.0	52	0.014	1.05	2.44
Climber	1ss8-1sx4	-	-	-	-	-	-	-
	1sx4-1ss8	-	-	-	-	-	-	-
NOLB	1ss8-1sx4	10.1/10.8	1.1/95.6	1.0	40	0.098	4.79	2.39
	1sx4-1ss8	8.1/11.1	1.2/93.8	0.8	70	0.052	3.52	2.73
ENI	-	7.8/7.9	4.9/89.1	0.8	127	0.055	5.74	3.14
aANM	-	7.8/9.1	16.4/71.7	3.3	373	0.122	10.87	4.25
ANMPathway	-	-	-	-	-	-	-	-
MinActionPath2	-	8.5/7.6	14.8/68.8	2.3	260	0.175	11.18	3.99

Supplementary Table 13. MolProbity[24] assessment of RBP end-state and eBDIMS2 intermediate conformations. Experimental end states (1ba2, 2dri) and the eBDIMS2 intermediate along the closing pathway (1ba2-2dri) reconstructed via cg2all[23]. The quality of the eBDIMS2 intermediate is assessed: (i) right after all-atom reconstruction with cg2all; (ii) after 5,000 minimization steps with solvent; (iii) after 125 ps NPT equilibration; (iv) after 1ns of unbiased MD.

Conformer	cg2all + refinement	Ramachandran outlier/favored (%)	Rotamer outliers (%)	Clash-score	RMS bonds	RMS angles	MP score
1ba2	No cg2all<br>No refinement	0.4/98.5	5.2	1	0.007	1.94	1.05
2dri	No cg2all<br>No refinement	0.4/98.9	2.9	0	0.010	1.15	0.85
eBDIMS2 intermediate	cg2all<br>No refinement	0.7/95.9	1.5	125	0.024	2.02	2.98
	cg2all +<br>Minimization	0.4/95.5	1.5	4	0.017	2.20	1.61
	cg2all +<br>Minimization +<br>Equilibration	0.0/94.4	3.6	1	0.034	3.58	1.69
	cg2all +<br>Minimization +<br>Equilibration +<br>1ns MD	1.1/94.4	4.1	1	0.034	3.61	1.64

Supplementary Table 14. MolProbity[24] assessment of RNaseIII end-state and eBDIMS2 intermediate conformations. Experimental end states (1yyo, 1yyw) and the eBDIMS2 intermediate along the opening pathway (1yyo-1yyw) reconstructed via cg2all[23] and additional refinement steps.

Conformer	cg2all + refinement	Ramachandran outlier/favored (%)	Rotamer outliers (%)	Clash-score	RMS bonds	RMS angles	MP score
1yyo	No cg2all<br>No refinement	1.6/86.9	2.1	0	0.004	1.03	1.36
1yyw	No cg2all<br>No refinement	0.9/88.6	4.9	0	0.006	1.05	1.61
eBDIMS2 intermediate	cg2all<br>No refinement	3.3/88.1	2.8	205	0.028	2.65	3.71
	cg2all +<br>Minimization	3.0/89.7	3.4	0	0.017	2.33	1.46
	cg2all +<br>Minimization +<br>Equilibration	2.1/90.0	3.9	0	0.034	3.57	1.50
	cg2all +<br>Minimization +<br>Equilibration +<br>1ns MD	2.6/91.1	2.5	0	0.416	3.70	1.32

Supplementary Table 15. MolProbity[24] assessment of SERCA end-state and eBDIMS2 intermediate conformations. Experimental end states (2c9m, 1t5s) and the eBDIMS2 intermediate along the closing pathway (2c9m-1t5s) reconstructed via cg2all[23] and additional refinement steps.

Conformer	cg2all + refinement	Ramachandran outlier/favored (%)	Rotamer outliers (%)	Clash-score	RMS bonds	RMS angles	MP score
2c9m	No cg2all<br>No refinement	5.6/79.1	8.3	0	0.010	1.57	1.95
1t5s	No cg2all<br>No refinement	2.3/91.7	9.8	6	0.011	1.64	1.75
eBDIMS2 intermediate	cg2all<br>No refinement	4.0/86.0	2.3	207	0.032	3.07	3.70
	cg2all +<br>Minimization	4.3/84.3	5.3	5	0.017	2.44	2.51
	cg2all +<br>Minimization +<br>Equilibration	3.3/86.0	6.2	4	0.034	3.65	2.40
	cg2all +<br>Minimization +<br>Equilibration +<br>1ns MD	2.8/90.8	4.0	1	0.035	3.60	1.83

Supplementary Table 16. MolProbity[24] assessment of GroEL 7-mer end-state and eBDIMS2 intermediate conformations. Experimental end states (1ss8, 1sx4) and the eBDIMS2 intermediate along the opening pathway (1ss8-1sx4) reconstructed via cg2all[23] and additional refinement steps.

Conformer	cg2all + refinement	Ramachandran outlier/favored (%)	Rotamer outliers (%)	Clash-score	RMS bonds	RMS angles	MP score
1ss8	No cg2all<br>No refinement	0.4/96.8	15.0	0	0.027	2.14	1.59
1sx4	No cg2all<br>No refinement	1.1/91.1	18.2	0	0.025	2.14	1.97
eBDIMS2 intermediate	cg2all<br>No refinement	3.4/88.3	3.0	160	0.032	2.93	3.62
	cg2all +<br>Minimization	3.5/89.7	4.8	2	0.017	2.30	2.08
	cg2all +<br>Minimization +<br>Equilibration	2.7/91.6	5.6	2	0.034	3.63	2.09
	cg2all +<br>Minimization +<br>Equilibration +<br>1ns MD	1.5/93.3	3.1	1	0.034	3.59	1.60

Supplementary Table 17. System and simulation details for unbiased MD and TMD simulations.

Protein	Number of residues for MD (PCA)	Conformation	Number of atoms[†]	MD type	Production-run time (ns)
RBP	271 (271)	Open (1ba2)	66,166 (T)<br>4,070 (P)<br>61,980 (W)<br>116 (I)	Unbiased	3 x 200 ns
				TMD (target: 2dri)	3 x 1 ns
		Closed (2dri)	51,856 (T)<br>4,059 (P)<br>47,706 (W)<br>91 (I)	Unbiased	3 x 200 ns
				TMD (target: 1ba2)	3 x 1 ns
		Closing intermediate (eBDIMS2 midpoint in 1ba2-2dri pathway)[*]	55,755 (T)<br>4,072 (P)<br>51,585 (W)<br>98 (I)	Unbiased	3 x 200 ns
		Opening intermediate (eBDIMS2 midpoint in 2dri-1ba2 pathway)[*]	68,100 (T)<br>4,058 (P)<br>63,918 (W)<br>124 (I)	Unbiased	3 x 200 ns
RNaseIII	432 (432)	Closed (1yyo)	70,318 (T)<br>7,370 (P)<br>62,814 (W)<br>134 (I)	Unbiased	3 x 200 ns
				TMD (target: 1yyw)	3 x 1 ns
		Open (1yyw)	197,588 (T)<br>7,360 (P)<br>189,852 (W)<br>376 (I)	Unbiased	3 x 200 ns
				TMD (target: 1yyo)	3 x 1 ns
		Opening intermediate (eBDIMS2 midpoint in 1yyo-1yyw pathway)[*]	112,421 (T)<br>7,371 (P)<br>104,835 (W)<br>215 (I)	Unbiased	3 x 200 ns
SERCA	993 (993)	Open (2c9m)	386,047 (T)<br>15,433 (P)<br>369,888 (W)<br>726 (I)	Unbiased	3 x 200 ns
				TMD (target: 1t5s)	3 x 1 ns
		Closed (1t5s)	401,947 (T)<br>15,435 (P)<br>385,758 (W)<br>754 (I)	Unbiased	3 x 200 ns
				TMD (target: 2c9m)	3 x 1 ns
		Opening intermediate (eBDIMS2 midpoint in 1t5s-2c9m pathway)[*]	432,138 (T)<br>15,435 (P)<br>415,893 (W)<br>810 (I)	Unbiased	3 x 200 ns
hs_DNA-PKcs	4,118 (2,993)[+]	Inactive (7k19)	738,102 (T)<br>66,099 (P)<br>670,728 (W)<br>1,275 (I)	Unbiased	3 x 200 ns
				TMD (target: 7k0y)	3 x 2 ns
		Active (7k0y)	807,845 (T)<br>66,099 (P)<br>740,337 (W)<br>1,409 (I)	Unbiased	3 x 200 ns
				TMD (target: 7k19)	3 x 2 ns
hs_ACLY	4,384 (4,032)[+]	Apo (6pof)	949,448 (T)	Unbiased	3 x 200 ns

			67,840 (P)<br>879,948 (W)<br>1,660 (I)	TMD<br>(target: 6hxh)	3 x 2 ns
		Holo (6hxh)	888,395 (T)<br>67,836 (P)<br>819,009 (W)<br>1,550 (I)	Unbiased	3 x 200 ns
				TMD<br>(target: 6pof)	3 x 2 ns
SARS2_Spike<br>(from Amaro's lab[30,31])	3,774 (2,910)	Open (6vsb)	72,759 (T)	Unbiased	3 x ~ 1000 ns
				Adaptive sampling	3 x ~ 400 ns
		Closed (6vxx)		Unbiased	3 x ~ 550 ns
		Open mutant (6vsb + N165A + N234A)	71,294 (T)	Unbiased	3 x ~ 1000 ns
				Adaptive sampling	3 x ~ 400 ns
		Closed (model from Casalino et al.[30])	63,787 (T)	Weighted ensemble (WE) propagation[31]	1 x 175 ns

[*]All-atom reconstruction of CG eBDIMS2 intermediates was performed via cg2all[23]. [+]Missing residues were added via SWISS-MODEL[27]. [†]Number of atoms specifies the total (T) number of atoms in the solvated box, as well as the number of atoms related to the protein (P), water (W), and ion (I) systems; except for *SARS-CoV-2* spike, where these numbers only correspond to the total number of atoms for the proteins and glycans, since water molecules and ions are not provided.

Supplementary Table 18. Essential dynamics (ED) analysis of unbiased MD trajectories and overlaps with experimental PCs.

Protein	PDB	Trajectory	ED1 (%)	ED2 (%)	$RMSIP_3$	$RMSIP_5$	$RMSIP_{10}$	O_{max} (ED, PC mode)
RBP	1ba2	Replica 1	40.82	24.73	0.76	0.64	0.65	0.87 (1,1)
		Replica 2	46.24	21.64	0.77	0.67	0.65	0.92 (1,1)
		Replica 3	61.28	10.46	0.77	0.65	0.65	0.78 (1,1)
		All replicas	54.16	19.16	0.76	0.64	0.65	0.81 (1,1)
	2dri	Replica 1	61.74	21.67	0.75	0.61	0.66	0.84 (1,1)
		Replica 2	83.94	7.19	0.79	0.68	0.67	0.95 (1,1)
		Replica 3	62.19	15.68	0.73	0.64	0.67	0.95 (1,1)
		All replicas	72.23	13.75	0.77	0.63	0.67	0.93 (1,1)
	1ba2-2dri	Replica 1	44.11	19.83	0.77	0.65	0.65	0.93 (1,1)
		Replica 2	30.06	26.13	0.76	0.65	0.65	0.96 (2,1)
		Replica 3	36.78	29.37	0.77	0.65	0.65	0.87 (2,1)
		All replicas	36.85	26.58	0.78	0.65	0.65	0.94 (1,1)
	2dri-1ba2	Replica 1	51.76	22.97	0.68	0.65	0.64	0.75 (2,1)
		Replica 2	45.64	26.20	0.70	0.63	0.62	0.94 (2,1)
		Replica 3	43.00	23.04	0.77	0.64	0.63	0.97 (2,1)
		All replicas	40.96	23.17	0.64	0.63	0.64	0.91 (2,1)
RNaseIII	1yyo	Replica 1	66.91	13.48	0.60	0.67	0.65	0.62 (1,3)
		Replica 2	40.87	14.86	0.45	0.51	0.57	0.63 (1,3)
		Replica 3	47.07	23.37	0.54	0.59	0.61	0.68 (1,3)
		All replicas	72.63	12.50	0.65	0.67	0.64	0.60 (2,1)
	1yyw	Replica 1	41.15	19.94	0.57	0.59	0.57	0.55 (2,1)
		Replica 2	59.90	20.93	0.53	0.58	0.60	0.71 (3,1)
		Replica 3	42.08	17.84	0.48	0.62	0.60	0.55 (4,1)
		All replicas	43.40	19.50	0.48	0.60	0.60	0.58 (3,1)
	1yyo-1yyw	Replica 1	51.71	15.19	0.72	0.63	0.63	0.70 (1,1)
		Replica 2	45.64	23.79	0.47	0.58	0.64	0.51 (2,3)
		Replica 3	45.55	14.45	0.56	0.55	0.58	0.57 (5,6)
		All replicas	50.03	21.23	0.58	0.59	0.64	0.66 (1,1)
SERCA	2c9m	Replica 1	42.48	18.48	0.68	0.66	0.64	0.58 (2,2)
		Replica 2	70.02	15.64	0.63	0.65	0.65	0.80 (2,2)
		Replica 3	75.83	5.81	0.63	0.60	0.63	0.83 (1,2)
		All replicas	57.54	22.52	0.69	0.63	0.64	0.81 (1,2)
	1t5s	Replica 1	43.95	9.97	0.36	0.45	0.53	0.46 (4,5)
		Replica 2	47.35	10.39	0.20	0.38	0.48	0.55 (5,6)
		Replica 3	25.07	12.89	0.36	0.41	0.48	0.53 (2,6)
		All replicas	36.35	17.71	0.22	0.39	0.49	0.44 (5,5)
	1t5s-2c9m	Replica 1	47.49	12.25	0.37	0.51	0.58	0.69 (5,2)
		Replica 2	47.17	10.61	0.45	0.53	0.61	0.67 (2,2)
		Replica 3	32.64	18.46	0.52	0.54	0.60	0.51 (2,2)
		All replicas	35.24	23.99	0.48	0.51	0.58	0.59 (1,2)
hs_DNA-PKcs	7k19	Replica 1	34.04	14.80	0.47	0.47	0.53	0.45 (3,3)
		Replica 2	37.99	22.98	0.43	0.46	0.54	0.51 (6,1)
		Replica 3	36.48	13.03	0.53	0.54	0.55	0.58 (1,1)
		All replicas	55.57	14.16	0.44	0.51	0.55	0.40 (3,3)
	7k0y	Replica 1	53.30	9.55	0.22	0.24	0.40	0.32 (1,10)
		Replica 2	41.24	14.81	0.18	0.26	0.42	0.39 (10,9)
		Replica 3	50.52	7.50	0.18	0.26	0.44	0.37 (8,1)

		All replicas	29.99	22.86	0.18	0.24	0.38	0.36 (1,10)
hs_ACLY	6pof	Replica 1	39.18	22.10	0.58	0.68	0.67	0.76 (2,1)
		Replica 2	57.75	17.28	0.51	0.65	0.66	0.77 (1,1)
		Replica 3	48.15	16.72	0.45	0.60	0.62	0.55 (2,1)
		All replicas	55.37	25.11	0.50	0.60	0.68	0.68 (1,1)
	6hxh	Replica 1	43.67	14.60	0.65	0.63	0.68	0.75 (2,1)
		Replica 2	45.84	13.74	0.41	0.58	0.66	0.51 (5,5)
		Replica 3	55.67	16.28	0.58	0.69	0.67	0.80 (2,1)
		All replicas	55.24	12.63	0.50	0.60	0.71	0.58 (4,1)
SARS2_Spike[30]	6vsb	Replica 1	31.79	14.07	0.32	0.32	0.48	0.33 (5,6)
		Replica 2	30.85	11.99	0.18	0.36	0.41	0.33 (7,4)
		Replica 3	26.66	12.54	0.20	0.32	0.41	0.38 (3,4)
		All replicas	39.58	28.99	0.36	0.37	0.45	0.44 (7,4)
	6vsb (adaptive sampling)	Replica 1	23.28	11.41	0.15	0.28	0.40	0.41 (5,6)
		Replica 2	33.12	14.93	0.32	0.42	0.46	0.39 (2,4)
		Replica 3	26.85	10.68	0.15	0.39	0.42	0.40 (4,2)
		All replicas	41.84	34.59	0.40	0.43	0.46	0.38 (2,2)
	6vxx	Replica 1	26.25	13.30	0.23	0.29	0.41	0.36 (4,3)
		Replica 2	37.82	9.78	0.27	0.34	0.42	0.39 (10,10)
		Replica 3	34.22	11.97	0.25	0.26	0.38	0.28 (8,9)
		All replicas	30.26	26.94	0.27	0.32	0.40	0.25 (2,1)
	Mutant 6vsb	Replica 1	39.93	15.97	0.31	0.29	0.42	0.40 (9,5)
		Replica 2	39.41	12.98	0.43	0.46	0.48	0.54 (2,5)
		Replica 3	23.95	16.22	0.25	0.34	0.42	0.32 (6,5)
		All replicas	46.91	24.02	0.34	0.41	0.46	0.48 (4,5)
	Mutant 6vsb (adaptive sampling)	Replica 1	29.86	12.69	0.18	0.43	0.48	0.44 (4,1)
		Replica 2	44.45	9.02	0.42	0.41	0.49	0.48 (6,5)
		Replica 3	34.42	10.32	0.25	0.32	0.32	0.30 (8,10)
		All replicas	55.18	21.81	0.34	0.41	0.48	0.33 (3,5)

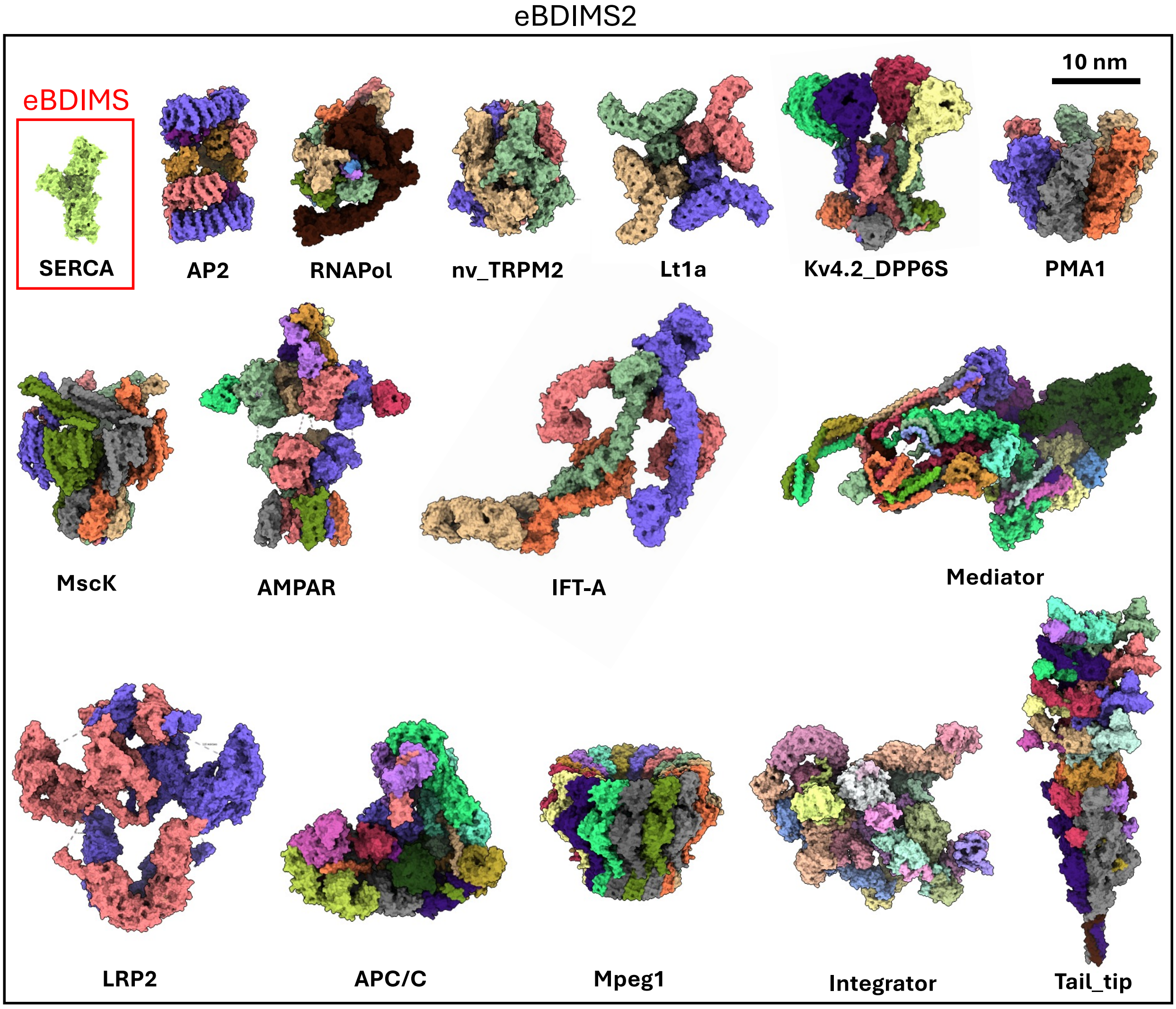

Supplementary Figure 1. Additional 15 large multimeric proteins (from ~350 kDa to ~1.1 MDa) with 2 experimental states available (more details in Supplementary Table 5). SERCA (~110 kDa) is also shown here for size comparison.

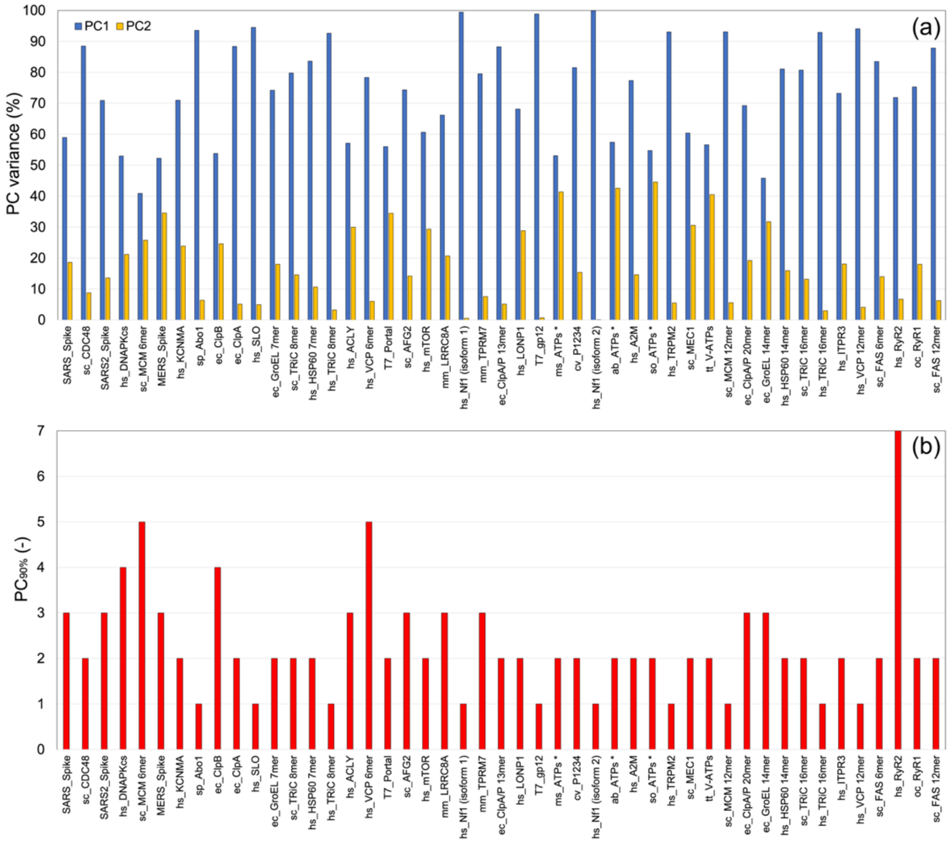

Supplementary Figure 2. PCA results of the 47 investigated experimental ensembles: **(a)** variance covered by the first 2 PCs; **(b)** number of PCs needed to describe at least 90% of the variance. All data presented in these plots are available in the Source Data file.

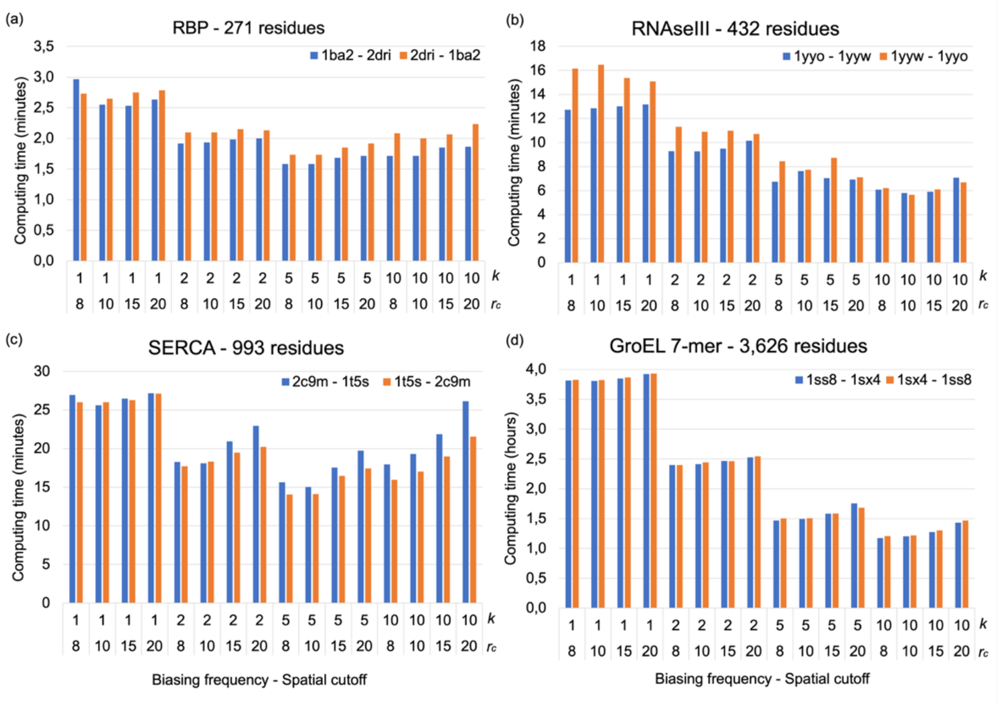

Supplementary Figure 3. Effect of the spatial cutoff r_c (8, 10, 15, 20 Å) and biasing frequency k (1, 2, 5, 10) on the computational efficiency of eBDIMS2: **(a)** RBP; **(b)** RNaseIII; **(c)** SERCA; **(d)** GroEL 7-mer. All computations have been performed on a Linux workstation with an Intel® Core i9-13900K processor and 64 GB of RAM. Values corresponding to r_c = 8 Å and k = 10 were selected as optimal and used for all subsequent calculations. All data presented in these plots are available in the Source Data file.

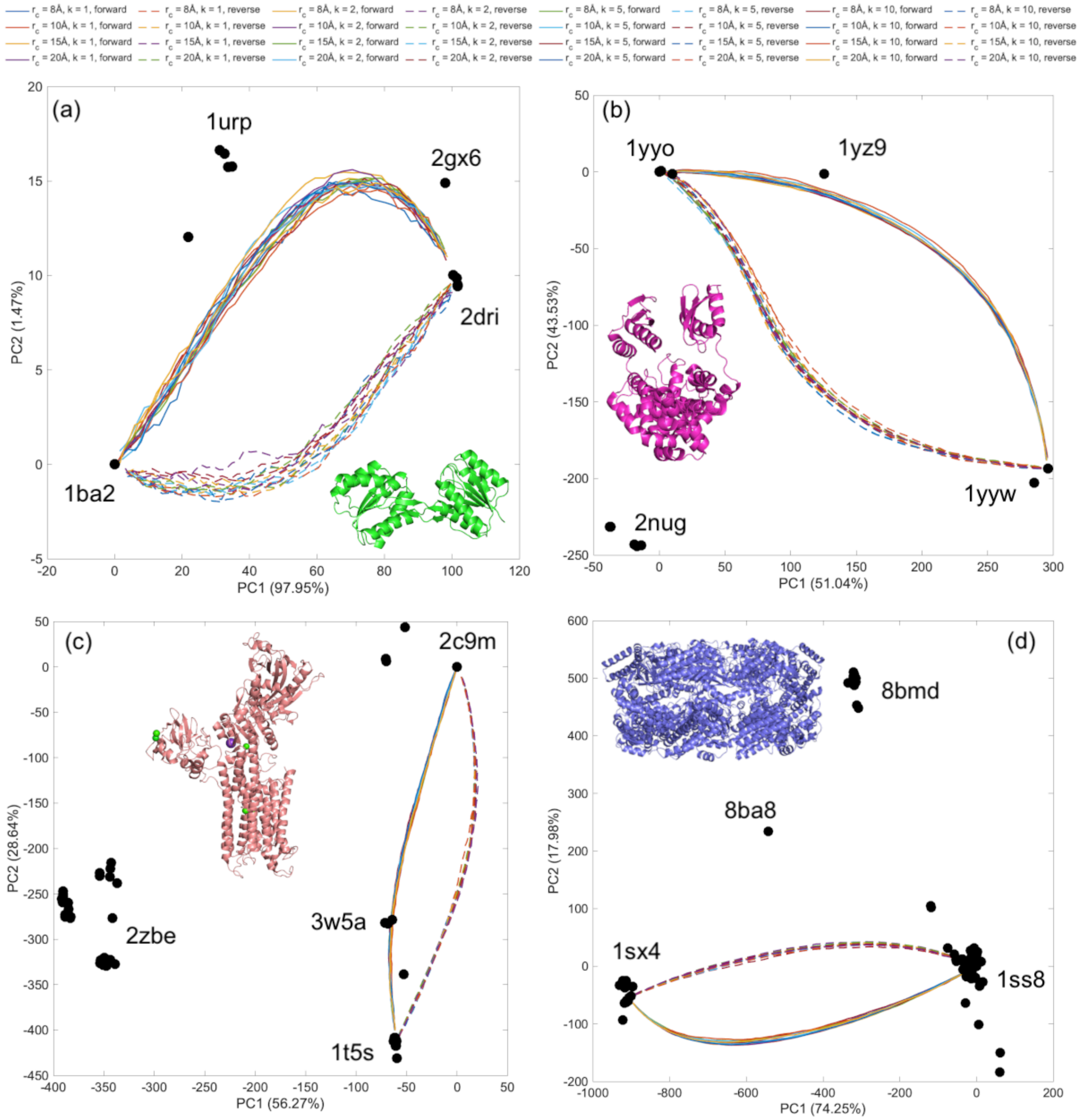

Supplementary Figure 4. Effect of the spatial cutoff r_c (8, 10, 15, 20 Å) and biasing frequency k (1, 2, 5, 10) on the accuracy of the eBDIMS2 pathways: **(a)** RBP; **(b)** RNaseIII; **(c)** SERCA; **(d)** GroEL 7-mer. No evident impact can be observed on the PC projections for different values of r_c or k.

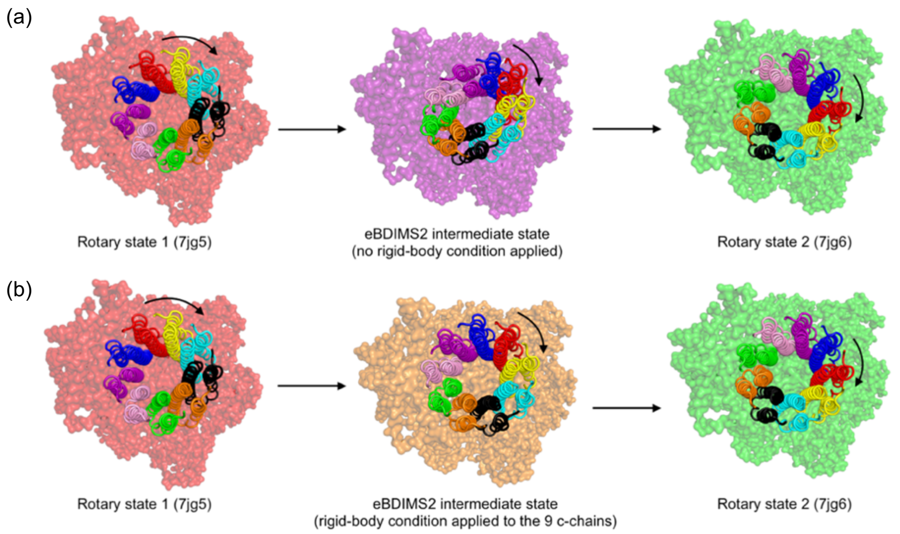

Supplementary Figure 5. Large-scale rigid rotational motions modelled in eBDIMS2: **(a)** artifact in the transition pathway of the F_0 rotor subunit of *M. Smegmatis* ATP synthase when standard ENM parameters are used for non-bonded interactions in the *c*-chains of the rotor; **(b)** more realistic pathway obtained by minimizing internal rotor deformations in eBDIMS2. All ATP synthase structures are viewed from the periplasm side. All structural components, except for the nine *c*-chains, are drawn with a semi-transparent surface representation, while the F_0 rotor subunits are shown as cartoons. Each *c*-chain is drawn in a different color to highlight the ~120° rotation from rotary state 1 to state 2.

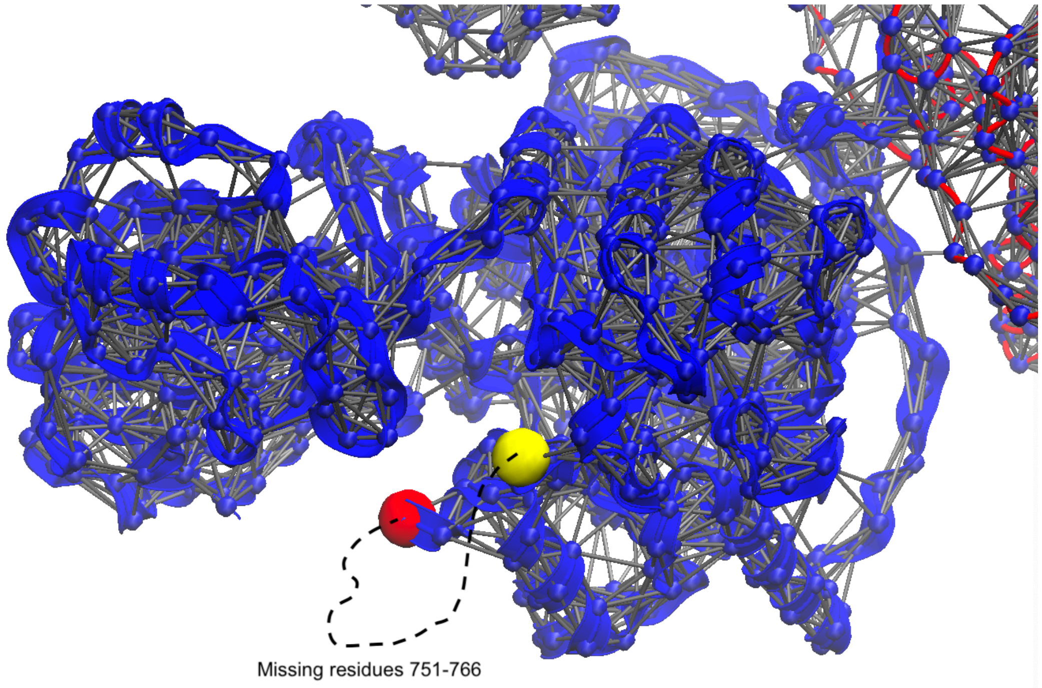

Supplementary Figure 6. Dealing with missing residues. Graphical representation of the edENM of ACLY (PDB: 6pof), highlighting a region with 16 missing residues (chain A, residues 751-766). Chain A is shown with a blue-ribbon representation, and thin gray cylinders are used to represent interacting springs. The yellow and red spheres correspond to residues 750 and 767, respectively, which are the last residues before the gap. The model ignores missing parts in the structure (usually disordered loops on the external surface) and uses non-bonded interactions to ensure the network connectivity between the other available residues.

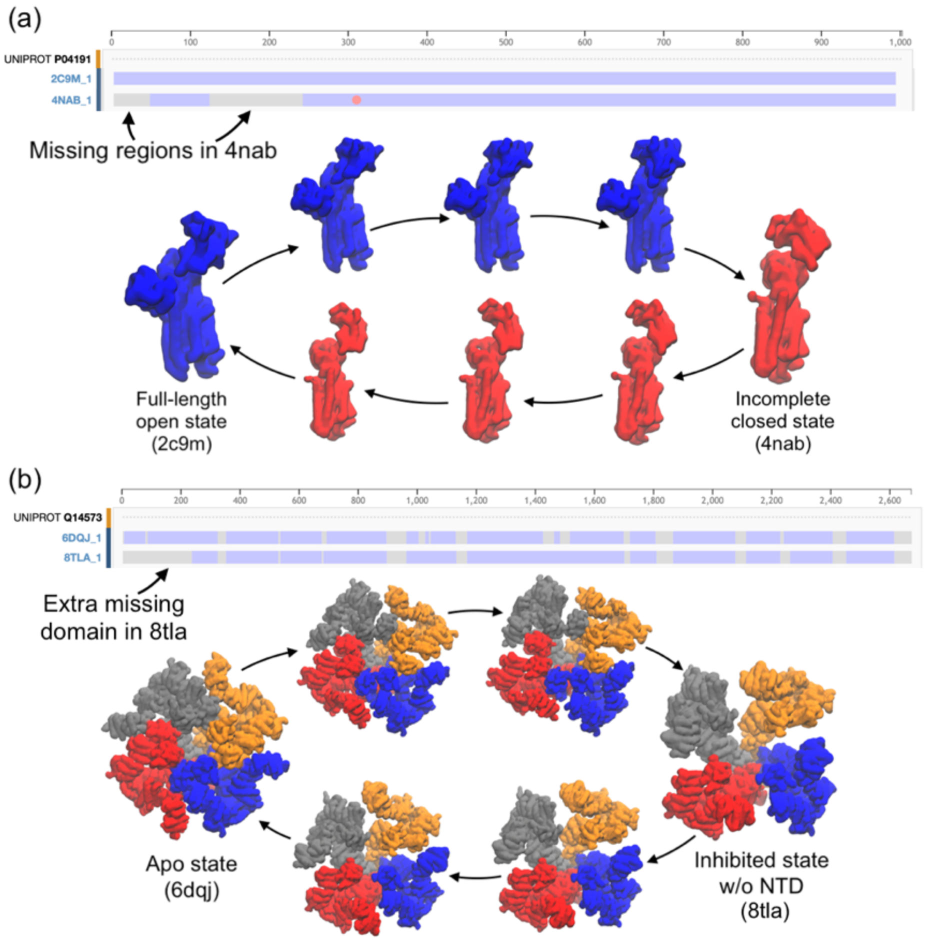

Supplementary Figure 7. Transition pathways between end-state conformers with different numbers of residues: **(a)** transition in SERCA between the full-length open state (PDB: 2c9m) and a closed state with most of the residues in the A-domain missing (4nab); **(b)** transition in ITPR3 between the apo state (6dqj) and an inhibited state with most of the residues in the N-terminal domain missing (8tla). Missing residues are highlighted in the sequences via the Sequence Alignment PDB webpage image: violet regions correspond to residues available in the 3D model, while grey ones account for missing residues.

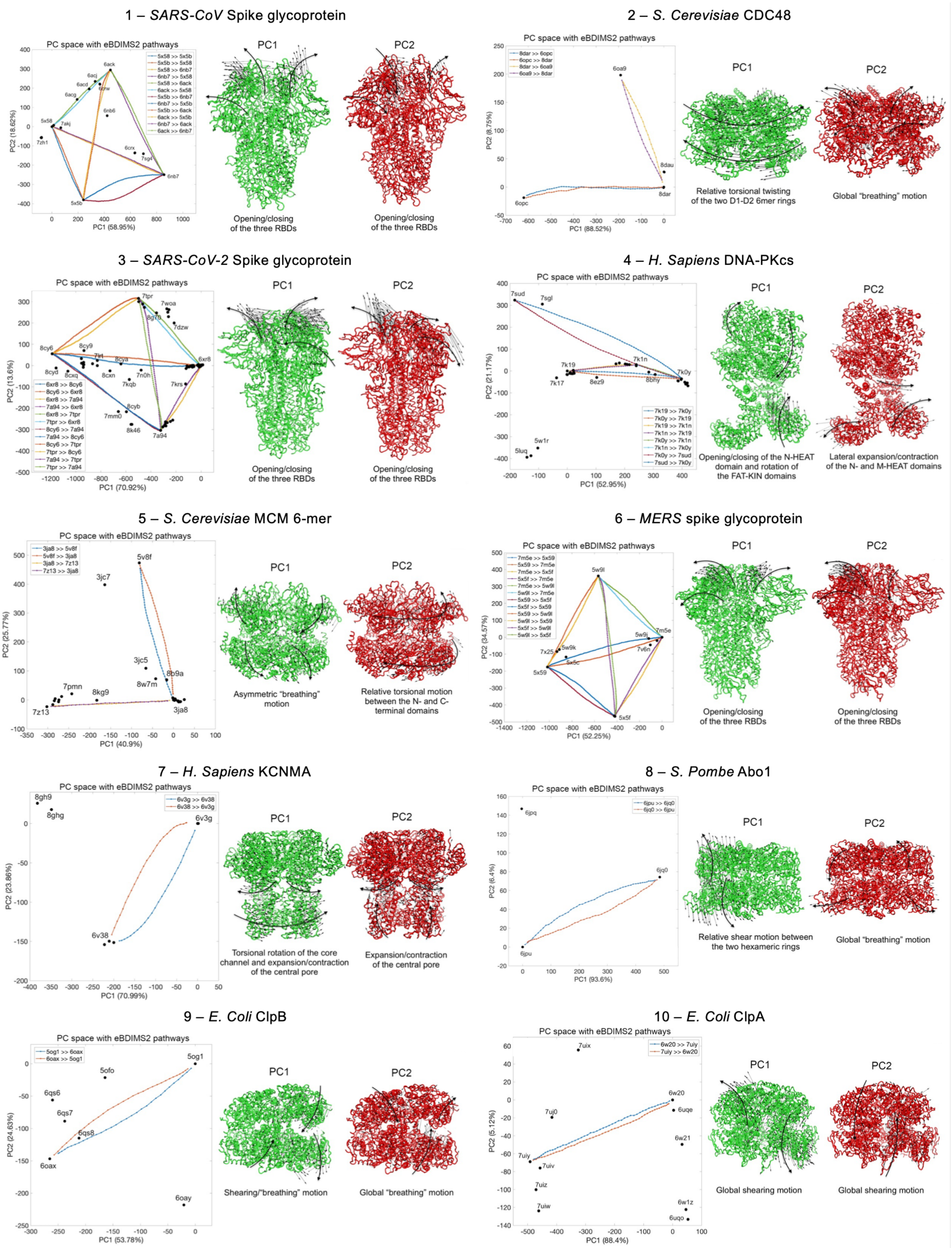

Supplementary Figure 8. PC space and eBDIMS2 transitions for: **(1)** *SARS-CoV* spike glycoprotein; **(2)** *S. Cerevisiae* CDC48; **(3)** *SARS-CoV-2* spike glycoprotein; **(4)** *H. Sapiens* DNA-PKcs; **(5)** *S. Cerevisiae* MCM 6-mer; **(6)** *MERS* spike glycoprotein; **(7)** *H. Sapiens* KCNMA; **(8)** *S. Pombe* Abo1; **(9)** *E. Coli* ClpB; **(10)** *E. Coli* ClpA. The PC1-2 spaces report experimental structures (black dots) and eBDIMS2 pathways (colored lines). Graphical representations of the apparent motions associated with PC1 (green) and PC2 (red) eigenvectors are also reported.

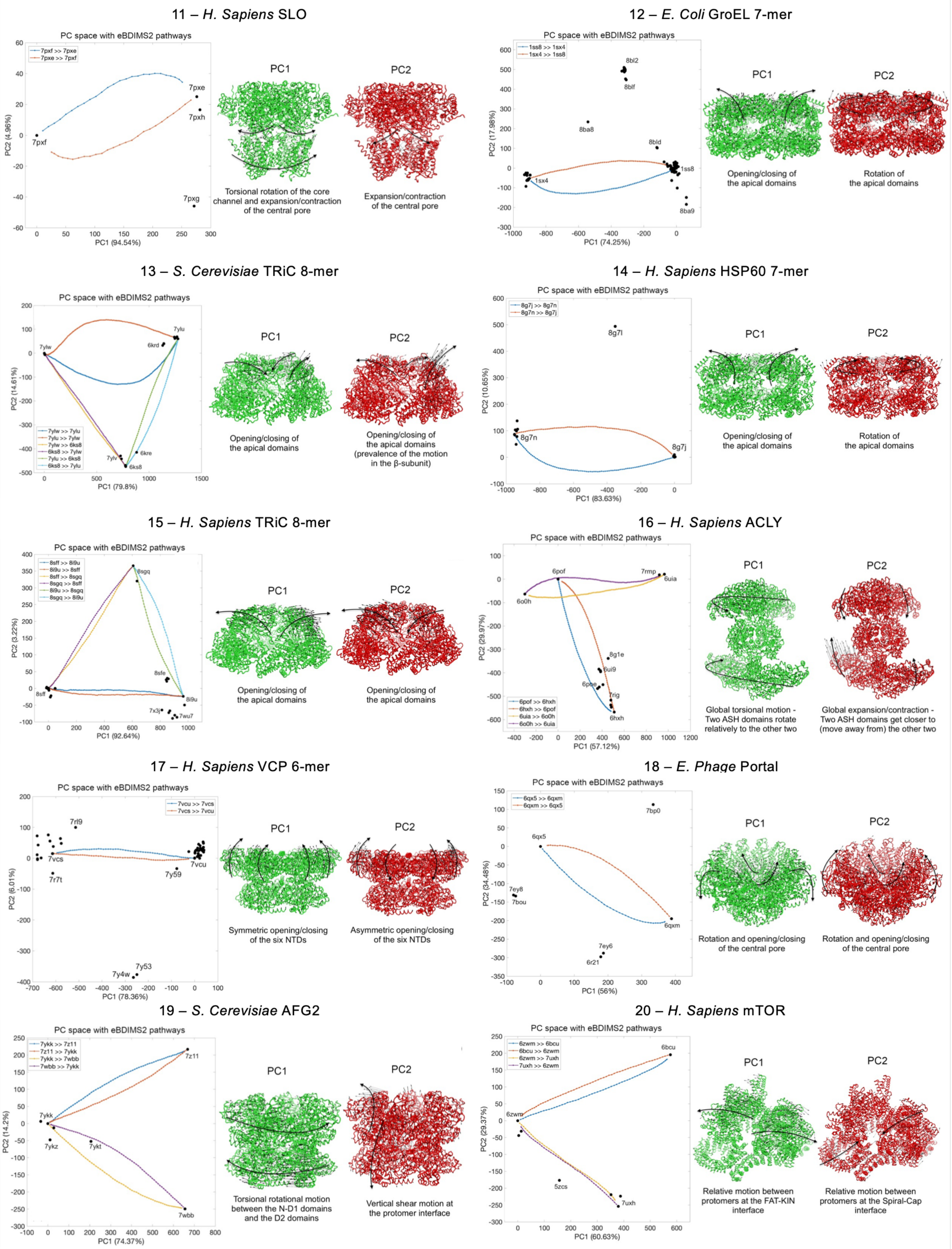

Supplementary Figure 9. PC space and eBDIMS2 transitions for: **(11)** *H. Sapiens* SLO; **(12)** *E. Coli* GroEL 7-mer; **(13)** *S. Cerevisiae* TRiC 8-mer; **(14)** *H. Sapiens* HSP60 7-mer; **(15)** *H. Sapiens* TRiC 8-mer; **(16)** *H. Sapiens* ACLY; **(17)** *H. Sapiens* VCP 6-mer; **(18)** *E. Phage* Portal; **(19)** *S. Cerevisiae* AFG2; **(20)** *H. Sapiens* mTOR.

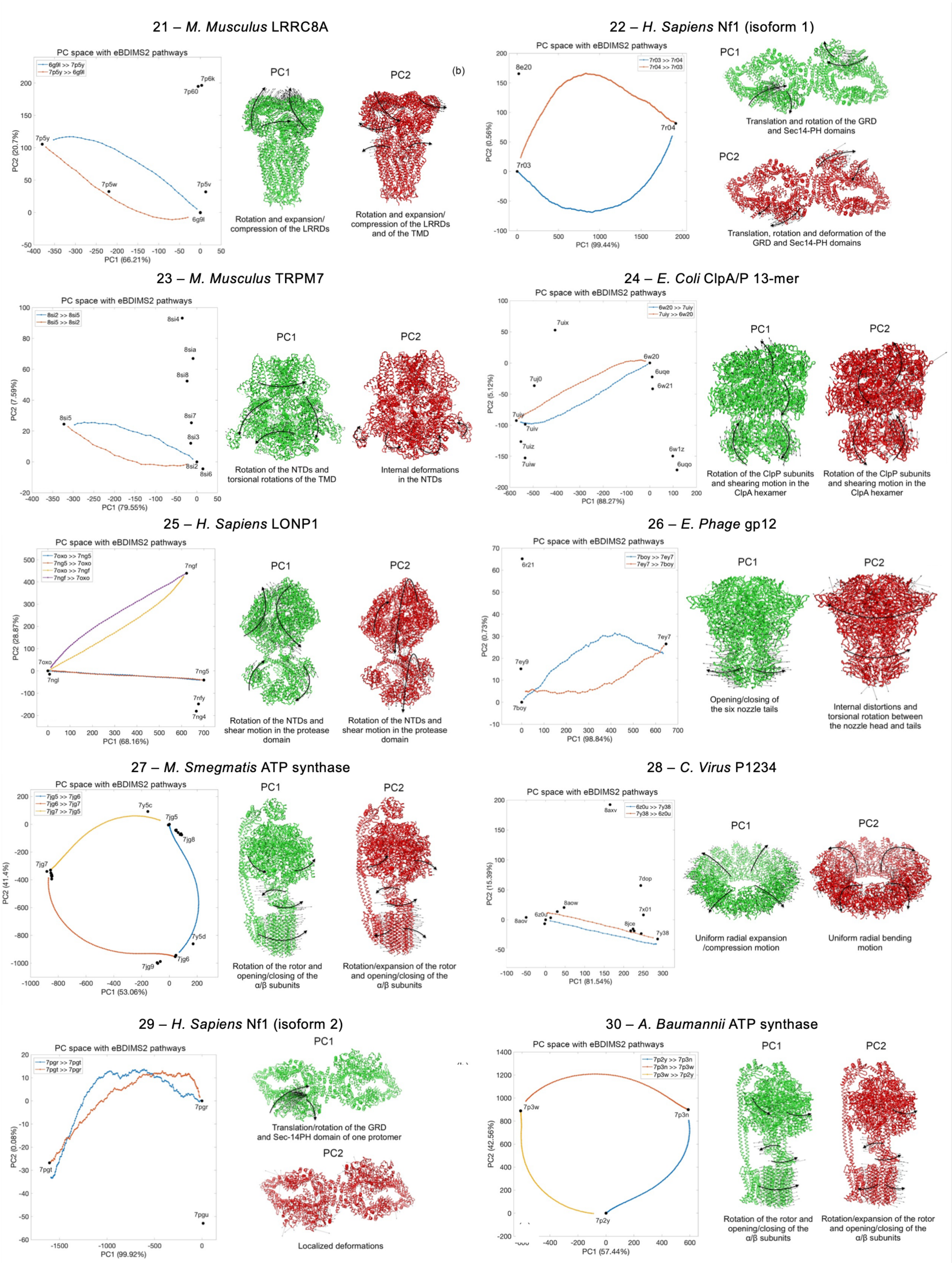

Supplementary Figure 10. PC space and eBDIMS2 transitions for: **(21)** *M. Musculus* LRRC8A; **(22)** *H. Sapiens* Nf1 (isoform 1); **(23)** *M. Musculus* TRPM7; **(24)** *E. Coli* ClpA/P 13-mer; **(25)** *H. Sapiens* LONP1; **(26)** *E. Phage* gp12; **(27)** *M. Smegmatis* ATP synthase; **(28)** *C. Virus* P1234; **(29)** *H. Sapiens* Nf1 (isoform 2); **(30)** *A. Baumannii* ATP synthase.

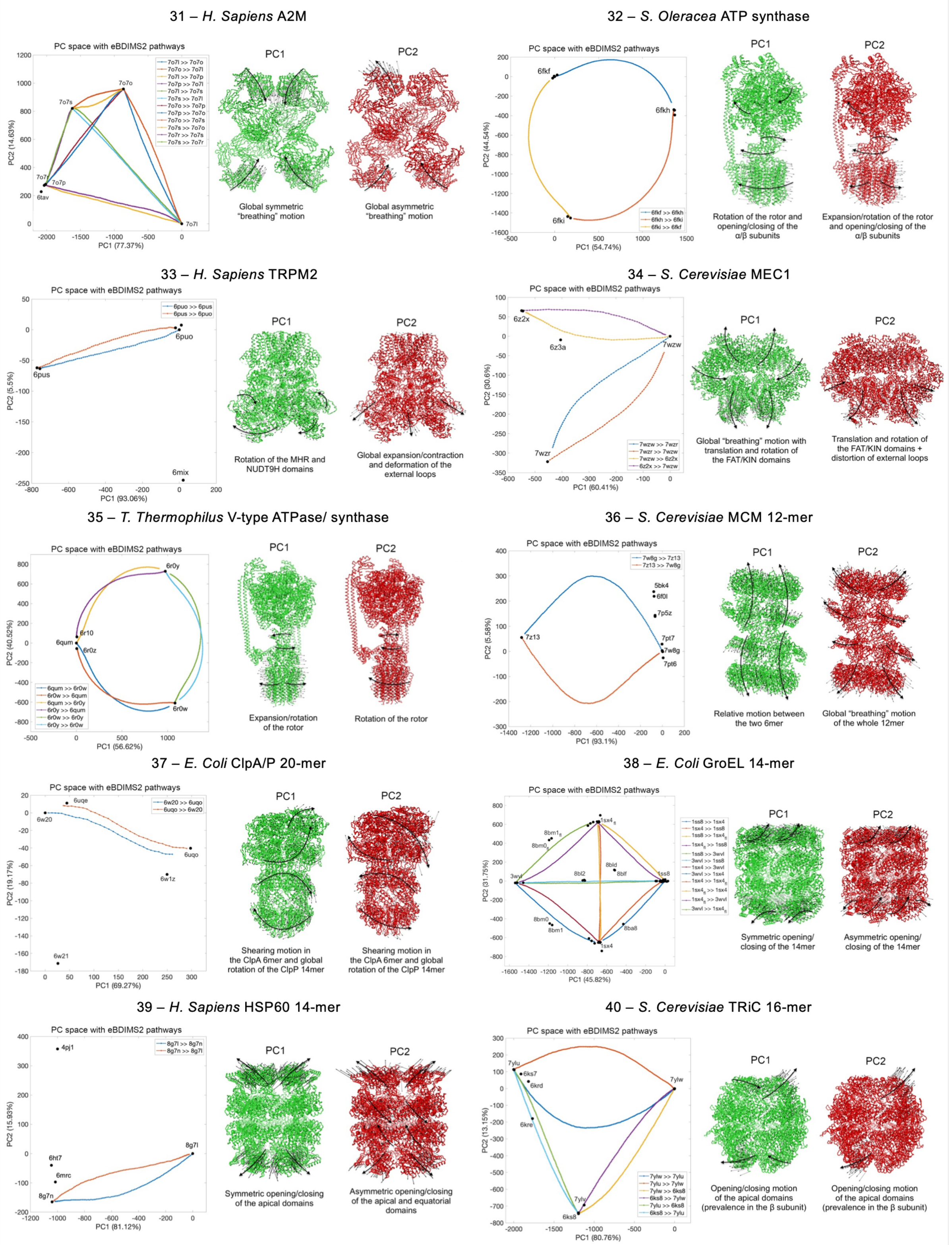

Supplementary Figure 11. PC space and eBDIMS2 transitions for: **(31)** *H. Sapiens* A2M; **(32)** *S. Oleracea* ATP synthase; **(33)** *H. Sapiens* TRPM2; **(34)** *S. Cerevisiae* MEC1; **(35)** *T. Thermophilus* V-type ATPase/synthase; **(36)** *S. Cerevisiae* MCM 12-mer; **(37)** *E. Coli* ClpA/P 20-mer; **(38)** *E. Coli* GroEL 14-mer; **(39)** *H. Sapiens* HSP60 14-mer; **(40)** *S. Cerevisiae* TRiC 16-mer.

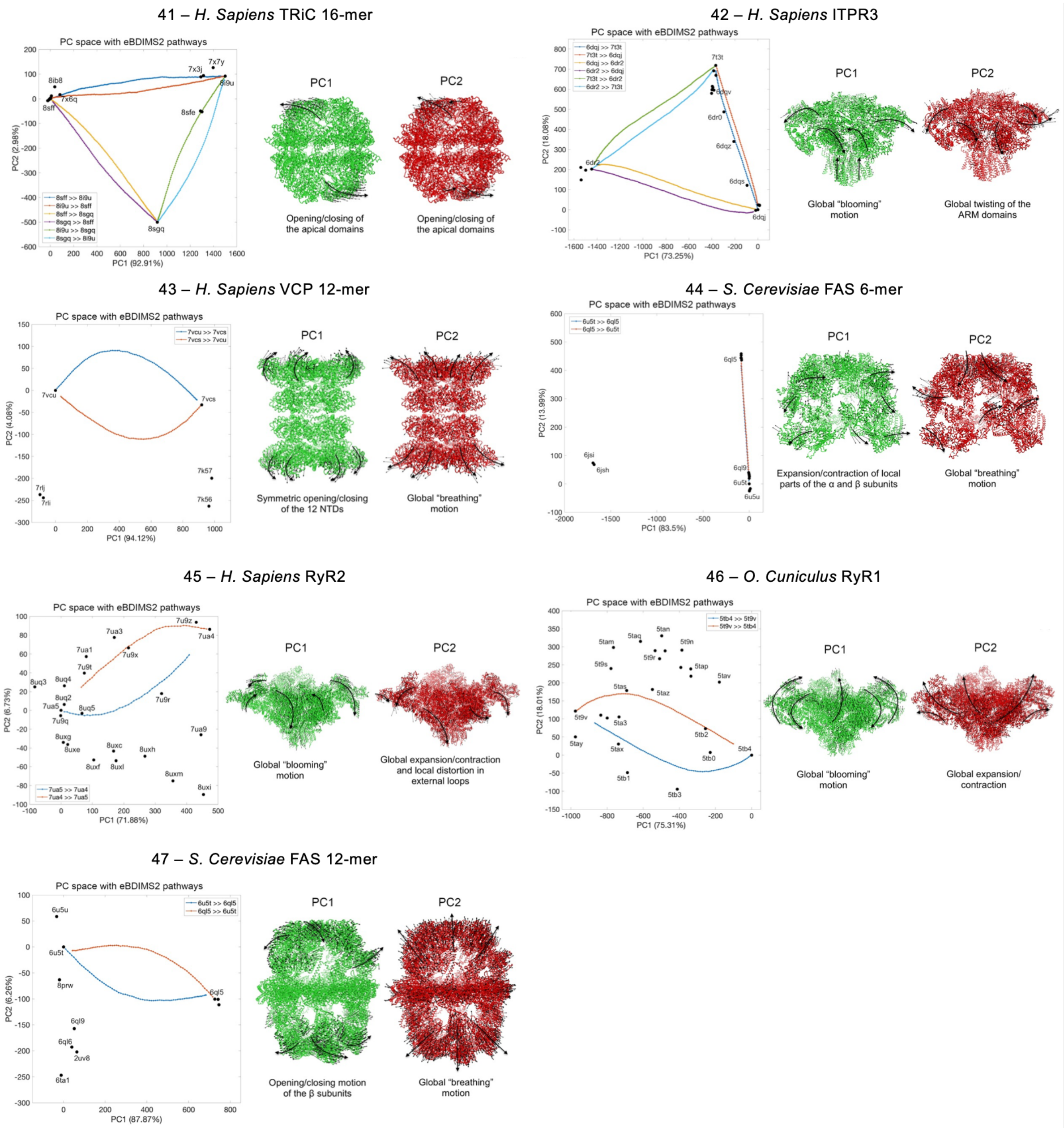

Supplementary Figure 12. PC space and eBDIMS2 transitions for: **(41)** *H. Sapiens* TRiC 16-mer; **(42)** *H. Sapiens* ITPR3; **(43)** *H. Sapiens* VCP 12-mer; **(44)** *S. Cerevisiae* FAS 6-mer; **(45)** *H. Sapiens* RyR2; **(46)** *M. Musculus* RyR1; **(47)** *S. Cerevisiae* FAS 12-mer.

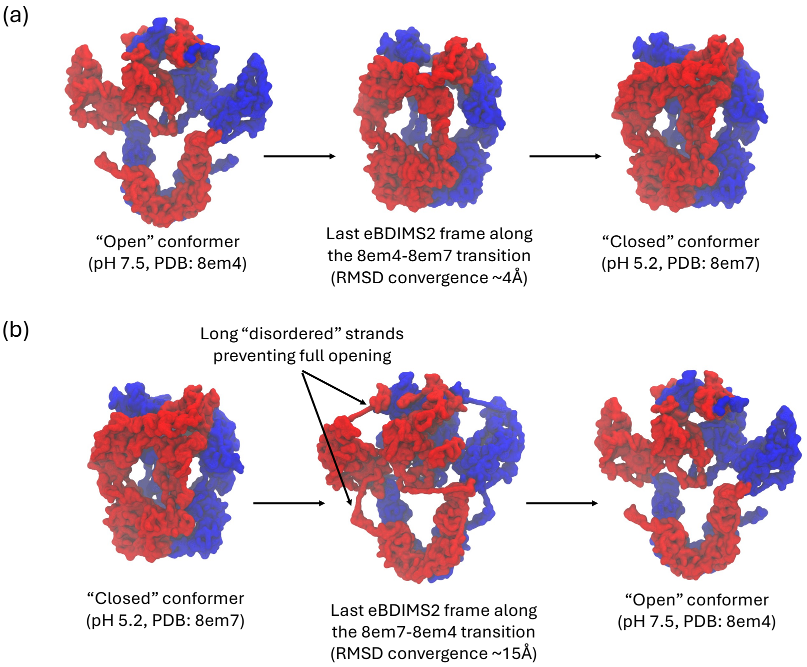

Supplementary Figure 13. Gigantic (RMSD ~57Å) transition in LRP2: **(a)** the eBDIMS2 transition from the open state at pH 7.5 to the more closed one at pH 5.2 can achieve good convergence at ~4Å from the target; **(a)** in the opposite direction, eBDIMS2 struggles to reach the target (convergence at ~15Å) due to the presence of long disordered strands that hinder full opening of the moving domains, which are only present in the cryo-EM model at pH 5.2.

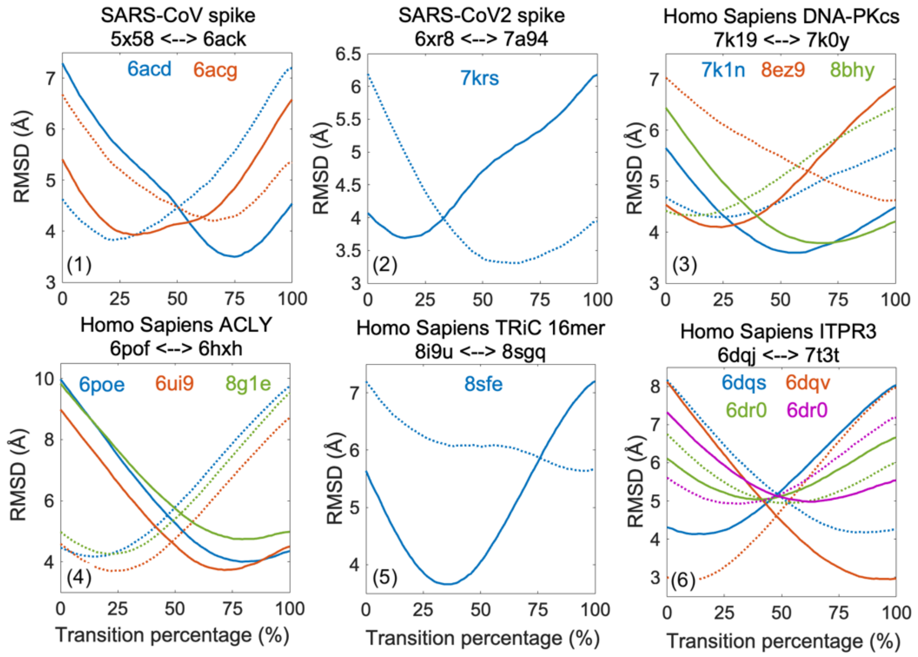

Supplementary Figure 14. RMSD values of eBDIMS2 pathways from on-path experimental intermediates for six selected systems: **(1)** *SARS-CoV* spike glycoprotein, transition between fully closed trimer (5x58) to one-RBP-up state (6ack); **(2)** *SARS-CoV-2* spike glycoprotein, transition between fully closed trimer (6xr8) to the one-RBP-up state (7a94); **(3)** DNA-PKcs, transition from apo-like inactive state (7k19) to active conformer (7k0y); **(4)** ACLY, transition from the apo state (6pof) to the conformation in complex with citrate-CoA-Mg-ADP (6hxh); **(5)** *H. Sapiens* TRiC 16-mer, transition between PhLP2A-ADP-bound open conformation (8i9u) to ADP-bound open state (8sgq); **(6)** ITPR3, transition between apo conformation (6dqj) to IP3-, ATP-, and Ca2+-bound active state (7t3t). Experimental intermediates that were used to compute RMSD are reported with different colors. Continuous and dashed lines refer to the distance between forward and reverse pathways, respectively.

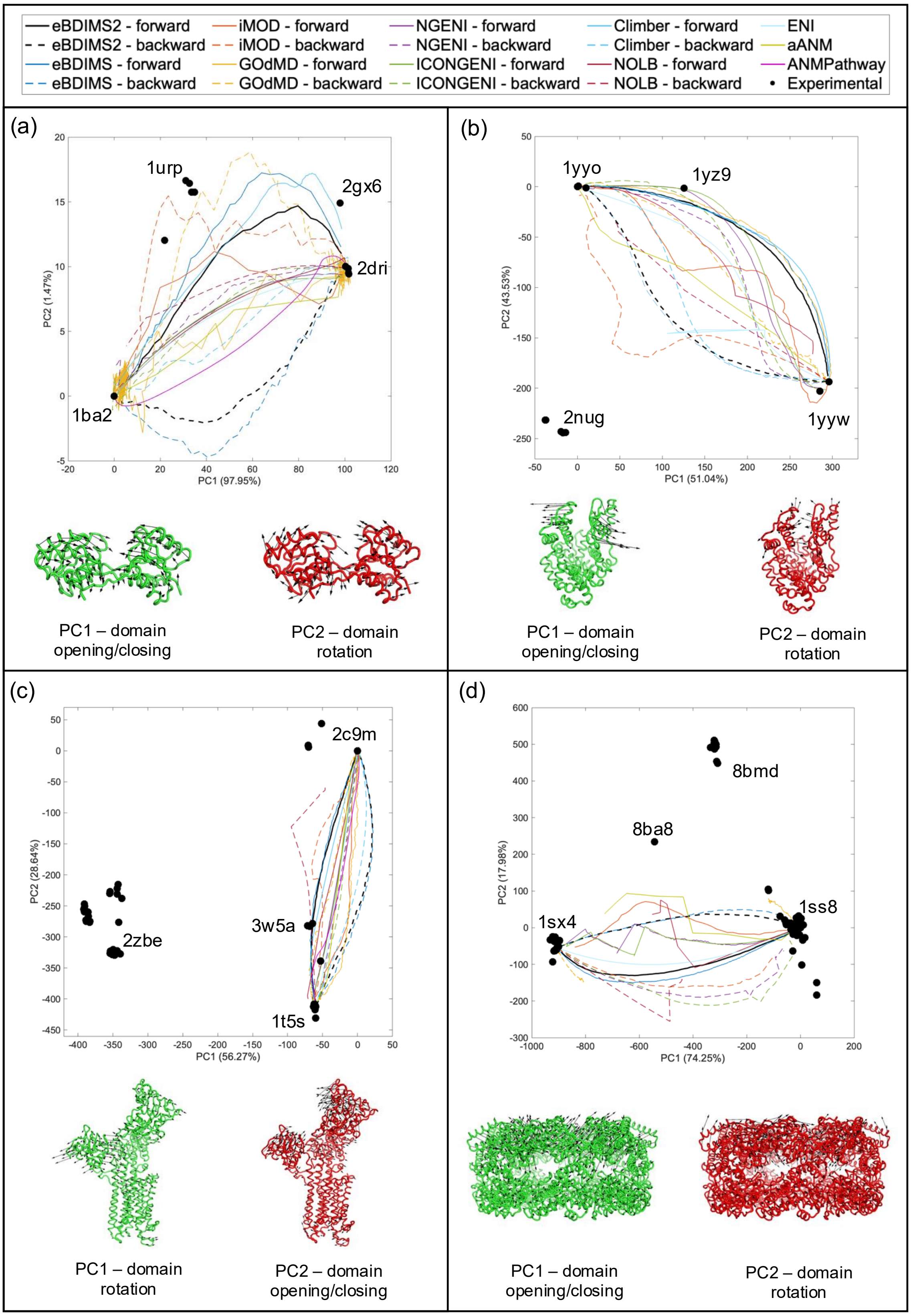

Supplementary Figure 15. PC results and transition pathways projections for: **(a)** RBP; **(b)** RNaseIII; **(c)** SERCA; **(d)** GroEL 7-mer. In the PC spaces, black dots indicate the experimental conformations in the ensemble, while colored lines represent the transition pathways evaluated with eBDIMS2, eBDIMS, iMOD, GOdMD, NGENI, ICONGENI, Climber, NOLB, ENI, aANM, and ANMPathway. Non-linear methods show two distinct pathways for the forward (continuous lines) and backward (dashed lines) directions, while linear methods predict one single pathway. Vector representations of the PC1 and PC2 essential motions are also shown for each protein.

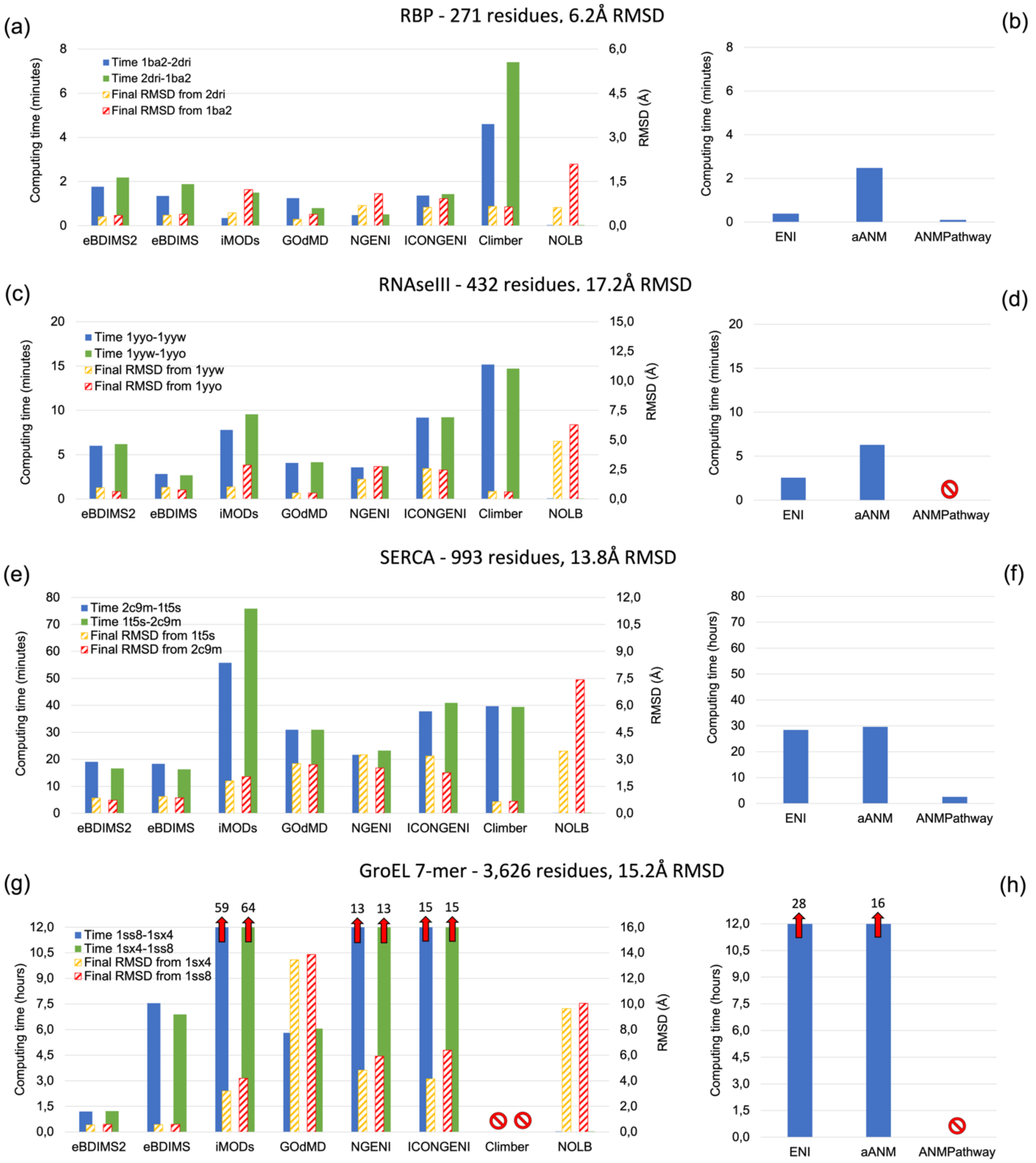

Supplementary Figure 16. Computing times for the conformational transitions of: **(a,b)** RBP; **(c,d)** RNaseIII; **(e,f)** SERCA; and **(g,h)** GroEL 7-mer. Panels (a,c,e,g) report the computing times for non-linear algorithms, for both the forward and backward directions, together with the value of RMSD from the target state at the moment of convergence. Panels (b,d,f,h) report the computing times for linear algorithms, where a single morphing transition connects the end states. Red stop signals in panels (d,g,h) indicate simulations that were aborted or not initiated by the code (see Supplementary Information text). Red arrows for iMODs, NGENI, ICONGENI, ENI, and aANM for GroEL point to the actual computing times (in hours) required for the simulation to terminate. All data presented in these plots are available in the Source Data file.

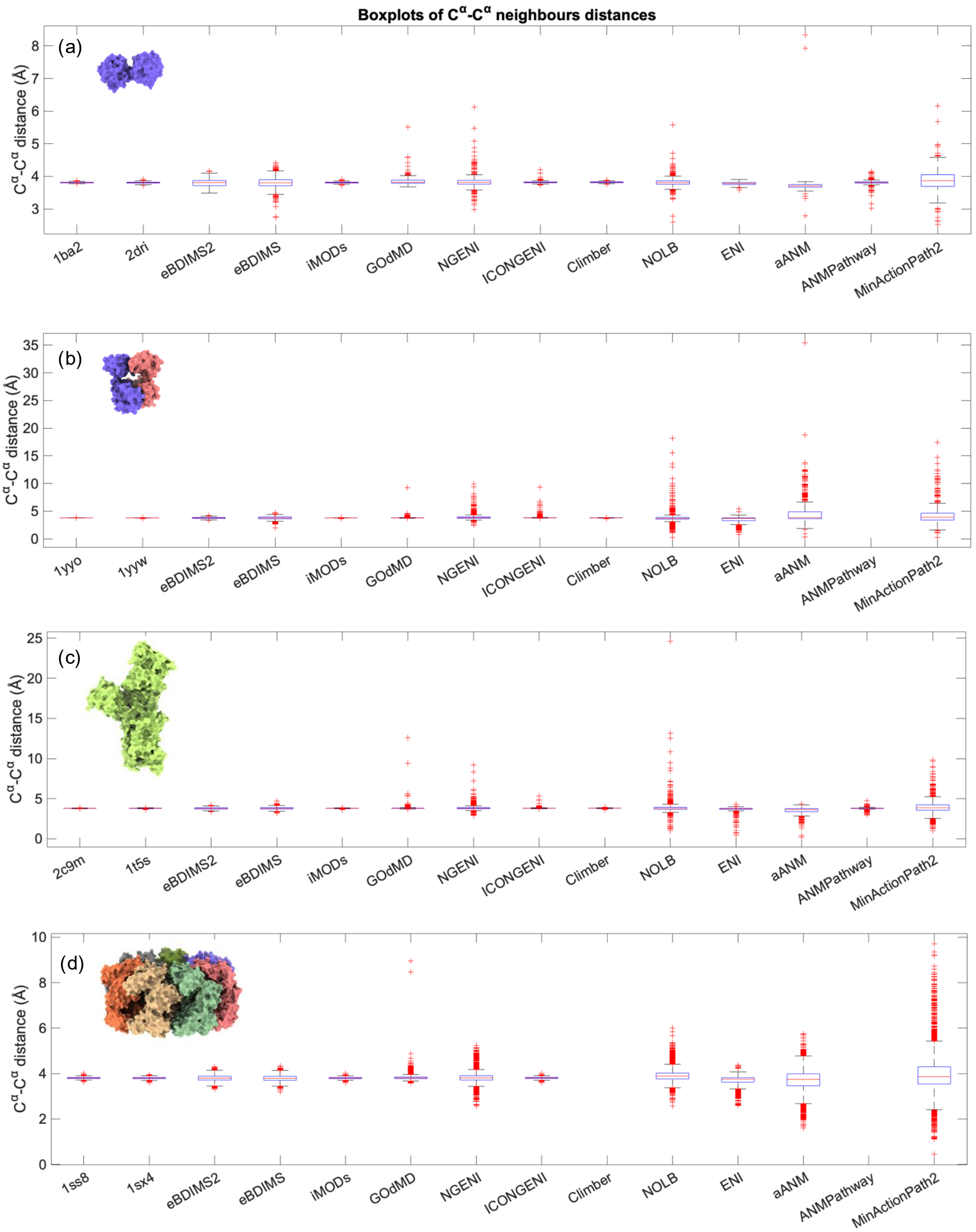

Supplementary Figure 17. Comparison of distributions of the distances between consecutive C^{α}-C^{α} atoms for the four benchmark (full-length) proteins: **(a)** RBP; **(b)** RNaseIII; **(c)** SERCA; **(d)** GroEL 7-mer. Distances between consecutive C^{α} atoms are reported using boxplot representations, considering n = 270 consecutive C^{α}-C^{α} distance for RBP, n = 430 for RNaseIII, n = 992 for SERCA, and n = 3,619 for GroEL 7-mer. The bottom and top of each box are the 25th and 75th percentiles of the distance distributions, and the middle red line represents the median value. Whiskers extend up to minimum and maximum values, while observations beyond the whiskers (red "+" signs) are considered outliers. The C^{α}-C^{α} distances in experimental PDB end states are compared to those in the mid-point intermediates generated by the path-sampling methods investigated above: eBDIMS2, eBDIMS, iMOD, GOdMD, NGENI, ICONGENI, Climber, NOLB, ENI, aANM, ANMPathway. Transition points generated via the MinActionPath2 webserver have also been included here for comparison. For non-linear methods both forward and backward intermediates have been considered. No data are reported for RNaseIII and GroEL for ANMPathway, as well as for GroEL for Climber, as no intermediate conformations were generated by these methods. All data presented in these plots are available in the Source Data file.

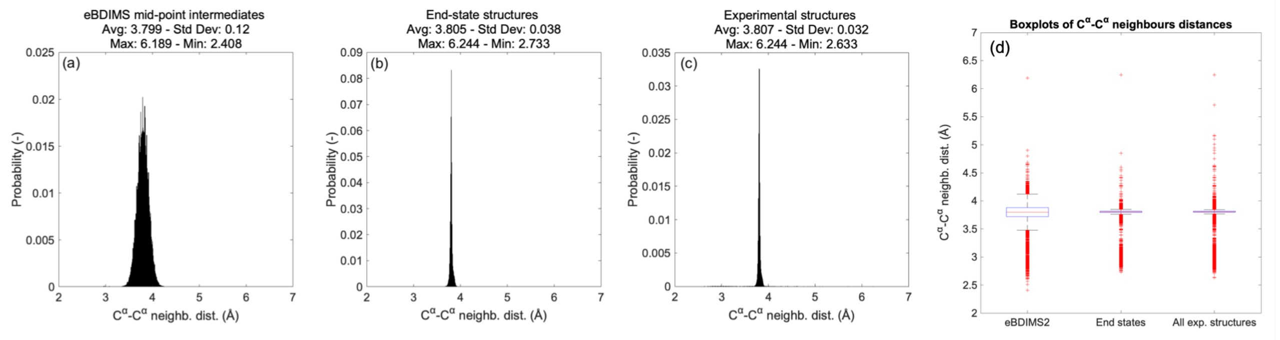

Supplementary Figure 18. Comparison between distribution of distances between consecutive C$^{\alpha}$-C$^{\alpha}$ atoms for the dataset of large protein ensembles: **(a)** mid-point intermediates along all 191 eBDIMS2 transition pathways; **(b)** the 124 end-state experimental conformers used to simulate transition pathways; and **(c)** all 872 experimental structures used to build structural ensembles. These plots have been produced considering a total of n = 967,464 consecutive C$^{\alpha}$-C$^{\alpha}$ distances for eBDIMS2 mid-point intermediates, n = 651,380 for end-state structures, and n = 4,608,450 for all experimental structures.

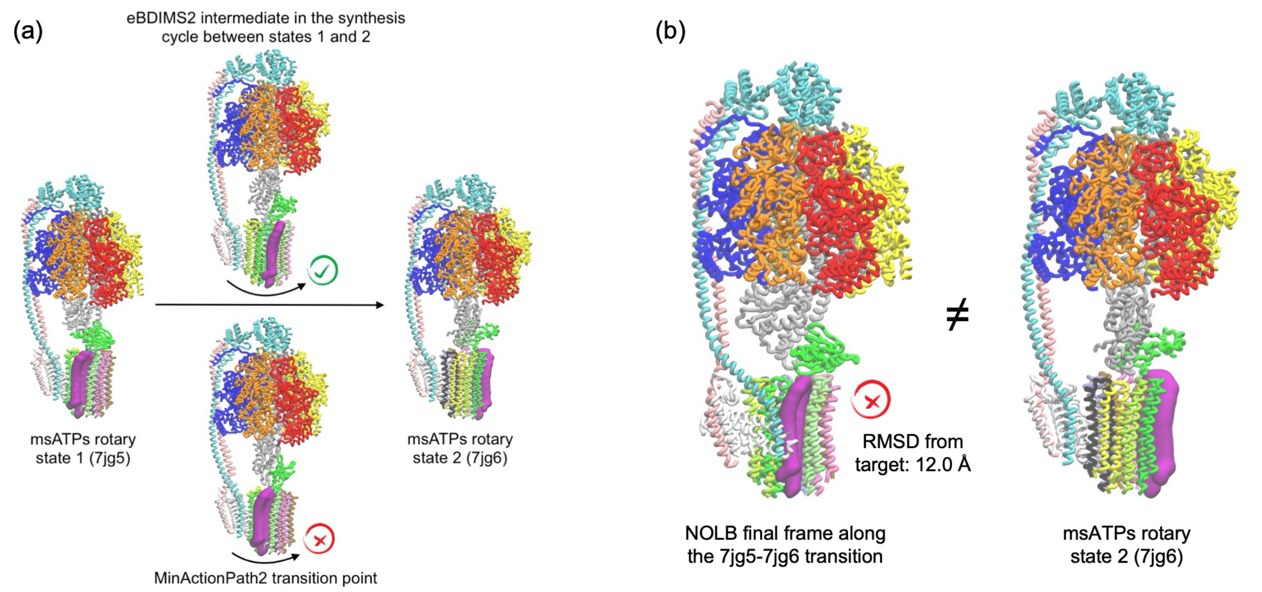

Supplementary Figure 19. Transition pathway for *M. Smegmatis* ATP synthase from rotary state 1 (7jg5) to rotary state 2 (7jg6) computed with eBDIMS2, MinActionPath2, and NOLB: **(a)** ribbon representation of the two end states, the eBDIMS2 intermediate (above), and the MinActionPath2 transition point (below). One *c*-chain of the rotor domain has been represented with a a purple surface to highlight that the MinActionPath2 transition does not generate any rotation of the F_0 rotor; **(b)** comparison between the last frame of the NOLB transition and the target conformation, highlighting the unsuccessful convergence of the NOLB trajectory (RMSD ~12 Å from the target).

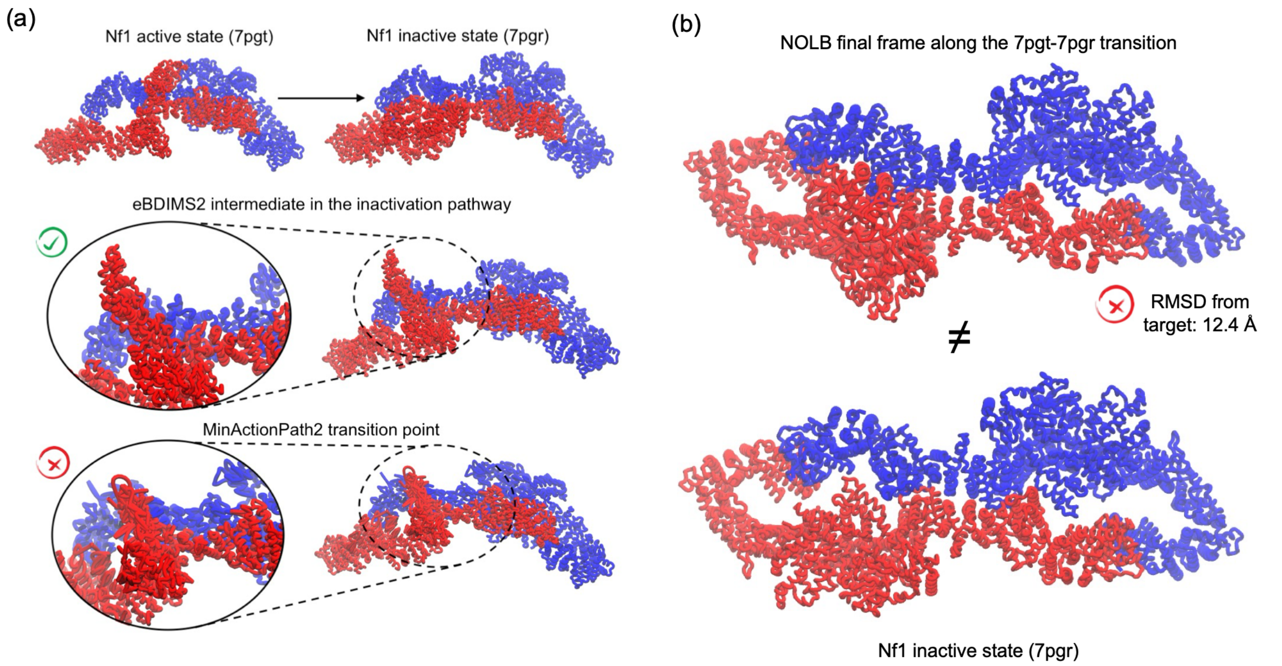

Supplementary Figure 20. Transition pathway for *H. Sapiens* Nf1 isoform 2 from active state (7pgt) to inactive state (7pgr) computed with eBDIMS2 and the MinActionPath2 webserver: **(a)** ribbon representation of the two end states, the eBDIMS2 intermediate, and the MinActionPath2 transition point. Zoomed views of the moving GRD domains to highlight the large internal distortions in the MinActionPath2 intermediate; **(b)** comparison between the last frame of the NOLB transition and the target conformation, highlighting the unsuccessful convergence of the NOLB trajectory (RMSD ~12 Å from the target).

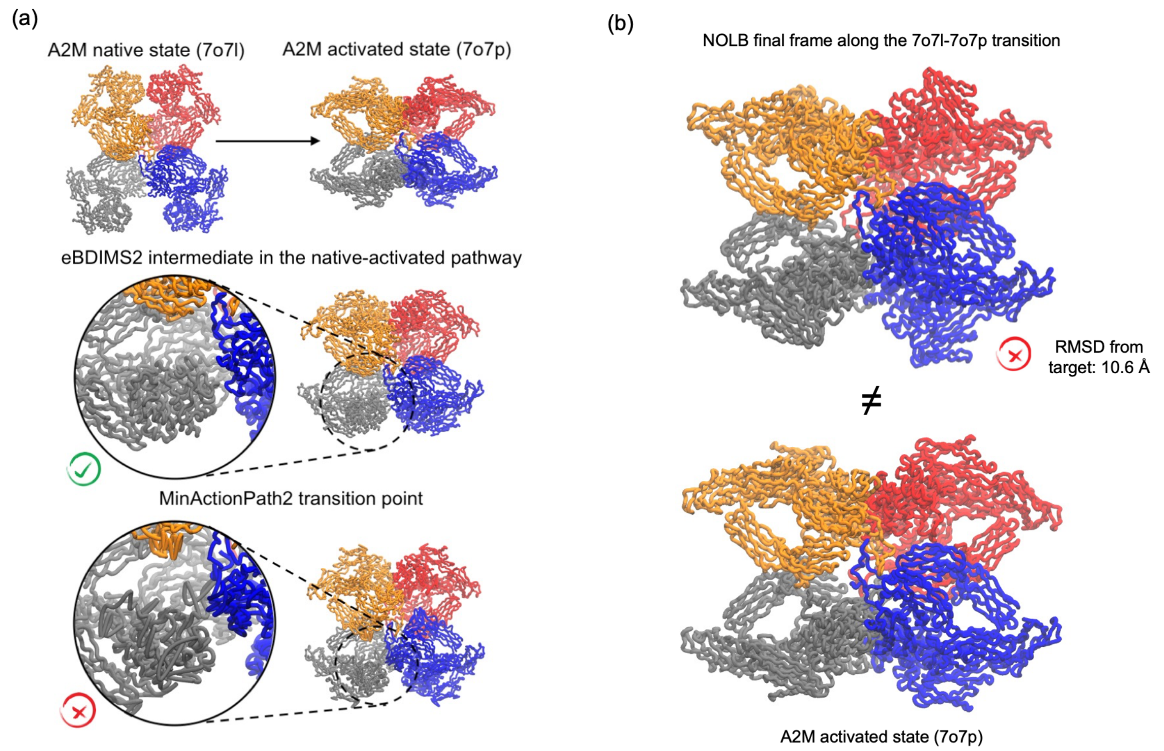

Supplementary Figure 21. Transition pathway for *H. Sapiens* A2M from native state (7o7l) to activated state (7o7p) computed with eBDIMS2 and the MinActionPath2 webserver: **(a)** ribbon representation of the two end states, the eBDIMS2 intermediate, and the MinActionPath2 transition point. Zoomed views of one of the A2M moving domains to highlight the major distortions in the MinActionPath2 transition point; **(b)** comparison between the last frame of the NOLB transition and the target conformation, highlighting the unsuccessful convergence of the NOLB trajectory (RMSD ~11 Å from the target).

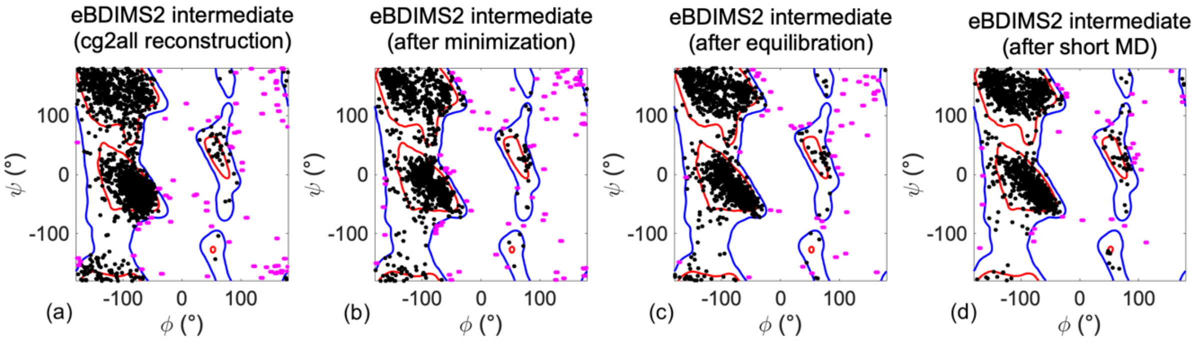

Supplementary Figure 22. Quality assessment of GroEL 7-mer eBDIMS2 intermediate along the opening pathway (1ss8-1sx4) based on the all-atom reconstruction performed with cg2all[23]: **(a)** without additional refinement; **(b)** after 5,000 steps of minimization with solvent; **(c)** after additional 125 ps of NPT equilibration; **(d)** after a short 1-ns unbiased MD. Ramachandran ϕ-ψ plots for the general amino acids, assessed via MolProbity[24]. Additional details on other MolProbity metrics are reported in Supplementary Table 16.

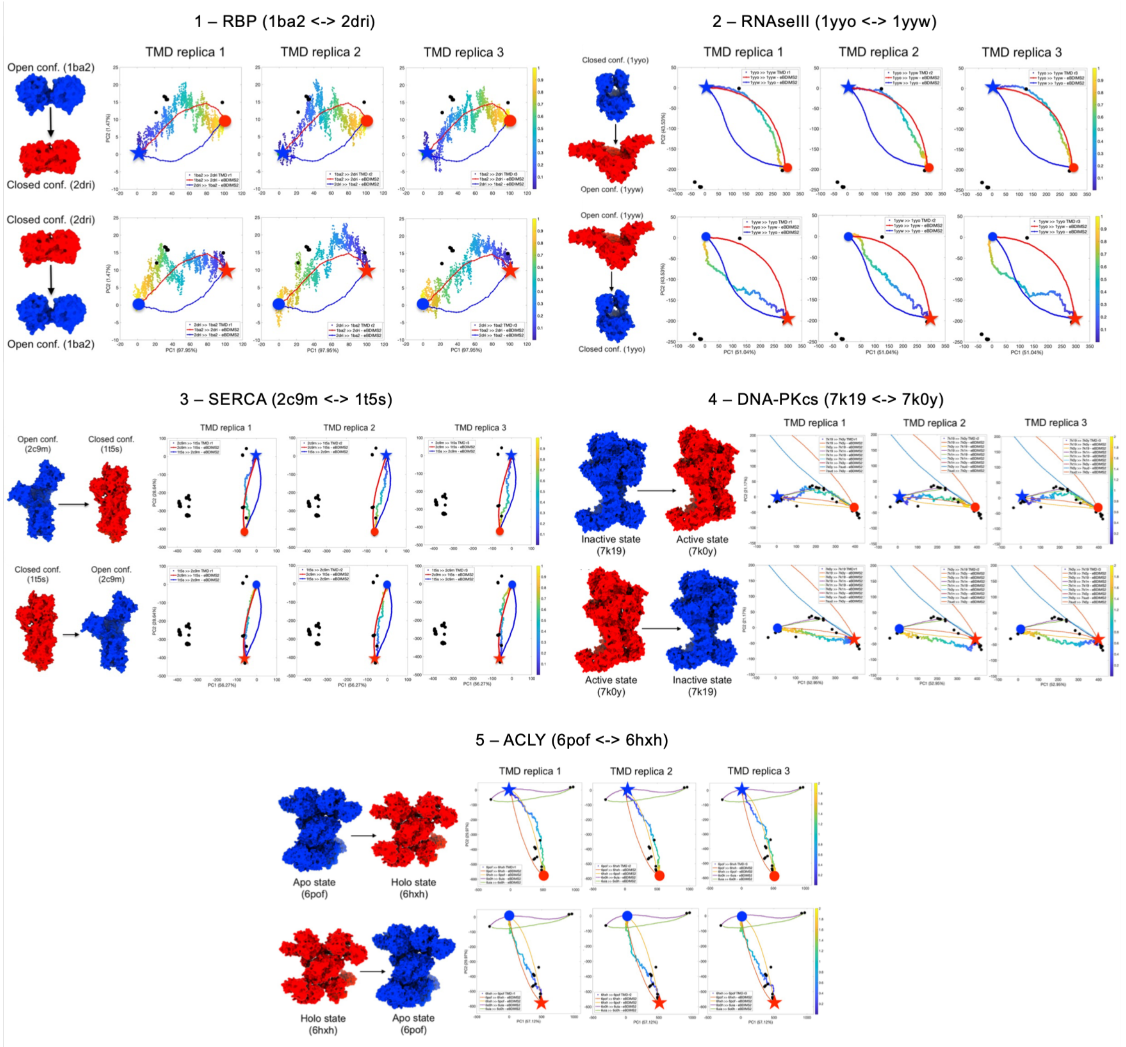

Supplementary Figure 23. PC projections of the TMD simulations for: **(1)** RBP, open-closed (1ba2-2dri) and closed-open transition (2dri-1ba2); **(2)** RNaseIII, closed-open (1yyo-1yyw) and open-closed transition (1yyw-1yyo); **(3)** SERCA, open-closed (2c9m-1t5s) and closed-open transition (1t5s-2c9m); **(4)** DNA-PKcs, inactive-active (7k19-7k0y) and active-inactive transition (7k0y-7k19); **(5)** ACLY, apo-holo (6pof-6hxh) and holo-apo transition (6hxh-6pof). Colored stars and circles refer to the starting and target TMD conformations, respectively. Scattered points are the projections of the TMD trajectories from 0 ns (blue) to 1-2 ns (yellow).

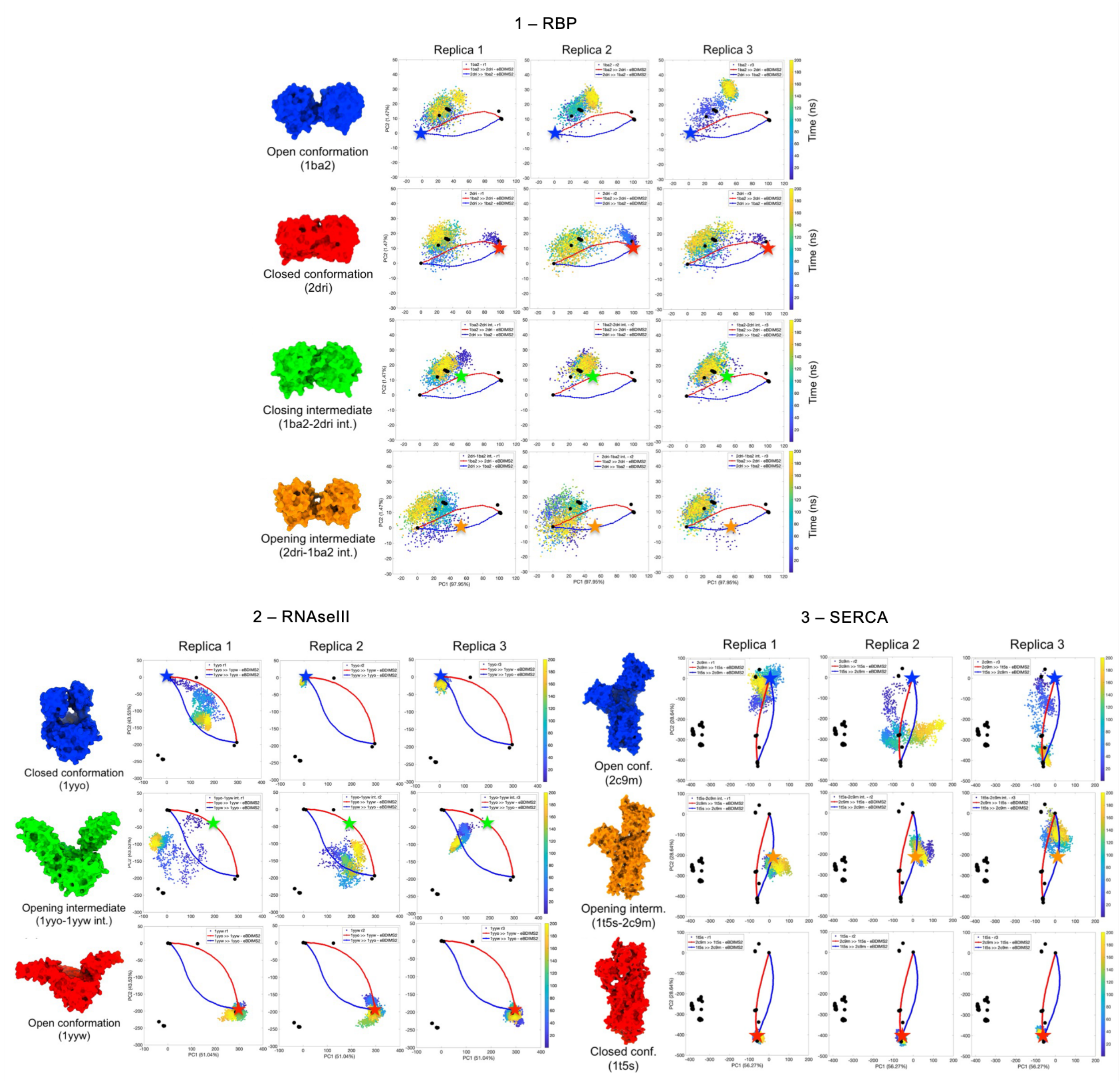

Supplementary Figure 24. PC projections of the unbiased MD simulations for: **(1)** RBP starting from open conformation (1ba2, blue), closed conformation (2dri, red), eBDIMS2 mid-point intermediate in the closing pathway (1ba2-2dri, green), and eBDIMS2 intermediate in the opening pathway (2dri-1ba2, orange); **(2)** RNaseIII starting from closed conformation (1yyo, blue), open conformation (1yyw, red), and eBDIMS2 mid-point intermediate in the opening pathway (1yyo-1yyw, green); **(3)** SERCA starting from open conformation (2c9m, blue), closed conformation (1t5s, red), and eBDIMS2 mid-point intermediate in the opening pathway (1t5s-2c9m, green). Colored stars refer to the starting seeds for MD simulations, and scattered points are the projections of the trajectories from 0 ns (blue) to 200 ns (yellow).

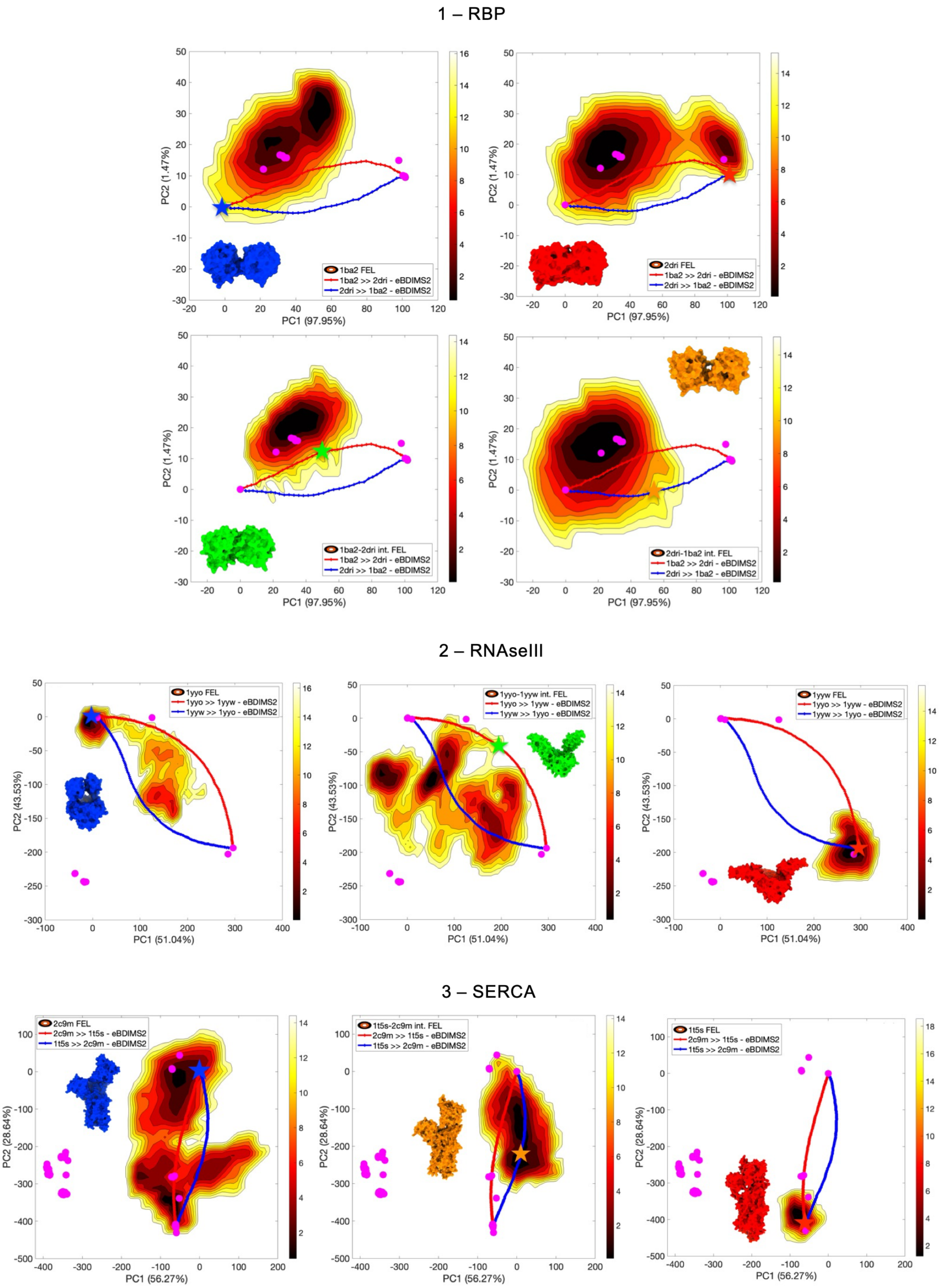

Supplementary Figure 25. PC projections of the FELs of unbiased MD simulations for: **(1)** RBP starting from open conformation (blue), closed conformation (red), eBDIMS2 mid-point intermediate in the open-closed pathway (green), and eBDIMS2 intermediate in the closed-open pathway (orange); **(2)** RNaseIII starting from closed conformation (blue), eBDIMS2 mid-point intermediate in the closed-open pathway (green), and open conformation (red); **(3)** SERCA starting from open conformation (blue), eBDIMS2 mid-point intermediate in the closed-open pathway (orange), and closed conformation (red). Energy values are expressed in kJ/mol.

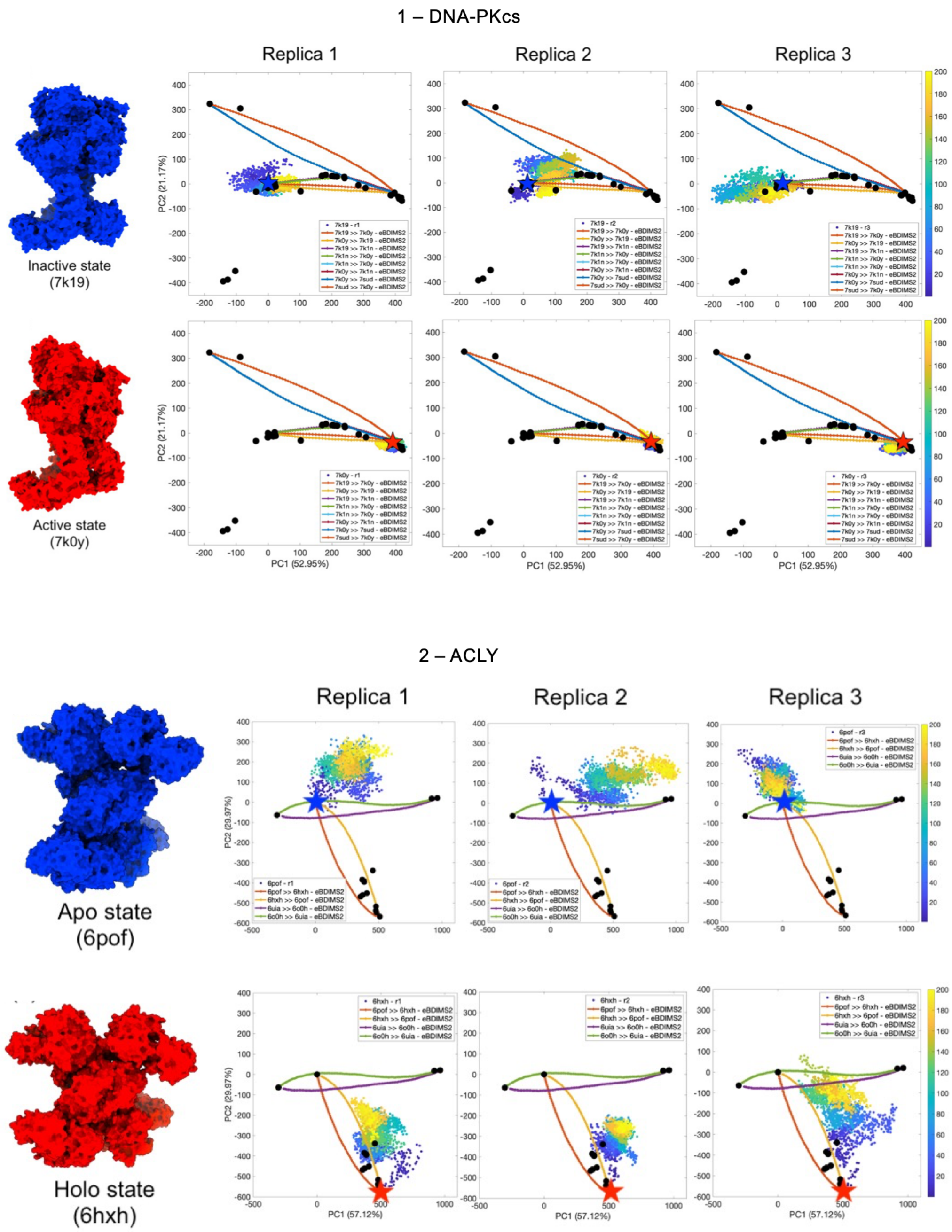

Supplementary Figure 26. PC projections of the unbiased MD simulations for: **(1)** DNA-PKcs starting from inactive conformation (7k19, blue), and active conformation (7k0y, red); **(2)** ACLY starting from apo state (6pof, blue), and holo conformation (6hxh, red). Colored stars refer to the starting seeds for MD simulations, and scattered points are the projections of the trajectories from 0 ns (blue) to 200 ns (yellow).

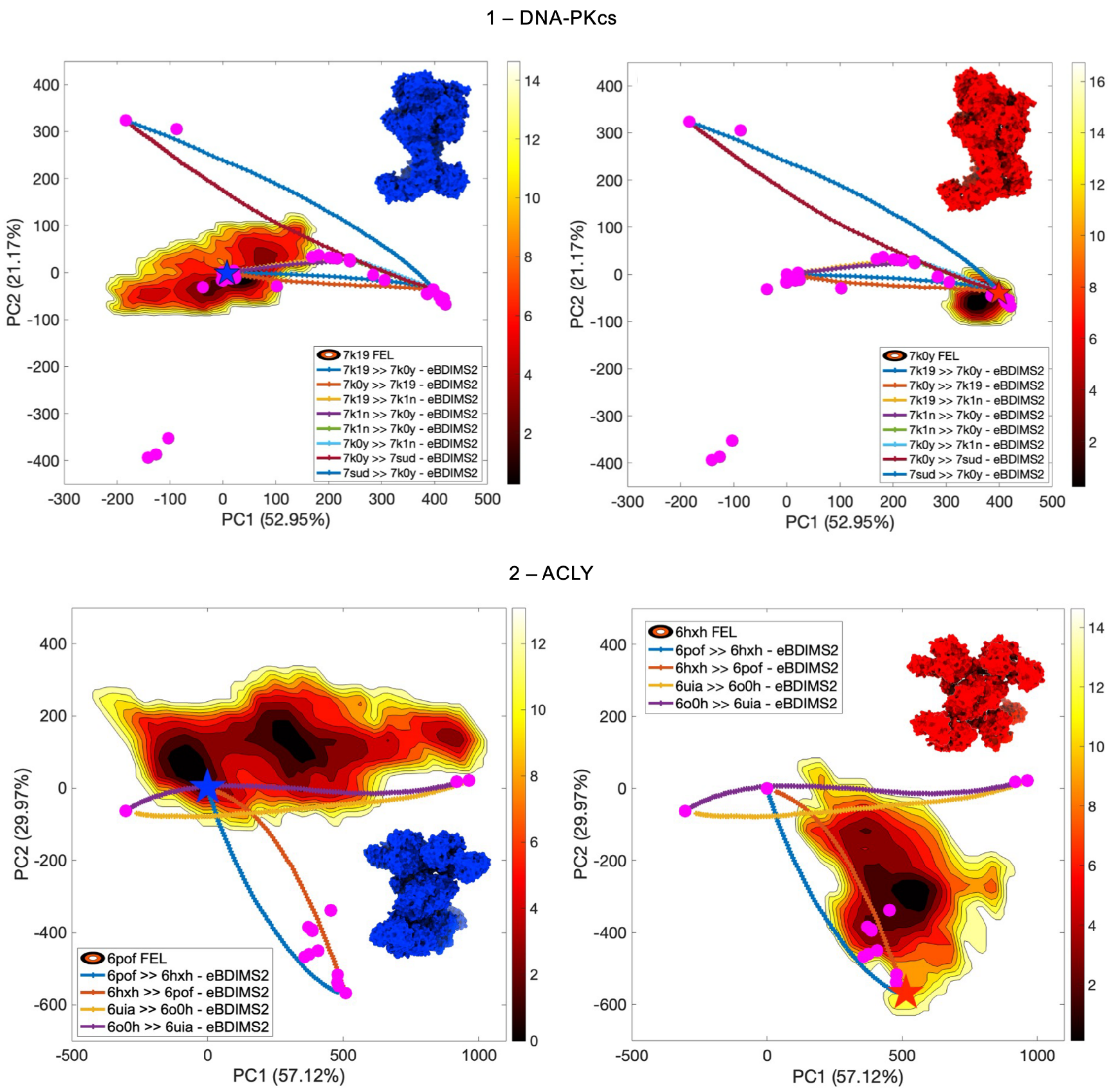

Supplementary Figure 27. PC projections of the FELs of unbiased MD simulations for: **(1)** DNA-PKcs starting from inactive conformation (blue), and active conformation (red); **(2)** ACLY starting from apo state (blue), and holo state (red). Energy values are expressed in kJ/mol.

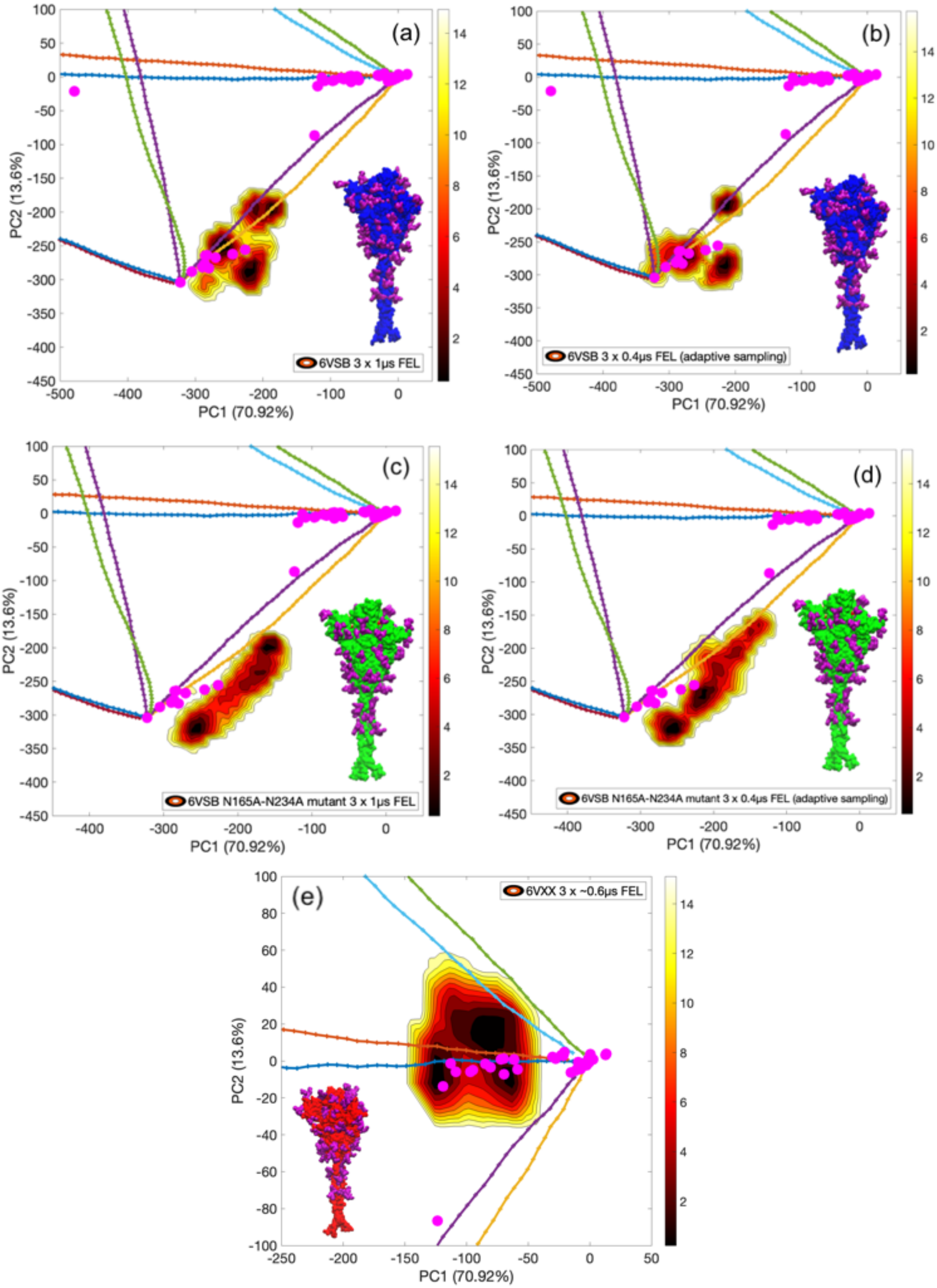

Supplementary Figure 28. PC projections of the FELs of: **(a)** unbiased and **(b)** adaptive sampling MD simulations for *SARS-CoV-2* spike glycoprotein in the open conformation (PDB: 6vsb); **(c)** unbiased and **(d)** adaptive sampling MD simulations of the open conformation with N165A and N234A mutations; **(e)** unbiased MD simulations of the closed conformation (PDB: 6vxx). All simulations come from the Amaro's lab[30] and energy values are expressed in kJ/mol.

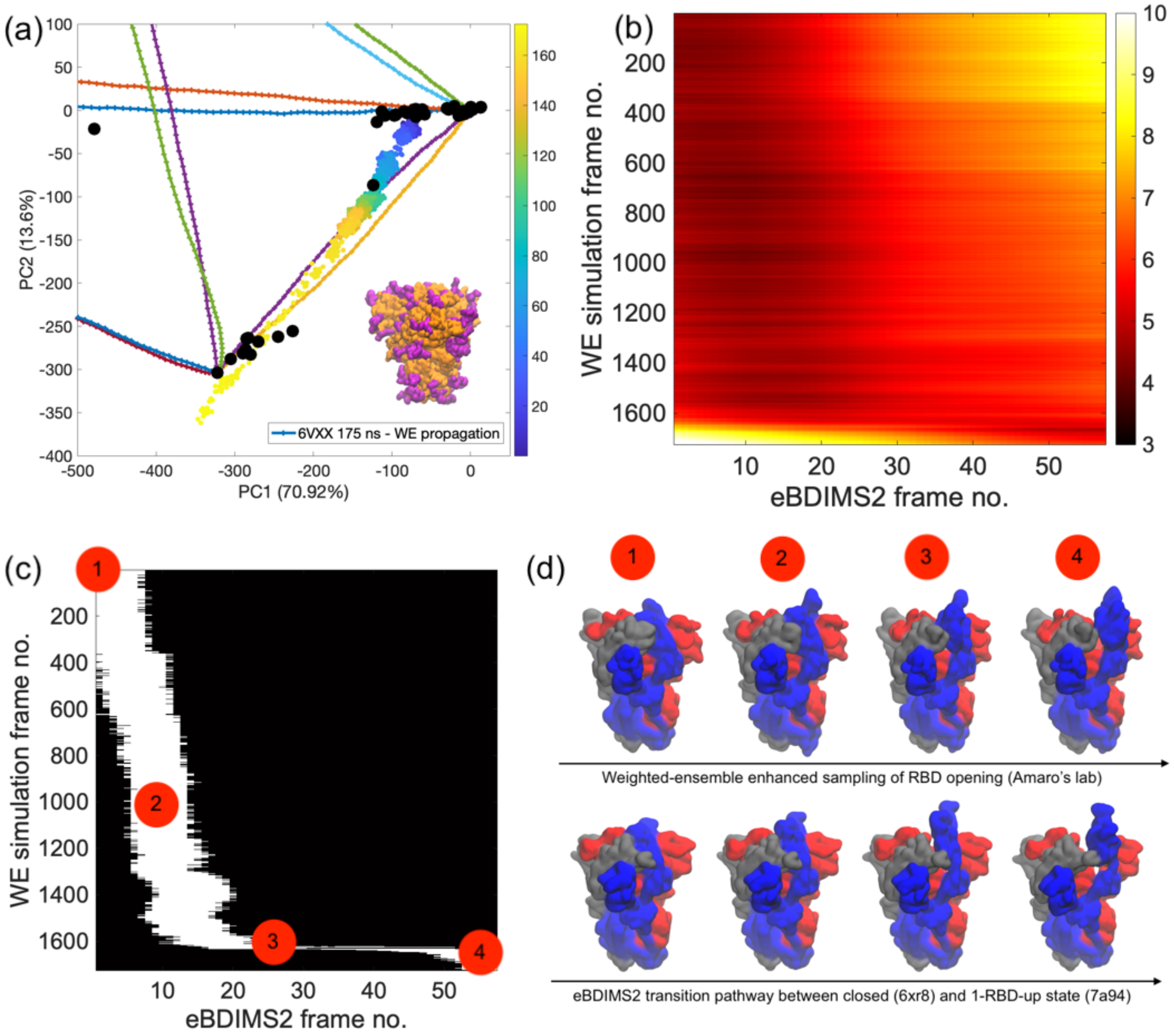

Supplementary Figure 29. Weighted Ensemble (WE) enhanced MD simulation from Amaro's lab[31] for *SARS-CoV-2* spike glycoprotein, starting from the closed state (PDB: 6vxx) and showing full opening of one receptor binding domain (RBP): **(a)** projection of the WE trajectory - from 0 ns (blue points) to 175 ns (bright yellow points) - on a zoomed portion of the experimental PC space; **(b)** RMSD values - from 3 Å (black) to 10 Å (white) - between WE and eBDIMS2 conformers from fully closed state (6xr8) to the 1-RBD-up conformation (7a94); **(c)** RMSD matrix from panel (b), highlighting in white only couples of WE-eBDIMS2 conformations with low RMSD values (RMSD < $RMSD_{min}$ + 2%); **(d)** comparison between four selected conformations along the WE and eBDIMS2 trajectories with minimum pairwise RMSD (~4 Å).

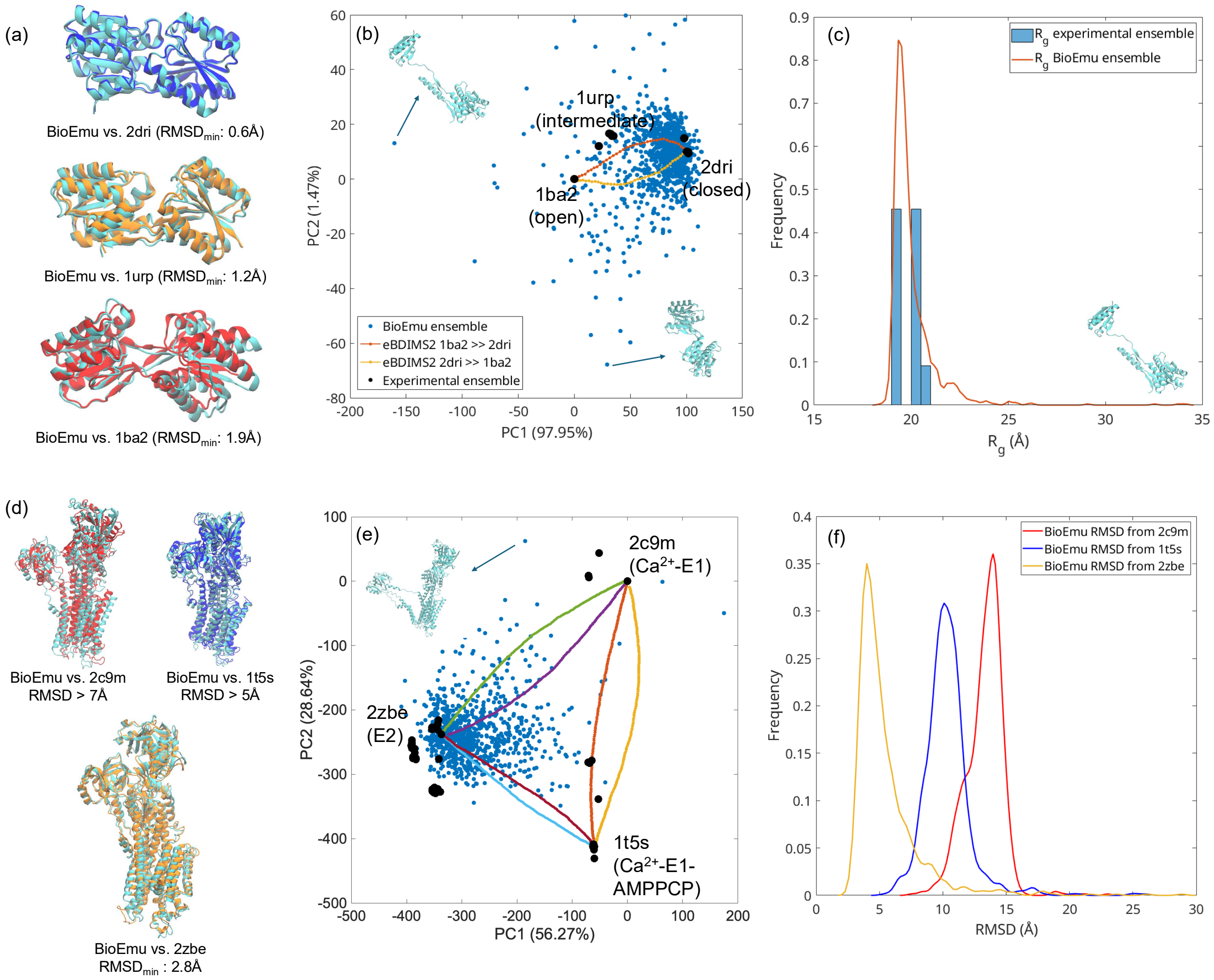

Supplementary Figure 30. Application of BioEmu to **(a-c)** RBP and **(d-f)** SERCA, using default parameters and asking for the generation of 1,000 samples: **(a)** comparison between the BioEmu conformation (cyan) of RBP that is closest to the closed (2dri, blue), intermediate (1urp, orange), and open (1ba2, red) state. RMSD values between BioEmu models and experimental structures are also reported; **(b)** projection of BioEmu predictions (blue points) on the experimental PC space of RBP. Two models with extreme PC1 and PC2 values are shown to highlight their 3D conformations; **(c)** distribution of gyration radii (R_g) of the structures in the experimental ensemble (blue histogram, closed conformations have R_g values of ~19Å, while open states adopt values of ~20/21 Å) and the BioEmu ensemble (red line); **(d)** comparison between the BioEmu conformation (cyan) of SERCA that is closest to the open E1-2Ca^{2+} (2c9m, red), closed E1-2Ca^{2+}-P (1t5s, blue), and closed E2 (2zbe, orange) state; **(e)** projection of BioEmu predictions (blue points) on the experimental PC space of SERCA, together with the three pairs of eBDIMS2 transition pathways between the three end states; **(f)** distribution of RMSD values between all BioEmu predictions and 2c9m (red), 1t5s (blue), and 2zbe (orange).

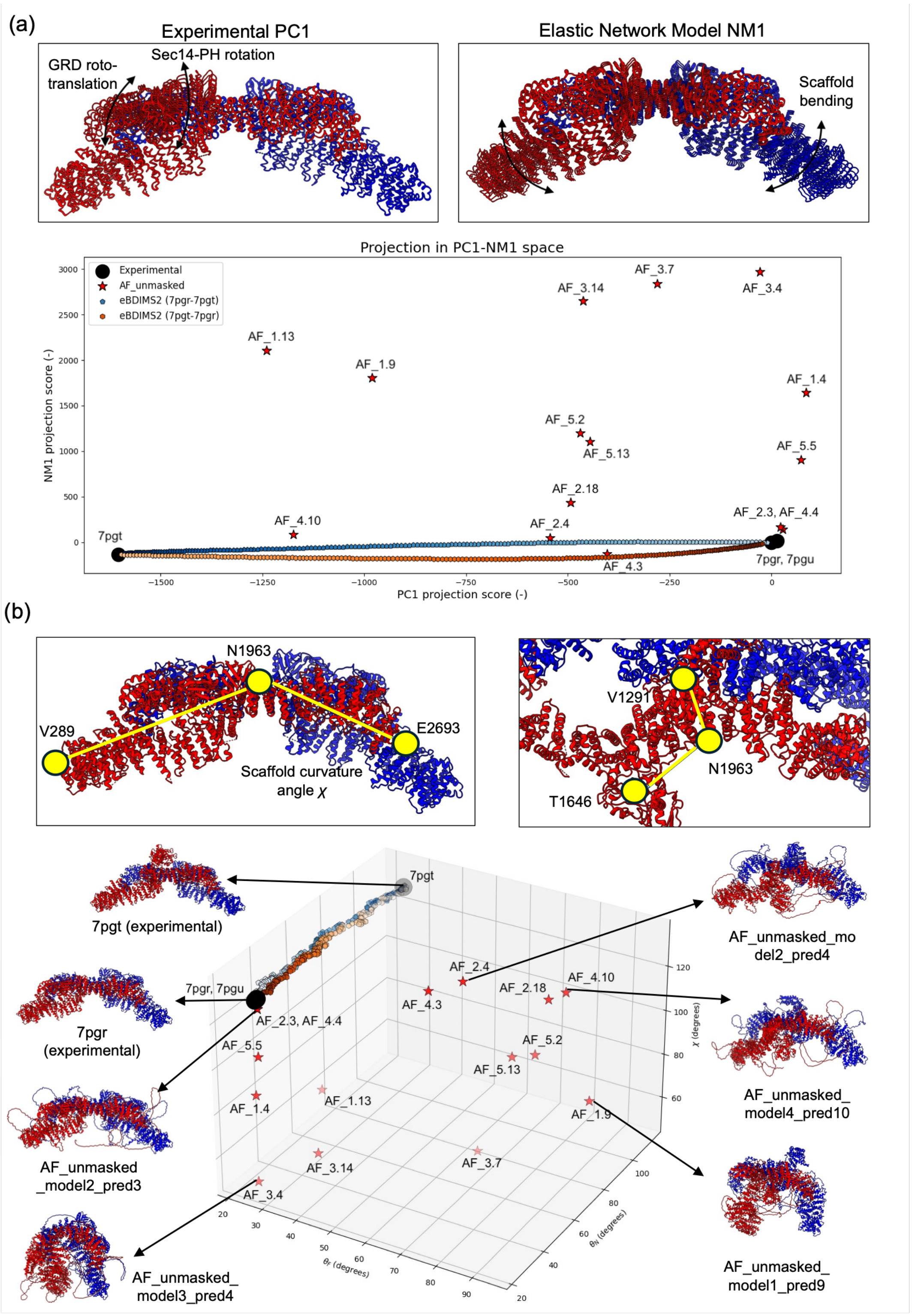

Supplementary Figure 31. Comparison between experimental, eBDIMS2, and AF_unmasked[35] conformers for isoform 2 of neurofibromin (Nf1): **(a)** projection of all conformations in a 2D space defined by the first PC eigenvector (PC1) of the Nf1 ensemble (highlighting the opening-closing motion of the GRD–Sec-14PH domains) and the first normal mode (NM1) computed from the edENM[6] of the closed state (highlighting scaffold bending). Projections of experimental structures are shown with black dots, eBDIMS2 intermediate conformations in the two pathway directions with pentagons and hexagons of decreasing brightness, and AF_unmasked with red stars. The numbers of AF_unmasked labels refer to the model and prediction number as deposited by the authors; **(b)** projection in a 3D space defined by the average scaffold curvature, χ, and the two angles describing the opening-closing motions of the GRD–Sec-14PH domains of the two Nf1 chains, θ_F and θ_N. Some representative models of AF_unmasked conformers, as well as the two experimental end states, are also reported with ribbon representation close to 3D plot.

References

1. Orellana, L., Yoluk, O., Carrillo, O., Orozco, M. & Lindahl, E. Prediction and validation of protein intermediate states from structurally rich ensembles and coarse-grained simulations. *Nat Commun* **7**, 12575 (2016).
2. Berman, H. M. *et al.* The Protein Data Bank. *Nucleic Acids Research* **28**, 235–242 (2000).
3. The UniProt Consortium. UniProt: the Universal Protein Knowledgebase in 2023. *Nucleic Acids Research* **51**, D523–D531 (2023).
4. Zhang, C., Shine, M., Pyle, A. M. & Zhang, Y. US-align: universal structure alignments of proteins, nucleic acids, and macromolecular complexes. *Nat Methods* **19**, 1109–1115 (2022).
5. Abraham, M. J. *et al.* GROMACS: High performance molecular simulations through multi-level parallelism from laptops to supercomputers. *SoftwareX* **1–2**, 19–25 (2015).
6. Orellana, L. *et al.* Approaching Elastic Network Models to Molecular Dynamics Flexibility. *J. Chem. Theory Comput.* **6**, 2910–2923 (2010).
7. Orellana, L., Gustavsson, J., Bergh, C., Yoluk, O. & Lindahl, E. eBDIMS server: protein transition pathways with ensemble analysis in 2D-motion spaces. *Bioinformatics* **35**, 3505–3507 (2019).
8. Scaramozzino, D., Lee, B. H. & Orellana, L. eBDIMS2 large protein motion benchmark dataset. figshare https://doi.org/10.6084/m9.figshare.29423201.v1 (2025).
9. Tama, F. & Sanejouand, Y.-H. Conformational change of proteins arising from normal mode calculations. *Protein Engineering, Design and Selection* **14**, 1–6 (2001).
10. Weiss, D. R. & Levitt, M. Can Morphing Methods Predict Intermediate Structures? *Journal of Molecular Biology* **385**, 665–674 (2009).
11. Zheng, W. & Wen, H. A survey of coarse-grained methods for modeling protein conformational transitions. *Current Opinion in Structural Biology* **42**, 24–30 (2017).
12. Lopéz-Blanco, J. R., Garzón, J. I. & Chacón, P. iMod: multipurpose normal mode analysis in internal coordinates. *Bioinformatics* **27**, 2843–2850 (2011).
13. Sfriso, P., Hospital, A., Emperador, A. & Orozco, M. Exploration of conformational transition pathways from coarse-grained simulations. *Bioinformatics* **29**, 1980–1986 (2013).
14. Lee, B. H. *et al.* Normal mode-guided transition pathway generation in proteins. *PLoS ONE* **12**, e0185658 (2017).
15. Lee, B. H., Park, S. W., Jo, S. & Kim, M. K. Protein conformational transitions explored by a morphing approach based on normal mode analysis in internal coordinates. *PLoS ONE* **16**, e0258818 (2021).
16. Grudinin, S., Laine, E. & Hoffmann, A. Predicting Protein Functional Motions: an Old Recipe with a New Twist. *Biophysical Journal* **118**, 2513–2525 (2020).
17. Kim, M. K., Chirikjian, G. S. & Jernigan, R. L. Elastic models of conformational transitions in macromolecules. *Journal of Molecular Graphics and Modelling* **21**, 151–160 (2002).
18. Yang, Z., Májek, P. & Bahar, I. Allosteric Transitions of Supramolecular Systems Explored by Network Models: Application to Chaperonin GroEL. *PLoS Comput Biol* **5**, e1000360 (2009).
19. Das, A. *et al.* Exploring the Conformational Transitions of Biomolecular Systems Using a Simple Two-State Anisotropic Network Model. *PLoS Comput Biol* **10**, e1003521 (2014).
20. Koehl, P., Navaza, R., Tekpinar, M. & Delarue, M. MinActionPath2: path generation between different conformations of large macromolecular assemblies by action minimization. *Nucleic Acids Research* **52**, W256–W263 (2024).
21. Franklin, J., Koehl, P., Doniach, S. & Delarue, M. MinActionPath: maximum likelihood trajectory for large-scale structural transitions in a coarse-grained locally harmonic energy landscape. *Nucleic Acids Research* **35**, W477–W482 (2007).
22. Naschberger, A., Baradaran, R., Rupp, B. & Carroni, M. The structure of neurofibromin isoform 2 reveals different functional states. *Nature* **599**, 315–319 (2021).
23. Heo, L. & Feig, M. One bead per residue can describe all-atom protein structures. *Structure* **32**, 97-111.e6 (2024).
24. Williams, C. J. *et al.* MolProbity: More and better reference data for improved all-atom structure validation. *Protein Sci* **27**, 293–315 (2018).
25. Bergh, C., Heusser, S. A., Howard, R. & Lindahl, E. Markov state models of proton- and pore-dependent activation in a pentameric ligand-gated ion channel. *eLife* **10**, e68369 (2021).

26. Schlitter, J., Engels, M. & Krüger, P. Targeted molecular dynamics: a new approach for searching pathways of conformational transitions. *J Mol Graph* **12**, 84–89 (1994).
27. Waterhouse, A. *et al.* SWISS-MODEL: homology modelling of protein structures and complexes. *Nucleic Acids Research* **46**, W296–W303 (2018).
28. Daidone, I. & Amadei, A. Essential dynamics: foundation and applications. *WIREs Comput Mol Sci* **2**, 762–770 (2012).
29. Mhashal, A., Emperador, A. & Orellana, L. Computational techniques to study protein dynamics and conformations. in *Advances in Protein Molecular and Structural Biology Methods* 199–212 (Elsevier, 2022). doi:10.1016/B978-0-323-90264-9.00013-1.
30. Casalino, L. *et al.* Beyond Shielding: The Roles of Glycans in the SARS-CoV-2 Spike Protein. *ACS Cent. Sci.* **6**, 1722–1734 (2020).
31. Casalino, L. *et al.* AI-driven multiscale simulations illuminate mechanisms of SARS-CoV-2 spike dynamics. *The International Journal of High Performance Computing Applications* **35**, 432–451 (2021).
32. Zuckerman, D. M. & Chong, L. T. Weighted Ensemble Simulation: Review of Methodology, Applications, and Software. *Annual Review of Biophysics* **46**, 43–57 (2017).
33. Jumper, J. *et al.* Highly accurate protein structure prediction with AlphaFold. *Nature* **596**, 583–589 (2021).
34. Lewis, S. *et al.* Scalable emulation of protein equilibrium ensembles with generative deep learning. Preprint at https://doi.org/10.1101/2024.12.05.626885 (2024).
35. Mirabello, C., Wallner, B., Nystedt, B., Azinas, S. & Carroni, M. Unmasking AlphaFold to integrate experiments and predictions in multimeric complexes. *Nat Commun* **15**, 8724 (2024).
36. Lewis, S. *et al.* Supplementary data for 'scalable emulation of protein equilibrium ensembles with generative deep learning'. https://doi.org/10.5281/zenodo.15672282 (2025) doi:10.5281/zenodo.15672282.
37. Mirabello, C., Wallner, B., Nystedt, B. & Carroni, M. AlphaFold Unmasked data sets. https://doi.org/10.17044/scilifelab.24198669.v1 (2024).
38. Des Georges, A. *et al.* Structural Basis for Gating and Activation of RyR1. *Cell* **167**, 145-157.e17 (2016).
