# Supplementary material for: Efficient sampling of large-scale transition pathways and intermediate conformations in sub-mesoscopic protein complexes
Source: Nat Commun. 2026 Mar 2;17:2202. doi: 10.1038/s41467-026-69809-y (PMC12960823; doi:10.1038/s41467-026-69809-y)
Supplement: Supplementary file 2 — Description of Additional Supplementary File [file 41467_2026_69809_MOESM2_ESM.pdf]

The Description of Additional Supplementary Files

Supplementary Movie 1. Opening-closing movements in the GroEL chaperonin.

Supplementary Movie 2. Opening-closing movements in DNAPKCs.

Supplementary Movie 3. Opening-closing movements in SARS-CoV-2 spike protein.

Supplementary Movie 4. Opening-closing movements in A2M.

Supplementary Movie 5. Full rotary cycle of ATP synthase.

Supplementary Movie 6. GRD-Sec14-PH roto-translations in Neurofibromin 1.

Supplementary Movie 7. Large-scale rearrangement in the LRP2 dimer.

Supplementary Movie 8. Shear transition in the ring-shaped Mpeg1 pore.

Supplementary Movie 9. Torsional twist in the megadalton-sized Lambda tail tip complex.
