# Supplementary figures and images for: Efficient sampling of large-scale transition pathways and intermediate conformations in sub-mesoscopic protein complexes
Source: Nat Commun. 2026 Mar 2;17:2202. doi: 10.1038/s41467-026-69809-y (PMC12960823; doi:10.1038/s41467-026-69809-y)

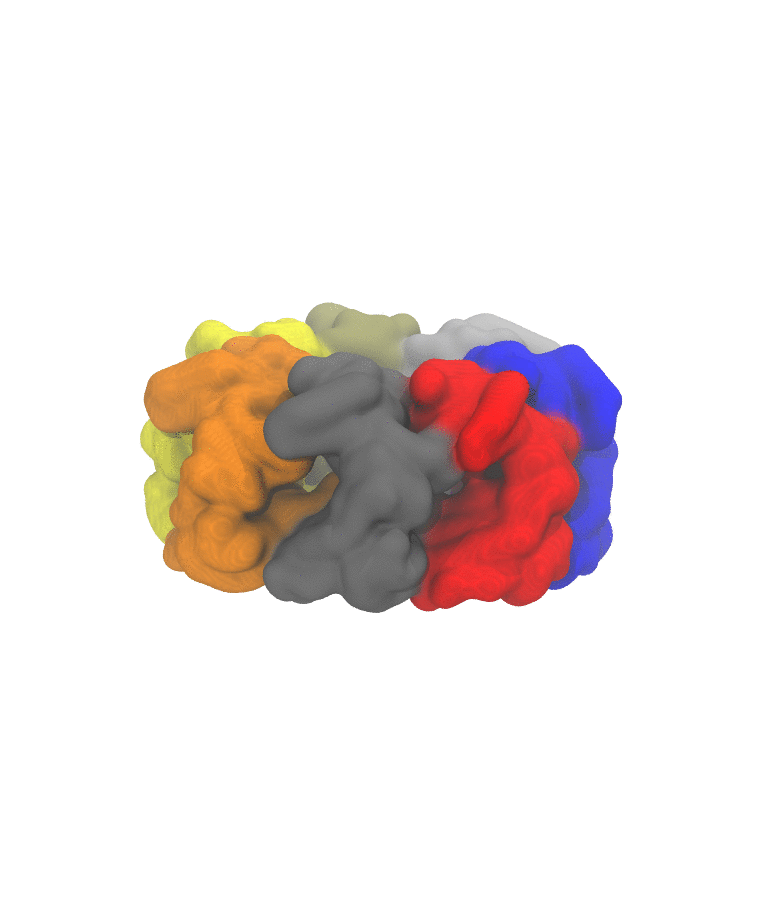

Supplement: Supplementary file 3 — Supplementary Movie 1 [file 41467_2026_69809_MOESM3_ESM.gif]

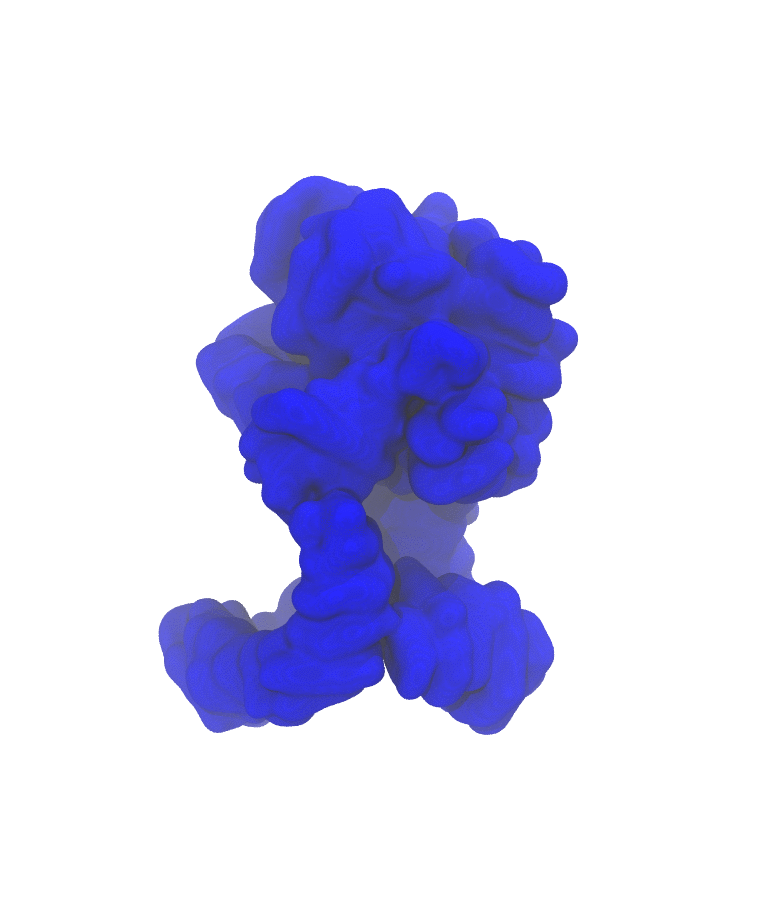

Supplement: Supplementary file 4 — Supplementary Movie 2 [file 41467_2026_69809_MOESM4_ESM.gif]

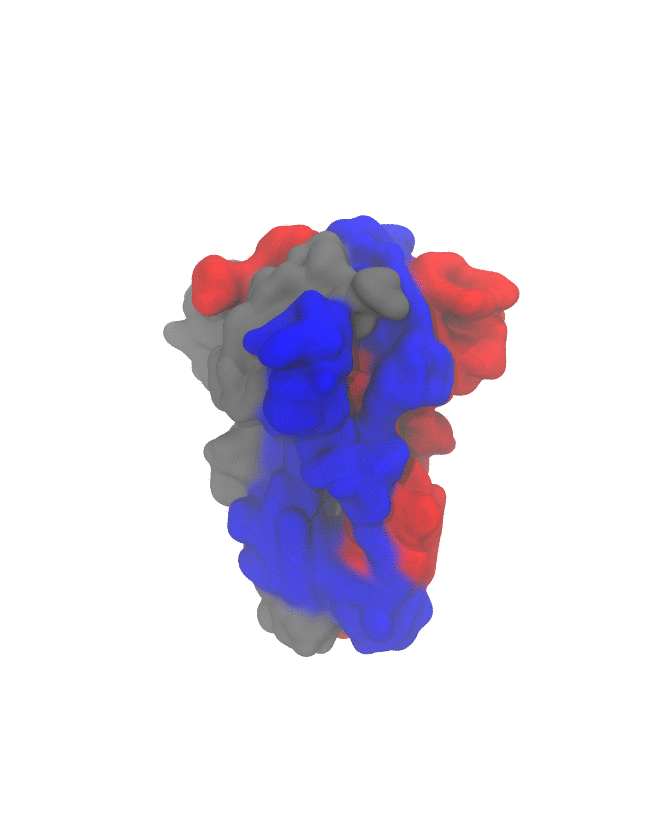

Supplement: Supplementary file 5 — Supplementary Movie 3 [file 41467_2026_69809_MOESM5_ESM.gif]

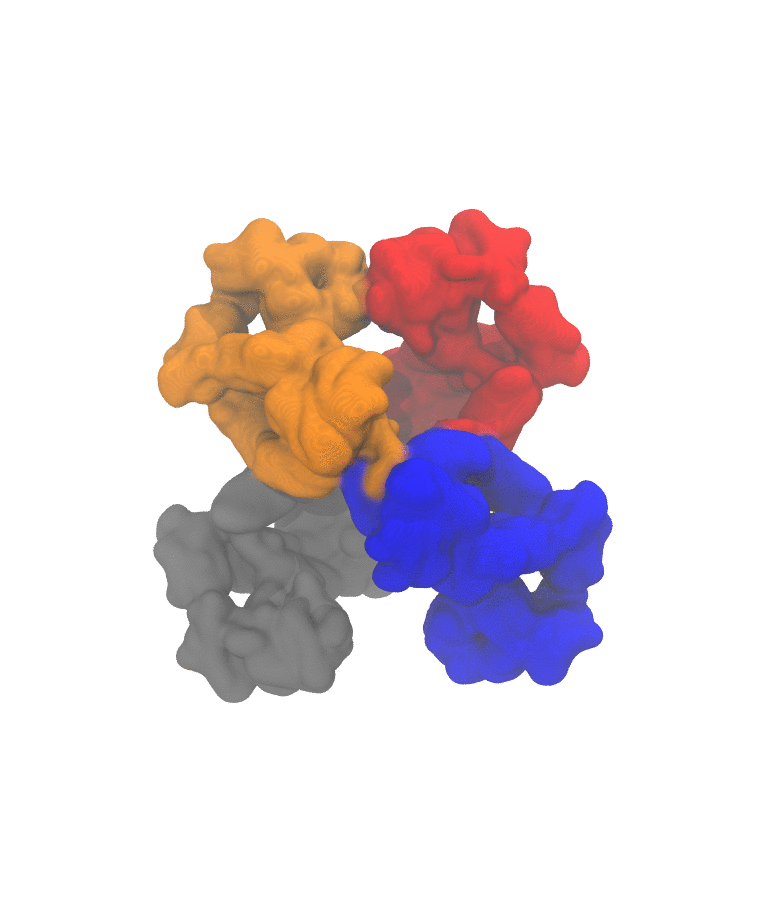

Supplement: Supplementary file 6 — Supplementary Movie 4 [file 41467_2026_69809_MOESM6_ESM.gif]

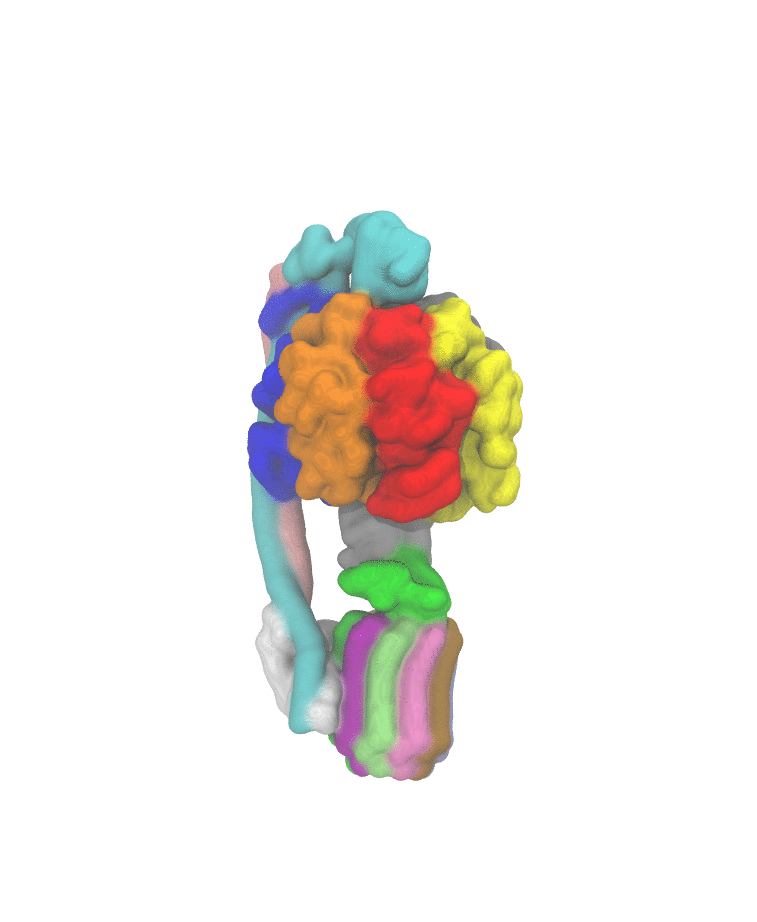

Supplement: Supplementary file 7 — Supplementary Movie 5 [file 41467_2026_69809_MOESM7_ESM.gif]

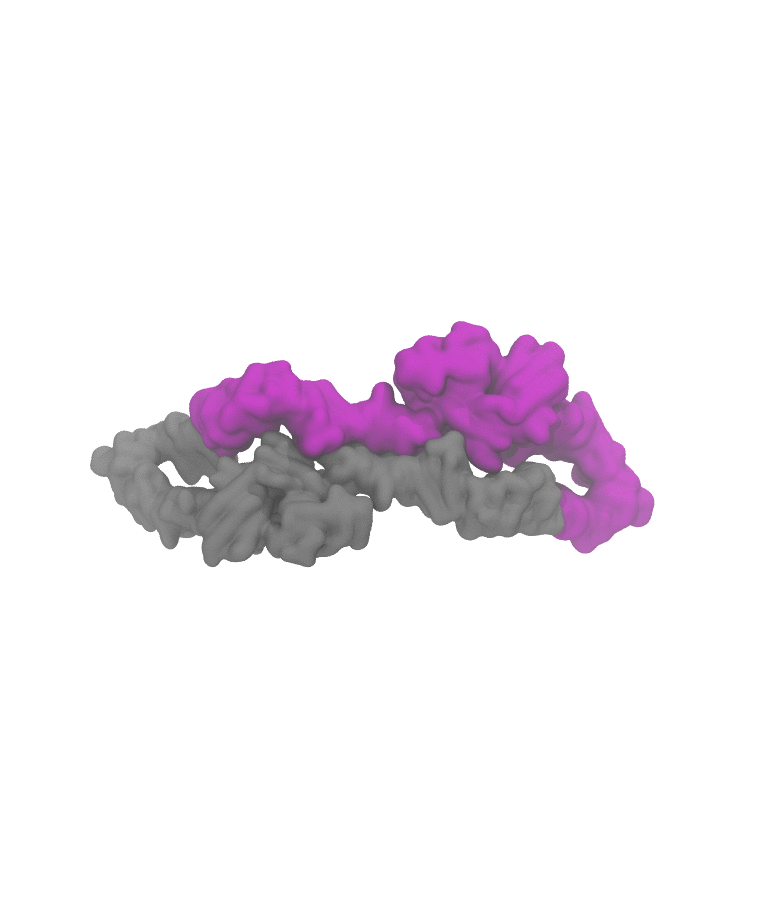

Supplement: Supplementary file 8 — Supplementary Movie 6 [file 41467_2026_69809_MOESM8_ESM.gif]

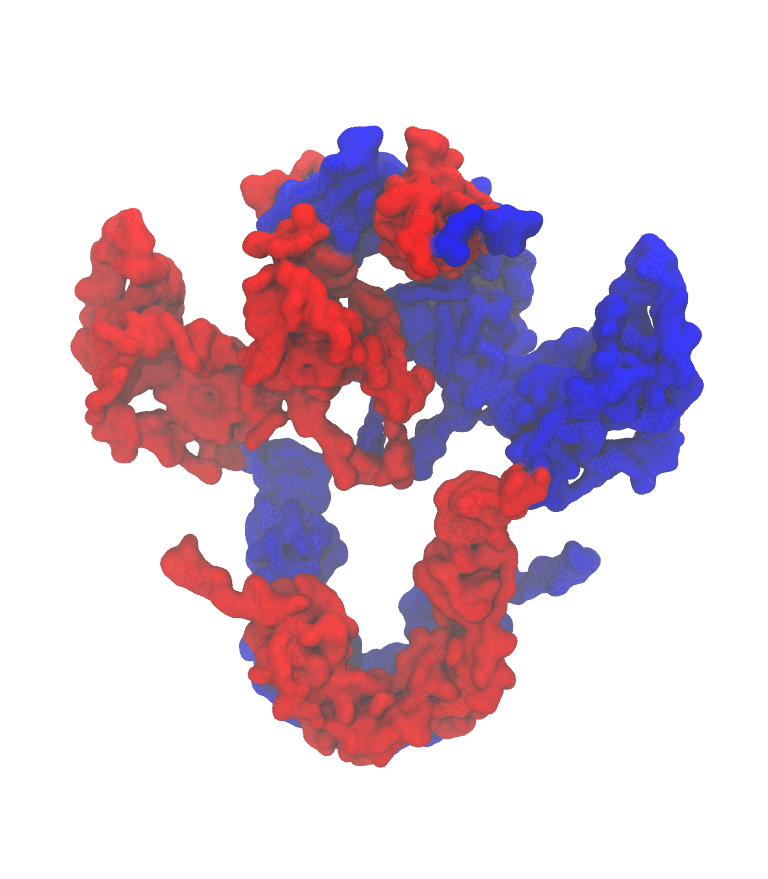

Supplement: Supplementary file 9 — Supplementary Movie 7 [file 41467_2026_69809_MOESM9_ESM.gif]

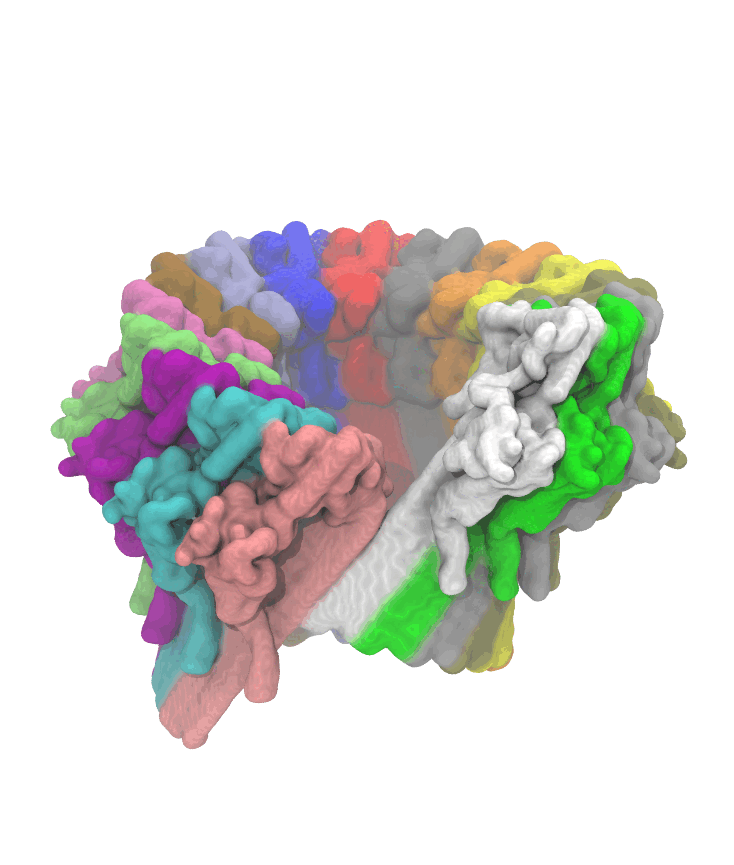

Supplement: Supplementary file 10 — Supplementary Movie 8 [file 41467_2026_69809_MOESM10_ESM.gif]

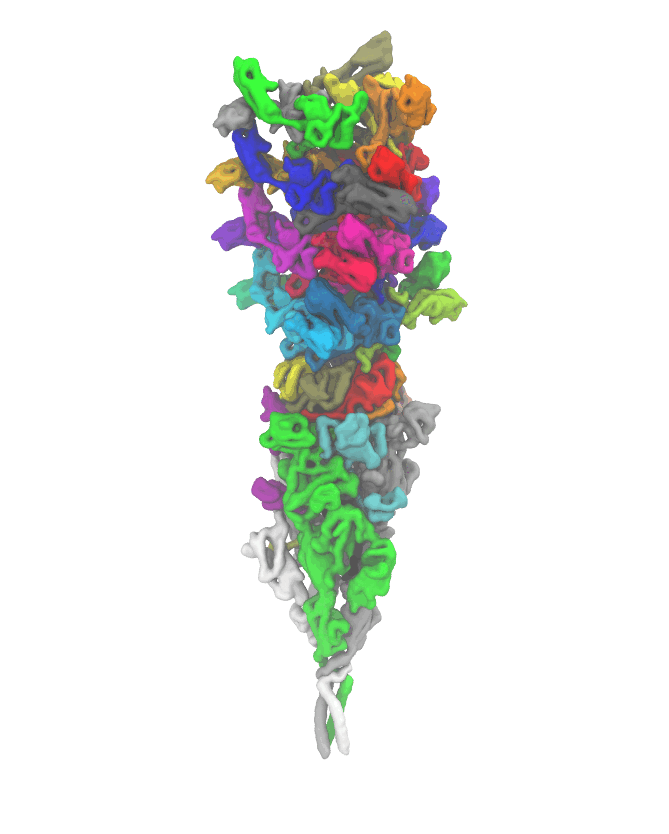

Supplement: Supplementary file 11 — Supplementary Movie 9 [file 41467_2026_69809_MOESM11_ESM.gif]
